# Synchronous wearable ultrasound for early detection of coronary and carotid artery comorbidity

**DOI:** 10.1126/sciadv.aed2114

**Published:** 2026-06-19

**Authors:** Shengrong Lin, Hao Xu, Ya Li, Xinyang Ge, Kang Chen, Lin Chen, Minfeng Tong, Fangyi Wang, Dawei Wu, Hongxiang Lin, Wenli Dai, Yingying Zhang, Yuxin Xing, Jianming Wen, Dexing Kong

**Affiliations:** ^1^ College of Mathematical Medicine, Zhejiang Normal University, Zhejiang 321004, China.; ^2^ College of Computer Science, Zhejiang Normal University, Zhejiang 321004, China.; ^3^ Department of Neurosurgery, Affiliated Jinhua Hospital, Zhejiang University School of Medicine, Zhejiang 321004, China.; ^4^ State Key Laboratory of Mechanics and Control for Aerospace Structures, Nanjing University of Aeronautics and Astronautics, Nanjing 210016, China.; ^5^ Medical Engineering & Engineering Medicine Innovation Center, Hangzhou International Innovation Institute, Beihang University, Zhejiang 311115, China.; ^6^ Key Laboratory of Biomechanics and Mechanobiology, Ministry of Education, Beijing Advanced Innovation Center for Biomedical Engineering, School of Biological Science and Medical Engineering, Beihang University, Beijing 100191, China.; ^7^ School of Mathematical Sciences, Zhejiang University, Zhejiang 321004, China.; ^8^ Institute of Pathology and Southwest Cancer Center, Southwest Hospital of the Third Military Medical University, Chongqing 400038, Sichuan, China.

## Abstract

Coronary heart disease (CHD) and carotid artery disease (CAD) often co-occur. However, conventional diagnosis typically involves separate, site-by-site examinations after symptoms appear, leading to delayed intervention. In this work, we developed a wearable ultrasound system that enables synchronous monitoring of cardiac and carotid dynamics for comorbidity assessment. The system combines dual wearable ultrasound patches, a synchronous imaging strategy, artificial intelligence–based image processing algorithms, and human circuitry models to automatically extract and analyze key cardiac-carotid metrics, such as heart rate, pulse rate, cardiac volume, cardiac output, and carotid blood pressure. By evaluating the correlation of these metrics between modeling and measurements, we showed the feasibility of differentiating among healthy participants and patients with CAD, CHD, or CAD-CHD comorbidity. This integrated approach constitutes a promising framework for supporting the proactive assessment of coronary-carotid comorbidity.

## Teaser

A wearable ultrasound system enables synchronized cardiac and carotid imaging for comorbidity evaluation.

## INTRODUCTION

Coronary heart disease (CHD) and carotid artery disease (CAD) are prevalent manifestations of systemic atherosclerosis, a chronic inflammatory condition characterized by the buildup of plaque within arteries (*1*, *2*). Statistics suggest that in patients with CHD, 25.4% were found to have CAD exceeding 50% stenosis upon ultrasound examination, with the detection rate positively correlating to the number of affected coronary vessels; furthermore, the prevalence of CHD among patients with CAD ranges from 13 to 86% (*3*). The frequent coexistence of these two conditions is not coincidental but rather indicative of a shared pathophysiology (*4*, *5*). This comorbidity presents a critical challenge in clinical management, as patients with both conditions represent a high-risk population for major adverse cardiovascular and cerebrovascular events.

There is currently no single standard test for diagnosing the comorbidity of CHD and CAD simultaneously (*6*, *7*). Instead, the diagnosis relies on the separate assessment of each vascular bed using its own gold standard modality (Supplementary Text), followed by clinical correlation to establish comorbidity (*8*, *9*). This diagnostic approach is typically triggered by symptoms or the identification of disease in one territory, prompting investigation in the other. Specifically, invasive coronary angiography is the gold standard for diagnosing CHD (*10*, *11*), which provides high-resolution visualization of the coronary lumen, and precisely quantifying the location, severity, and characteristics of stenoses; color-doppler ultrasonography is the first-line screening tool for CAD (*12*, *13*), which is noninvasive and cost-effective for measuring stenosis severity via characterizing plaque morphology. These conventional diagnostic methods face three principal limitations. First, they are typically reactive, initiated only after symptom onset, often missing the critical window for early intervention. Second, these techniques commonly provide a static, resting-state assessment, failing to capture pathological dynamics under physiological stress, which is when conditions like coronary ischemia often manifest (*14*, *15*). Third, they are inherently not well suited for continuous and wearable monitoring, which is crucial for early detection of the disease or managing its prognosis where silent plaque rupture can lead to acute events like myocardial infarction or stroke (*16*).

Wearable ultrasound has emerged as a transformative modality, enabling continuous, noninvasive, and hands-free monitoring of deep physiological dynamics (*17*). This paradigm shift from intermittent, static imaging to persistent assessment has been successfully demonstrated across multiple critical vascular and organ systems. Pioneering studies have validated its use for tracking central hemodynamics through continuous imaging of the carotid artery (*18*), as well as for assessing cardiac function (*19*), cerebral perfusion, and brain activity (*20*, *21*) and tracking blood pressure fluctuations (*22*–*24*). Beyond fundamental anatomical imaging, the integration of advanced functionalities such as Doppler flowmetry (*25*) for quantifying blood velocity, photoacoustic imaging (*26*) for mapping hemodynamic biomarkers like hemoglobin concentration, and elastography (*27*) for assessing tissue mechanical properties, notably enriches its diagnostic utility. This convergence of continuous data acquisition with multiparametric imaging capabilities positions wearable ultrasound as a powerful tool for comprehensive, dynamic physiological monitoring. Given the fact that conventional handheld ultrasound has contributed to the separate evaluation of cardiac or carotid diseases, such as echocardiography for diagnosing acute myocardial ischemia (*28*, *29*) and carotid ultrasound for assessing carotid stenosis (*12*, *13*), wearable ultrasound would potentially offer uninterrupted monitoring of carotid and coronary status, providing a comprehensive dataset for advancing early detection of the comorbidity.

Here, we present a wearable ultrasound system for synchronous capture of cardiac-carotid dynamics and automatic extraction of clinically important metrics for comorbidity analysis. The core of our method involves the proposed synchronous wearable ultrasound technology, comprising anatomically optimized ultrasound patches, synchronous imaging strategy, artificial intelligence (AI)–powered image processing, and cardiac-carotid–coupled circuitry models. We demonstrated the feasibility of the proposed method in differentiating among healthy participants, patient with CAD, patient with CHD, and patient with CAD-CHD comorbidity. Accordingly, this method can potentially serve as a diagnostic aid to suggest the possible presence or early signs of comorbidity, with subsequent confirmation to be pursued via clinical standard procedures.

## RESULTS

### Monitoring strategies by synchronous wearable ultrasound

**Fig. 1.**
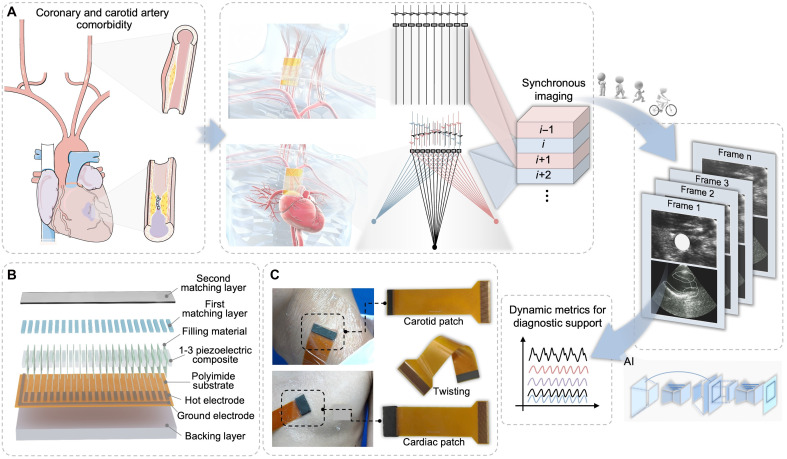
Concept of synchronous wearable ultrasound for evaluation of concomitant coronary and carotid artery disease. (**A**) Schematics showing concomitant coronary and carotid artery disease (left), synchronous wearable ultrasound imaging (middle), capture of frame-by-frame ultrasound images under different physiological conditions (right), and extraction of clinically important dynamic metrics with AI algorithms for supporting diagnosis (right bottom). In this work, the developed system typically achieves an imaging frame rate of 15 to 20 Hz, with a temporal offset of ~2.5 ± 0.5 ms between acquired cardiac and carotid images within a single frame. (**B**) Exploded view of the developed wearable ultrasound patch, with the key components labeled on the right. (**C**) Photos of the patches secured to the skin by adhesive tapes and optical images of the cardiac and carotid patches. Panel (A) was created using C4D and Photoshop software.

Conventional diagnostic methods are typically conducted site by site using separate modalities and mainly focus on resting state metrics initiated after symptomatic individuals seek medical attention (Supplementary Text), which thus may lead to delayed intervention. The proposed concept of synchronous wearable ultrasound for evaluating CHD and CAD comorbidity is illustrated in Fig. 1A. We propose to synchronously capture cardiac and carotid images under physiological stress. The resulting data are then processed by AI algorithms to extract clinically important cardiac-carotid metrics. These metrics are further input into a computational cardiac-carotid circuitry model. Last, comorbidity status is evaluated by fine-tuning the model’s pathological simulations to align with the measured data.

We implemented a stress echocardiography protocol using a graded bicycle exercise regimen (table S1) (*30*). This method captures comprehensive cardiac-carotid data across a broad physiological range, from rest to peak exercise, thereby unmasking subclinical disease not apparent at rest (Supplementary Text). Clinically, the protocol has proven effective for diagnosing acute myocardial ischemia, demonstrating a sensitivity of 80 to 86% and a specificity of 84 to 92% (*28*, *29*). For a preclinical demonstration, we recruited two participant groups (Materials and Methods). The first group comprised five healthy participants who completed the graded bicycle exercise protocol, while the second group included three patients with confirmed CAD, CHD, or CAD-CHD comorbidity that were assessed at rest for safety considerations. A synchronous ultrasound imaging strategy was implemented for three primary reasons. First, it enables the simultaneous acquisition of cardiac and carotid data within a single test, thereby reducing procedural complexity. Second, during graded exercise, the heart rate (HR) increases substantially; if carotid and cardiac data are not synchronized, the derived transient metrics become misaligned in the time domain, complicating their interpretation. Third, the developed human circuitry model uses measured cardiac data as input to output additional cardiac-carotid metrics for further evaluation; synchronous data are essential to ensure the accuracy of this model-based interpretation. The proposed synchronous ultrasound imaging strategy interfaces all ultrasound patches with a shared central imaging hardware unit (fig. S1 and Fig. 1A) and then executes programmed transceiving sequences to achieve precise synchronization across the cardiac-carotid ultrasound patches. This enables the use of custom ultrasound patches, with each being independently tailored to a specific anatomical target by adjusting the center frequency, the number of array elements, and imaging mode. Image data across different physiological states could be synchronously acquired to capture dynamic physiological responses. These synchronous data are further processed by the custom AI model and human circuitry model for comorbidity evaluation.

### Wearable patch designs and characterizations

Standard acoustic windows for evaluating CHD using echocardiography commonly include parasternal long-axis (PLA), parasternal short-axis, apical, and subcostal windows, each offering distinct anatomical perspectives of the cardiac structures (fig. S2). Among them, the PLA window is frequently adopted (*31*), as it allows direct access to the cardiac structures, less interference from the lung tissue, and measurement consistency across body types. For transmission and reception through PLA, commercial cardiac ultrasound probes typically feature a compact aperture (~20 mm by 15 mm) and wide beam-steering capability (*19*, *32*, *33*), optimizing both the field of view and penetration depth. In contrast to cardiac imaging, carotid ultrasound uses a primary acoustic window on the neck (lateral to the trachea), where the superficial location of the carotid arteries enables easy accessibility (*34*, *35*). This anatomical advantage imposes less constraints to the patch design.

**Fig. 2.**
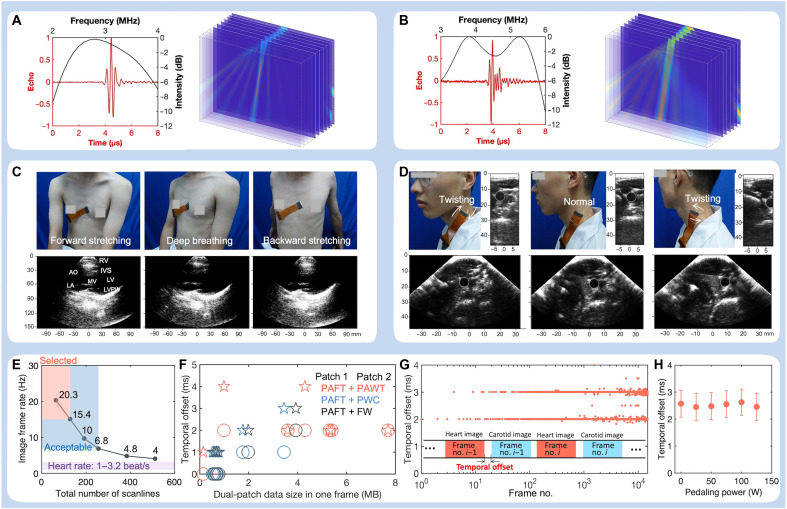
Design and characterization of the wearable ultrasound patches. (**A**) Pulse-echo characteristics (left) showing the center frequency (~3 MHz) and −6-dB bandwidth (~1.8 MHz, ~60%) of the cardiac patch; phased-array beam steering with focused transmissions for cardiac imaging (right). (**B**) Pulse-echo characteristics of the carotid patch (left), showing the center frequency of ~4.5 MHz and bandwidth of ~63% (~2.84 MHz); phased-array beam steering with wide-beam transmissions for carotid imaging (right). (**C**) Cardiac ultrasound images captured in response to various body postures, demonstrating stable image acquisition. RV, right ventricle; IVS, interventricular septum; AO, aorta; LA, left atrium; LV, left ventricle; MV, mitral valve; LVPW, left ventricular anterior wall. (**D**) Ultrasound images of the carotid artery under different postures. The plane-wave compounding imaging method (top images) fails to visualize the artery during neck twisting, whereas phased-array imaging with wide-beam transmissions (bottom images) maintains full artery visibility. (**E**) Frame rate for dual-patch imaging. (**F**) Temporal offsets for collecting cardiac-carotid image data under various combinations of imaging methods. Detailed explanation is shown in fig. S15. PAFT, phased-array imaging with focused transmissions; PAWT, phased-array imaging with wide-beam transmissions; PWC, plane-wave compounding; FW, focused-wave imaging. (**G**) Temporal offset measurements over 10,000 frames. The frame rate reaches ~18.6 Hz. (**H**) The temporal offset remained consistent across different pedaling power levels. A single-cycle burst pulse, with a center frequency defined by the patch, was transmitted for ultrasound imaging in panels (E) to (H).

The designed cardiac and carotid patches integrate diced 1-3 piezoelectric composite, dual acoustic-impedance matching layers, a flexible printed circuit (FPC), and a damping backing layer (Fig. 1, B and C; fig. S3; and Materials and Methods). We used the electrode flanging method to enable coplanar wiring of the piezoelectric composite (*36*, *37*), which enhanced signal sensitivity over the conventional wiring method (fig. S4). We also used dual matching layers to enhance the axial resolution and bandwidth (Materials and Methods and Supplementary Text). To improve beam steering, the pitch of the cardiac patch was set to 0.3 mm (~0.58 wavelength; fig. S5). We designed the carotid patch with a pitch of 0.27 mm (~0.79 wavelength). Furthermore, we bonded the piezoelectric composite to the FPC using a high-strength adhesive for robust connections (fig. S6) and selected a silicone-epoxy filler (*36*, *38*) for providing elastic compliance and acoustic isolation. The fabricated cardiac and carotid patches featured center frequencies of 3 and 4.5 MHz (Fig. 2, A and B), respectively. The patches demonstrated exceptional electrical and acoustic performances (figs. S7 and S8), exhibiting electromechanical coupling coefficients of 49% (cardiac) and 74% (carotid), bandwidths of 60 and 63%, signal-to-noise ratios (SNRs) of 25.84 ± 2.92 and 26.32 ± 1.95 dB, and cross-talk of −35 and −34 dB, respectively. The performance profile presents a clear advantage over existing ultrasound patches (table S2), such as a smaller pitch for reduced side-lobe artifacts, broader bandwidth, and a rigid design that avoids flexibility-induced imaging inaccuracies. The patches interfaced with a back-end system (Verasonics Vantage 128) via a custom connector using predefined array addresses (elements 1 to 64 for the cardiac patch and 65 to 128 for the carotid patch; fig. S9). For performing continuous imaging, the ultrasound patches were affixed to the skin using medical-grade adhesive tape (Fig. 2, C and D), enabling stable and high-quality image acquisition during movements (fig. S10).

To identify optimal imaging methods for simultaneous cardiac and carotid visualization, we evaluated three approaches for each patch: focused-wave imaging, phased-array imaging, and plane-wave compounding (fig. S11). Performance was assessed through the spatial resolution, field of view, and imaging contrast. Phantom tests showed that phased-array imaging with focused transmissions achieved reasonable imaging performance for the cardiac patch, offering a 220-mm by 155-mm field of view while maintaining acceptable imaging resolution (axial: 0.5 to 1.1 mm; lateral: 0.9 to 4.5 mm; fig. S12). In human tests, stable imaging acquisition was obtained during torso stretching and deep inspiration (Fig. 2C), with consistent high-contrast echoes across postures (fig. S13). For carotid imaging, a phased array with wide-beam transmissions delivered superior resolution (axial: 0.4 to 0.6 mm; lateral: 0.4 to 1.4 mm; fig. S14) and better contrast in phantoms, with human tests showing robustness during motion (fig. S13). After selecting the imaging methods, we improved the frame rate to ensure adequate temporal resolution for synchronous monitoring. To meet the Nyquist criteria (*39*), we implemented a total of 128 scanlines (64 per patch), achieving approximately six times the maximum HR (Fig. 2E). The proposed synchronous imaging strategy introduces a temporal offset between sequentially acquired cardiac-carotid images (figs. S1 and S15 to S17), which serves as a measure for assessing the event synchronization fidelity. Comprehensive tests across different imaging methods, parameter sets, fields of view, and frame counts demonstrate that the temporal offset is typically 0 to 4 ms, which is within 0 to 8% of the frame duration (Fig. 2, F to H, and fig. S15). Analysis showed that the temporal offset possibly introduces an error to the extracted cardiac and carotid waveforms, but the effects remain limited (fig. S16). The temporal offset also remains stable across different frame rate and physical motion levels (fig. S17). Thus, the strategy is capable of achieving synchronization suitable for diagnostic imaging purposes.

We further systematically evaluated the working performance of the dual-patch system. For both cardiac and carotid images, the imaging SNR was influenced by participant weight but remained consistent across most postures (fig. S18). Although dual-patch synchronous imaging may possibly introduce cross-talk, strong image similarity was observed between synchronous and separate imaging across different postures (figs. S19 and S20), indicating negligible cross-talk. This is likely because the cardiac and carotid imaging planes are spatially distant, and the synchronous imaging strategy was specifically designed to mitigate cross-talk by temporally switching radio-frequency acquisition between patches (fig. S1). Even without ultrasound gel, the ultrasound patch performed adequately (fig. S21); however, different body postures inevitably introduced small air gaps, degrading image quality. Therefore, ultrasound gel was used for all in vivo measurements. Under sweaty conditions, the medical tape can robustly bond the patch to the skin (fig. S21), and the wide field of view of the phased-array imaging strategy ensured continuous artery capture even during sweat-induced patch sliding. The system showed robust performance throughout extended use, with the patch maintaining image quality and user comfort during both 24-hour continuous wear and intense physical exercise, without notable degradation or adverse symptoms like skin irritation (fig. S22). Last, acoustic safety during prolonged imaging was confirmed by temperature measurement and cell viability test (fig. S23).

### Transient heart motion and pulsation

Transient heart (regional wall) motion and carotid artery pulsation under graded exercise provide important information of the cardiac-carotid status. Here, we focus on the variability, waveform characteristics, and dynamic interplay of HR, pulse rate (PR), and respiratory rate (RR), which provide valuable metrics for stratifying risk and predicting the severity of coronary and carotid artery comorbidity (Supplementary Text) (*40*, *41*). For instance, a consistently elevated resting HR with blunted postexertion recovery signals sympathetic overdrive and autonomic dysfunction, indicating underlying coronary strain. A concomitant rise in resting RR may reflect systemic circulatory inefficiency, implicating broader vascular dysfunction that could extend to the carotid arteries. Discrepancies between HR and PR can reveal arrhythmias such as atrial fibrillation, a condition linking coronary disease to elevated stroke risk in the presence of carotid pathology.

**Fig. 3.**
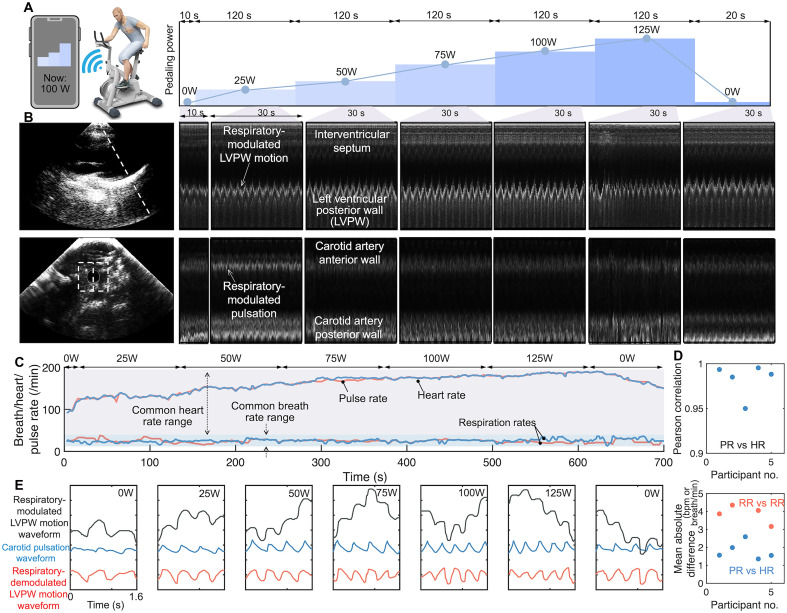
Transient heart motion and pulsation. (**A**) Pedaling power setup for collecting cardiac-carotid images under the physiological states from rest to exercise. The illustration of the pedaling figure at the left panel was created using C4D and Photoshop software. (**B**) M-mode cardiac and carotid ultrasound images under graded exercise intensity. (**C**) Extracted PR, HR, and RR by the STFT method. PRs measured from carotid M-mode ultrasound showed near-perfect agreement with HRs across all exercise intensities. (**D**) Pearson correlation coefficient and mean absolute difference between HR and PR curves for the five healthy participants (details are shown in fig. S28). The RR versus RR comparison illustrates the difference in RR when extracted via cardiac versus carotid images. The PR versus HR comparison illustrates the difference between PR and HR when extracted via carotid versus cardiac images. (**E**) Transient pulsation and heart motion waveforms show beat-to-beat motion.

To extract HR, PR, and RR, we acquired synchronized cardiac-carotid ultrasound images at progressively increasing pedaling intensities following the bicycle exercise protocol (Fig. 3A, table S1, and fig. S24). M-mode ultrasound images were further extracted from the collected B-mode image dataset, which revealed distinct motion patterns across varying exercise intensities (Fig. 3B and fig. S25). Using short-time Fourier transform (STFT) analysis (*42*), we decoupled respiratory and cardiac/pulsation motions, extracting HR, PR, and RR (fig. S26). The STFT method demonstrated accurate extraction of HR, PR, and RR across participants during both rest and exercise, as validated against commercial electrocardiography (ECG) measurements (fig. S27). Bland-Altman analysis showed reliable performance, with mean biases ranging from −0.3 to −1.2 beats per minute (bpm) for HR and 0.8 to 1.3 bpm for RR at rest and from −0.1 to −3 bpm for HR during exercise. As shown in Fig. 3C, during exercise, RR showed minor fluctuations, while HR and PR rose notably, reflecting the body’s requirement for enhanced circulatory output during physical exertion. The respiratory motion amplitude nearly doubled at high intensity, contrasting with a smaller increase in LVPW (left ventricular anterior wall) displacement (fig. S28A). HR and PR demonstrated strong concordance across participants (Fig. 3, C to E, and fig. S28B), as evidenced by the consistent temporal alignment of the values (mean absolute difference from 1.37 to 2.59 bpm), near-unity correlation coefficient (Pearson correlation ~1), and beat-to-beat waveform alignment. These findings collectively validate normal cardiovascular coupling, confirming participants’ healthy physiological status with intact peripheral pulse transmission.

### Dynamic cardiac-carotid metrics

The diagnosis of CAD by echocardiography is based on the concept that acute myocardial ischemia or infarction produces a detectable impairment in regional left ventricular (LV) mechanical function (*28*, *29*). For example (details in Supplementary Text), chronic LV remodeling, such as an increased LV internal diameter (LVID) accompanied by a reduced ejection fraction (EF), may indicate prior myocardial injury or chronic pressure overload from systemic hypertension, a major risk factor for atherosclerosis in both the coronary and carotid arteries. In addition, a reduction in stroke volume (SV) and cardiac output (CO) at rest or during stress echocardiography may reflect impaired pump efficiency. This often results from compromised coronary perfusion and may coexist with carotid artery disease resulting from shared atherosclerotic risk factors. Therefore, metrics for evaluating the LV function are important, such as LV volumetric changes, EF, and CO (*43*). For the carotid artery, metrics such as artery morphology, blood flow, and dynamic blood pressure fluctuations provide insights for stenosis analysis (Supplementary Text) (*44*). Analyzing dynamic blood pressure fluctuations within the carotid artery, such as increased pulse pressure or abnormal pressure wave reflections, can reveal heightened arterial stiffness. This stiffness offers a surrogate metric of shared pathological burden and may provide early warning of comorbidity before overt symptoms such as angina or transient ischemic attacks emerge.

**Fig. 4.**
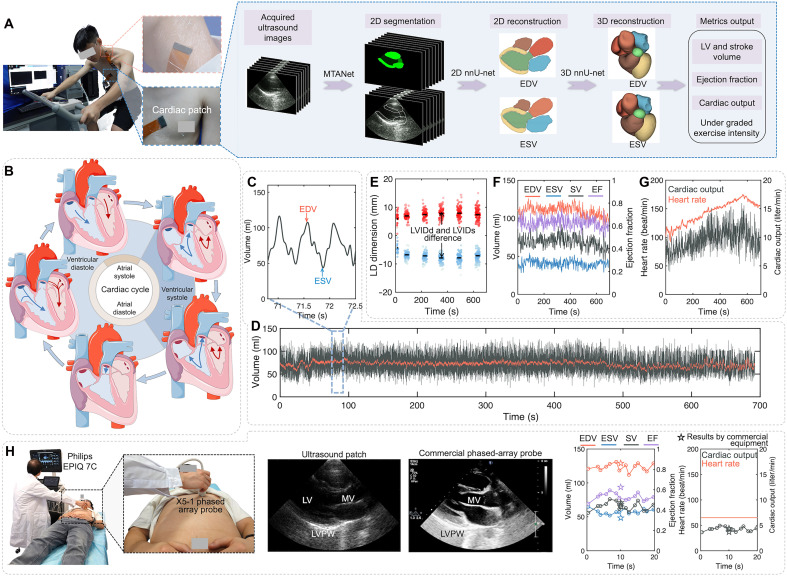
Dynamic cardiac metrics. (**A**) Workflow for quantifying cardiac metrics through automatic image segmentation and reconstruction algorithms. (**B**) The schematic of the cardiac cycle illustrates coordinated periods of relaxation (diastole) and contraction (systole) in both the atria and ventricles, working together to fill the chambers with blood and then eject it. (**C**) Detailed volumetric waveforms showing EDV and ESV. (**D**) Volumetric change of the heart. The black curve depicts cyclic volumetric variations, while the red curve shows the overall volumetric trend derived from moving average smoothing. (**E**) Exercise intensity–dependent variations in LV internal diameter at diastole (LVIDd) and LV internal diameter at systole (LVIDs). (**F**) Cardiac metrics including EDV, ESV, SV, and EF. (**G**) Transient CO and HR. (**H**) Performance validation of the cardiac ultrasound patch against commercial ultrasound equipment (Philips, EPIQ 7C); see fig. S32 for details. Ultrasound images captured by the cardiac patch (middle left panel) and commercial equipment (middle right panel) and quantitative comparison of the measured EDV/ESV/EF/CO/SV metrics.

To automatically quantify clinically important cardiac metrics, we implemented deep learning frameworks (Fig. 4A and movie S1). We developed and trained the MTANet architecture (*45*) to achieve automatic segmentation of cardiac structures from PLA views (fig. S29, Materials and Methods, and Supplementary Text). The segmentation was then completed with a two-dimensional (2D) inpainting model (using nnU-Net) and further complemented by a pretrained nnU-Net model (*46*) for 3D heart reconstruction. The reconstruction model accuracy was demonstrated under different combinations of acoustic windows, patch configurations, and motion levels of the heart (fig. S30).

The cardiac cycle consists of coordinated periods of ventricular relaxation (diastole) and contraction (systole) that facilitate chamber filling and blood ejection (Fig. 4, B and C). The cyclic ventricular volumetric response exhibited a rapid initial increase upon exercise onset, followed by a more gradual, intensity-dependent expansion (Fig. 4D). However, at high-intensity workload when the participant approached high exertion, a modest reduction in ventricular volume was observed, which was also seen in LV internal diameter at diastole (LVIDd) and LV internal diameter at systole (LVIDs) measurements (Fig. 4E). Such a response may result from the competing physiological mechanisms of increased venous return and reduced ventricular filling time. The volumetric metrics [end-diastolic volume (EDV), end-systolic volume (ESV), and SV] were further quantified from the LV volume curves (Fig. 4F), which exhibited a characteristic biphasic response to graded exercise, with an immediate increase upon exercise initiation (attributed to rapid venous return), followed by a progressive, intensity-dependent augmentation. The EF metric showed the peak of ~75% before returning to baseline levels of ~55% during the recovery phase, which illustrates the heart’s adaptive capacity to meet increased metabolic demands during physical stress, while the rapid postexercise normalization suggests preserved ventricular compliance and autonomic regulation of the participant. Furthermore, CO demonstrated a progressive increase from a resting baseline of ~5 liters/min to a peak of <~15 liters/min during maximal exertion (Fig. 4G). These hemodynamic metrics show similar trends across participants and fall within normal ranges (fig. S31). They provide clinically important measures of cardiac functions, which potentially serve as sensitive cardiovascular fitness indicators. Furthermore, the extraction method for these metrics was validated against a commercial ultrasound system under both resting and postexercise conditions (Fig. 4H and fig. S32). Measurements from the cardiac patch showed typical differences (mean ± SD) of −5.97 ± 5.29 ml for EDV, 9.28 ± 3.83 ml for ESV, −0.0956 ± 0.0385 for EF, 0.108 ± 0.443 liters/min for CO, and −2.75 ± 6.81 ml for SV. Most of these differences remained within 5% of the clinical standard, confirming the reliability of the cardiac patch measurements.

**Fig. 5.**
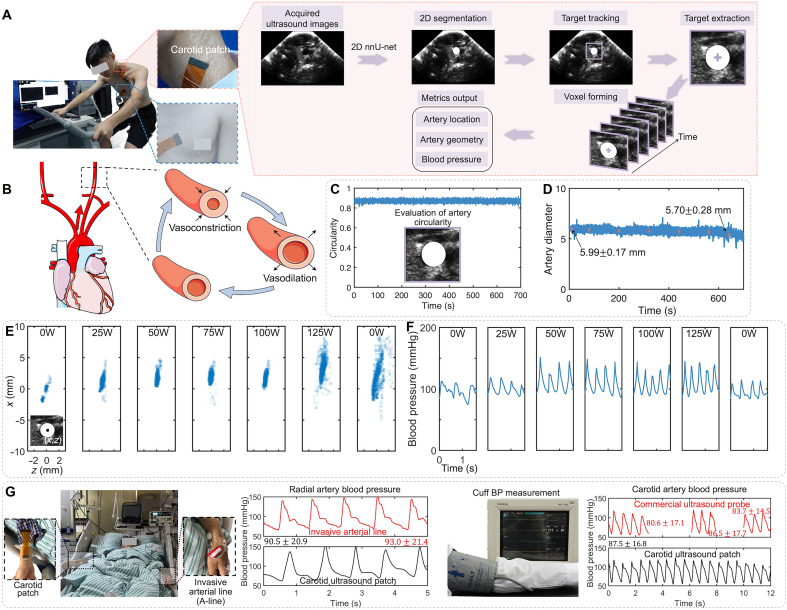
Dynamic carotid metrics. (**A**) Workflow for quantifying carotid metrics through automatic image segmentation and tracking algorithms. (**B**) Schematics of the pulsation cycle, showing vasoconstriction and vasodilation of carotid artery. (**C**) Quantitative evaluation of the arterial circularity, with the inset depicting the segmented carotid artery boundary. A circularity index of 1.0 indicates perfect geometric circularity, with decreasing values reflecting increasing deviation from an ideal circle. (**D**) Diameter variation of the carotid artery. (**E**) Exercise-induced dynamics of carotid artery geometric center displacement. (**F**) Blood pressure waveforms under graded exercise intensity. (**G**) Calibration and validation of the carotid blood pressure (BP) measurements.

Clinically important carotid parameters were also automatically extracted. A primary challenge stems from the dynamic nature of carotid artery motion during physiological activity, necessitating robust automated tracking across temporal sequences. We segmented the ultrasound images using a pretrained nnU-Net deep learning model (*47*), followed by the placement of a 15-mm by 15-mm region of interest centered on the carotid artery for subsequent analysis (Fig. 5A, fig. S33, movie S2, Materials and Methods, and Supplementary Text). The proposed tracking model enabled automatic acquisition of the artery dynamics across the entire exercise, demonstrating capabilities surpassing conventional M-mode imaging in tracking fidelity and motion characterization (fig. S34).

The pulsation cycle shows vasoconstriction and vasodilation of the carotid artery (Fig. 5B), in response to the cardiac cycle. Quantitative analysis showed consistently high arterial circularity with minimal variation (Fig. 5C). This is clinically relevant, as pathologies like atherosclerosis and stenosis typically cause irregular or noncircular lumen geometries. During exercise, the carotid artery diameter showed a slight reduction (5%) but almost doubled diameter variation (Fig. 5D). This may reflect the artery’s balancing act between constriction (from stress hormones) and enhanced pulsatility (from increased blood flow dynamics). The spatial displacement of the carotid artery center exhibited pronounced lateral movements that correlated strongly with exercise intensity (Fig. 5E), showing the participants’ increased physical motion amplitude during higher-intensity exercise. Continuous blood pressure waveforms (Fig. 5F and fig. S35) were derived from the pulsatile diameter changes of the carotid artery (*23*, *24*), serving as valuable physiological indicators for assessing individual cardiovascular status. The waveforms exhibited progressive sharpening with increasing exercise intensity, characterized by steeper systolic upslopes and reduced diastolic durations. These hemodynamic changes reflect the cardiovascular system’s adaptation to heightened metabolic demands during intense physical exertion. Furthermore, blood pressure measurements by the carotid patch have been carefully calibrated and validated against the clinical standard including the invasive arterial line and cuff method (Fig. 5G, fig. S36, and Materials and Methods).

### Evaluation of the system for coronary and carotid artery comorbidity analysis

**Fig. 6.**
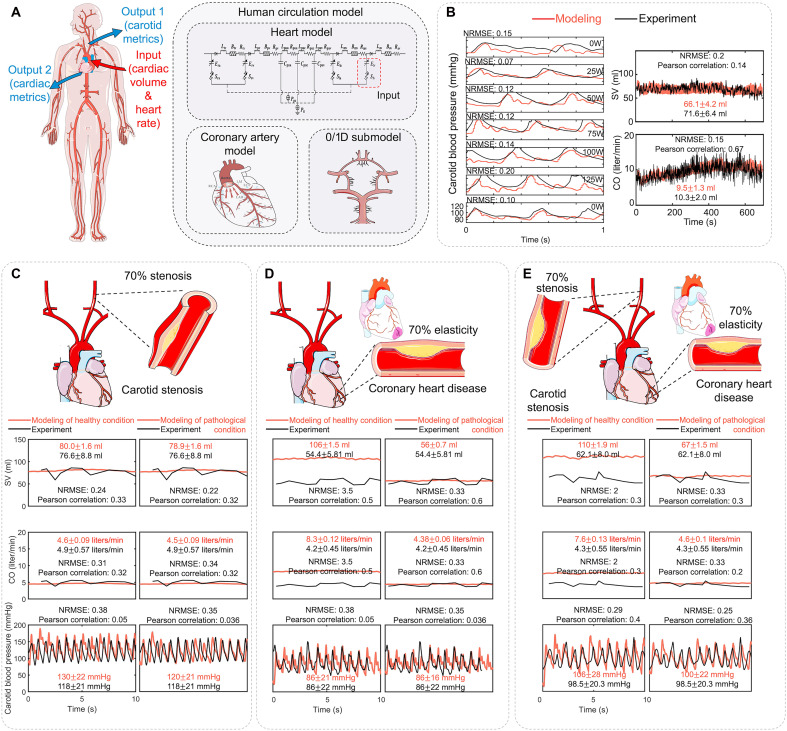
Evaluation of the system for coronary and carotid artery comorbidity analysis. (**A**) Schematic of the human circulatory model. The model uses transient cardiac volume and HR as inputs to predict carotid and cardiac metrics. (**B**) Comparison of the blood pressure waveforms, SVs, and CO between modeling and experiment. In vivo measurements obtained from patients with confirmed (**C**) CAD, (**D**) CHD, and (**E**) CAD-CHD comorbidity (see table S4 for detailed symptoms). On the basis of their clinical records, CAD was modeled as 70% arterial stenosis, and CHD was modeled by reducing myocardial compliance to 70% of the normal value (Materials and Methods). Two modeling results are shown for each patient: a healthy model [left panels of (C) to (E)] and a model incorporating the pathological condition [right panels of (C) to (E)]. Results are evaluated using the NRMSE (normalized root mean square error), Pearson correlation, mean, and standard deviation. The anatomical illustrations in (A) and top panels of (C) to (E) were referenced from Servier Medical Art, licensed under CC BY 4.0 (https://creativecommons.org/licenses/by/4.0/deed.en).

The derived cardiac-carotid metrics could be evaluated by experienced clinicians for comorbidity analysis. However, dynamic metrics are relatively scarce, as conventional methods mainly rely on static metrics, which makes evaluation difficult. In this work, to evaluate the performance of the developed system for comorbidity analysis, we further developed a human circulatory model that couples the heart and carotid hemodynamically (Fig. 6A, fig. S37, table S3, and Materials and Methods). The model integrates time-varying LV volume and HR measurements (Figs. 3C and 4D) as inputs, simulating dynamic changes in key cardiac-carotid metrics such as SV, CO, blood pressure, blood flow, and vessel diameter. For healthy participants, the model demonstrates good agreement with experimental data for blood pressure waveforms, SV, and CO (Fig. 5B). To evaluate metric variation under pathological conditions, we introduced CAD and CHD scenarios into the model (figs. S38 to S40 and Materials and Methods). Modeling with 50% carotid artery stenosis (representing mild to moderate blockage and an early pathological stage) resulted in a slight average blood pressure deviation of −4% from the healthy baseline. With 70% stenosis (severe blockage), the deviation increased to −8%. Furthermore, the progression of CHD often leads to reduced myocardial compliance. By introducing a 10% reduction in compliance (an early stage of CHD) and a 20% reduction (moderate stage), results showed −16 to −33% changes in both SV and CO from the healthy baseline.

To further validate the system performance in vivo, we recruited three patients with confirmed conditions of CAD, CHD, or CAD-CHD comorbidity (Fig. 6, C to E, and table S4). For each patient, we generated two modeling results: one simulating a healthy condition (contrary to the patient’s actual diagnosis) and another modeling the pathological state of the patient on the basis of the clinical records. As shown in Fig. 6C, carotid stenosis had limited impact on CO and SV but differs from the pathological modeling results with an average raised blood pressure of ~10 mmHg. In the patient with CHD (Fig. 6D), SV and CO deviated from the healthy model and closely matched pathological modeling results. For the CAD-CHD comorbidity case (Fig. 6E), all metrics showed deviation from the healthy model and good correspondence with pathological modeling. Collectively, these findings showed the effectiveness of the system in yielding measurements in good correlation to pathology-based simulations.

## DISCUSSION

The comorbidity of coronary and carotid artery disease is common, posing challenges for clinical management. Unfortunately, current diagnostics are typically initiated only after symptoms appear, missing the window for early intervention. Furthermore, as these conditions are usually examined separately with different modalities, no single standard test exists for their simultaneous diagnosis. To bridge this gap, we propose a wearable ultrasound strategy that integrates customizable ultrasound patches, a synchronous imaging strategy, AI-powered image processing algorithms, and cardiac-carotid hemodynamic models to enable the continuous, synchronous extraction of clinically important cardiac-carotid metrics for diagnostic support. Throughout this framework, errors originating from individual components may propagate through subsequent steps, where they can be either amplified or attenuated. This propagation issue was systematically investigated (fig. S41). Among the identified error sources, the combined uncertainty from image acquisition, AI-based segmentation and reconstruction, and metric extraction (i.e., the errors introduced in patch-derived cardiac metrics, up to 5 to 15%) might contribute mostly to the overall uncertainty in comparing metrics derived from modeling versus measurements. A secondary but notable contribution arises from a model-based inference error, which accounts for roughly 10% of the uncertainty. Regardless of these, in vivo patient measurements using the proposed system demonstrate its feasibility for distinguishing among healthy participants and patients with CAD, CHD, or CAD-CHD comorbidity. Accordingly, we position the proposed method as a diagnostic support tool intended to suggest the possible presence or early signs of comorbidity. The framework is not designed to replace comprehensive clinical evaluation but rather to serve as an adjunct that highlights potential early comorbid patterns warranting further investigation. In contrast to conventional diagnosis, which typically relies on separate, site-by-site examinations initiated only after symptom onset, our method potentially offers a more efficient pathway for early intervention in comorbidity assessment and treatment (fig. S42).

Several challenges remain for further improvements. First, this study recruited only a small group of healthy participants and patients, and future work would include more participants with a variation in age, sex, body mass index, and CAD or CHD conditions to evaluate the strategy’s clinical effectiveness. Second, the generalization of the developed AI models requires improvement through training on larger, more diverse datasets. Now, only cardiac and carotid ultrasound images are displayed in real time. Synchronous data are processed offline. While real-time AI processing would be feasible, it requires hardware acceleration, optimized algorithms, and seamless system integration to function within the stringent latency constraints of a live clinical examination. This represents a primary direction for our future development. Third, the current circulatory model relies on generalized hemodynamic parameters and therefore lacks patient specificity. To address this limitation, we conducted a sensitivity analysis of the modeling parameters (fig. S39) alongside a non-Newtonian flow analysis (fig. S40), both of which facilitate model adjustment to better fit individual patient states. Furthermore, given that the comorbidity assessment relies on model fitting with assumed pathological parameters, we analyzed the identifiability of inverse mapping from measured metrics to disease states (fig. S43). Results indicate that although most simulation parameter pairs remain identifiable, diagnostic ambiguity persists, primarily between LV elastance and end-systolic elastance parameters. Other metrics extractable by our method, such as waveform features, HR, PR, RR, and vessel dynamics, may help resolve these ambiguities. In addition, while blood pressure appears to have limited sensitivity to CAD, integrating blood flow metrics into the proposed framework could possibly enable better evaluation. Nevertheless, in-depth analysis remains a critical direction for future work. Last, the Verasonics system used in this study is limited for continuous and wearable monitoring, as it is not portable and relies on wired connections despite the fact that notable studies have used Verasonics systems for image acquisition (*19*, *21*). To enable daily monitoring for early diagnosis, the system would need to be miniaturized into autonomous wearable units with embedded processing capabilities. Integrated wearable ultrasound devices remain limited (*18*), and integrated systems for B-mode imaging face challenges in power consumption and battery life, which have yet to be addressed through advances in electronics. Furthermore, our application would require reliable interunit communication for synchronous imaging, such as using the master-slave transceiver method. Addressing these challenges is essential for practical implementation. The implications of the proposed strategy may extend beyond synchronous cardiac-carotid monitoring for comorbidity evaluations, for example, investigating the physiological interactions across anatomical systems by simultaneously tracking cardiac-pulmonary, renal-hepatic, or neurovascular systems.

## MATERIALS AND METHODS

### Design and fabrication of the wearable ultrasound patches

The ultrasound patches were designed as phased arrays to provide standardized imaging planes for both cardiac (PLA) and carotid (transverse) views. The detailed fabrication process is illustrated in fig. S3. We selected the 1-3 piezoelectric composite for transceiving ultrasound, given that it has high electromechanical coupling and low acoustic impedance, which improves energy transmission to the human tissue. Different from typical ultrasound patches that do not have matching layers (*18*, *19*, *21*, *23*, *24*), we improved the array design by the electrode flanging method such that two matching layers can be added to the front side of the piezoelectric composite. Such a configuration enables to improve the echo signal amplitude (fig. S4). After bonding the array to the customized FPC, we designed the high-damping backing layer (~6 MRayls) attached to the backside of FPC to increase the bandwidth and further improve the imaging resolution.

For conformable ultrasound patches that adapt to curved skin surfaces, uncertainties in the array’s spatial position can introduce notable errors in transmit-receive delays and beamforming (*48*, *49*). These inaccuracies would distort ultrasound images and degrade imaging precision. To address this challenge, we developed an improved fabrication method for the ultrasound patch, enhancing its ability to maintain consistent array positioning on curved skin surfaces and thereby improving beamforming accuracy. This is achieved by preserving a thin, continuous layer in the top second matching layer, instead of fully dicing through the matching-composite structure in the thickness direction. This undiced matching layer maintains the transducer elements in a flat plane during conformal skin attachment, mitigating curvature-induced beamforming errors (Fig. 1B and fig. S3). To ensure optimal acoustic coupling and secure patch attachment, we applied ultrasound gel between the patch and the skin surface and then fixed the assembly in place using medical-graded adhesive tapes (3M Tegaderm films). This approach maintained consistent transducer-skin contact while allowing natural tissue motion during movement (Fig. 2, C and D).

During encapsulation, the device was secured in a petri dish while customized filling material (silicone-epoxy composite) was injected into the array kerfs. To protect the top matching layer from contamination, we masked it with polyimide tape before filling, preventing deposition of high-damping material that could degrade acoustic performance. The device was placed in an oven for curing at 45°C and 1 hour. Last, the silicone elastomer (Ecoflex) was further poured into a mold and cured to finish the encapsulation of the device.

### Characterization of the patches

The pulse-echo responses of the ultrasound patches were characterized using a commercial pulser-receiver (DPR300, JSR Ultrasonics, US). The received radio-frequency signals were processed via fast Fourier transform spectral analysis to determine the center frequency and −6-dB bandwidth. The SNR of the patch was measured by analyzing echo signals with and without a reflector (fig. S7). Patch cross-talk was characterized by comparing the signal amplitude of an adjacent element with the input signal amplitude (fig. S8). To determine the optimal imaging methods for visualizing the carotid and heart, we conducted a comparative evaluation of four approaches: (i) plane-wave compounding, (ii) focused-wave imaging, (iii) phased-array imaging with focused transmissions, and (iv) phased-array imaging with wide-beam transmissions. Each method was assessed across three key metrics: spatial resolution, imaging contrast, and field of view. Spatial resolution was calculated on the basis of the full width at half maximum method (*36*–*38*); imaging contrast was quantitatively assessed through pixel intensity differentials, where a high brightness ratio between the target region (e.g., carotid artery lumen) and the surrounding background tissue indicates superior imaging contrast. Measurements were first conducted on a general-purpose ultrasound phantom (CIRS model 054GS) to measure the metrics in a standard environment. The phantom contains 100-μm-diameter wires (appearing as scatters in ultrasound images) and 8-mm-diameter cylinders (cyst-like structures) placed at different positions for mimicking the human body environment. Temperature measurement and cell viability test (fig. S23) were conducted for validating the acoustic safety during prolonged imaging. To assess in vivo imaging performance, ultrasound images were acquired across various cardiac-carotid postures (figs. S18 to S20), acoustic coupling conditions (fig. S21), and prolonged conditions (fig. S22).

### Carotid blood pressure calibration and measurements

Carotid blood pressure was derived from B-mode ultrasound images captured by the carotid patch. To analyze arterial pulsation, the moving artery was segmented using a nnU-Net model (Fig. 5A), and the artery diameter was then extracted and used to calculate the carotid blood pressure waveform by

$$p\left(t\right)=p_{d}e^{\alpha \left(\frac{A\left(t\right)}{A_{d}}-1\right)}$$ (1)

where $p_{d}$ is the diastolic blood pressure, α is the stiffness coefficient of the artery, A(t) is the transient carotid arterial cross section, and $A_{d}$ is the diastolic arterial cross section. The carotid patch can measure A(t) and $A_{d}$ but requires prior calibration of diastolic pressure $p_{d}$ and the stiffness coefficient α to derive blood pressure. The coefficient α can be obtained by

$$\alpha =\frac{A_{d}\text{ln}\left(p_{s}/p_{d}\right)}{A_{s}-A_{d}}$$ (2)

where $p_{s}$ is the systolic blood pressure, and $A_{s}$ is the systolic arterial cross section. Calibration of α, $p_{s}$, and $p_{d}$ follows the procedure illustrated in fig. S36. First, systolic and diastolic blood pressures ($p_{s}$ and $p_{d}$, respectively) are measured via a standard method, such as an invasive arterial line (which provides direct waveform data) or a cuff. Simultaneously, ultrasound is used to measure the transient cross-sectional diameter of the artery, from which the systolic and diastolic cross-sectional areas ($A_{s}$ and $A_{d}$, respectively) are calculated. Commonly, these parameters ($p_{s}$, $p_{d}$, $A_{s}$, and $A_{d}$) are measured at the radial or brachial artery. Second, α is calculated on the basis of Eq. 2. Notably, for a single subject, the parameters α and $p_{d}$ do not change notably along the arterial tree. Therefore, a calibration performed at the radial artery can be used to estimate blood pressure waveforms at other locations, such as the carotid artery. However, α varies between individuals and must be recalibrated when the subject’s physiological state changes (e.g., before and after exercise). In this work, blood pressure was calibrated using both the invasive arterial line and cuff method (fig. S36). The results from both methods confirmed the reliable performance of the developed patch. Furthermore, calibrations were conducted across different postures and pedaling power levels (fig. S36), enabling robust measurement of carotid blood pressure under varying physiological conditions.

### Ultrasound image processing algorithms

The cardiac ultrasound images were first segmented on the basis of the proposed MTANet framework (fig. S29; see Supplementary Text for details). MTANet (*45*) incorporates a reverse addition attention module and a parallel partial decoder within the basic UNet’s decoder to improve high-resolution feature extraction for the segmentation branch. It also uses attention bottleneck modules in the fully connected layers to integrate imaging features with clinical features for the classification branch. We trained the cardiac MTANet model for the PLA view. Training data were sourced from the public EchoNet-LVH dataset (12,000 videos; https://echonet.github.io/lvh/) and a self-established dataset (78 frames from 59 cases; https://doi.org/10.5281/zenodo.19470593). A total of 6448 frames were annotated by an experienced radiologist and reviewed by a senior radiologist, with separate annotation feature extraction and standardization performed for seven cardiac structures. For each cardiac structure, the public dataset served as the training set and the self-established dataset as the test set. Training used a comprehensive augmentation scheme (color adjustments, random rotations, and flips), with images resized to 352 by 352 pixels. The model was trained for 185 epochs using an AdamW optimizer (initial learning rate, 1 × 10^−5^; weight decay, 1 × 10^−4^) with a step-decay scheduler and a batch size of 16. A composite loss function combining weighted binary cross-entropy and weighted Intersection over Union was used for segmentation, while classification was treated as a regression problem with a mean squared error and L1 losses. The model achieved an overall average Dice coefficient of 0.843 ± 0.029 on the test set, demonstrating consistent cross-institutional performance. Given the current lack of large annotated datasets for patch-based ultrasound, this segmentation pipeline represents a balanced and feasible compromise to avoid overfitting on limited data. The segmented outputs were then processed using a 2D inpainting model based on nnU-Net, followed by 3D cardiac image reconstruction using a pretrained nnU-Net model (*46*). The reconstruction performance was systematically evaluated across different patch designs (linear or orthogonal), acoustic windows, and the effect of cardiac motion (Supplementary Text).

For analyzing the carotid ultrasound images, given the scarcity of high-quality manually annotated time-series data at the initial study stage, we adopted a targeted training strategy to achieve precise automated segmentation of the carotid artery under limited data conditions (fig. S33 and Supplementary Text). For each healthy participant, 50 representative images were randomly selected to construct a dedicated training subset, meticulously annotated by experienced imaging specialists. The model was then iteratively trained on this subset until it reached an overfitted state, enabling it to learn the morphological features, grayscale distribution, and boundary information of the target region. This overfitted model was subsequently applied to the remaining unannotated frames of the same case to automate full time-series segmentation. While this phased approach prioritizes segmentation accuracy over generalization to address initial data constraints, future work will focus on constructing a comprehensive multicenter, multisample dataset and using standardized machine learning techniques, including *K*-fold cross-validation, data augmentation, and regularization, to systematically enhance model generalizability across varying data distributions, individual differences, and imaging conditions, thereby laying a solid foundation for broader clinical implementation. The models were implemented using Python 3.10 on a computer equipped with an Intel Core i9-13900K central processing unit and an NVIDIA GeForce RTX 4090 graphics processing unit.

### Establishment of the human circulatory model

We developed a human circulatory model to simulate the synchronized cardiac-carotid metrics under pathological conditions. The model integrates simplified 0D and 1D representations of the circulatory system (fig. S37), where the 1D model simulates arterial blood flow and wave propagation, with governing equations derived via dimensional reduction of the 3D Navier-Stokes equations, and the 0D lumped-parameter model (obtained through linearization of 1D equations) captures flow dynamics in the heart, capillaries, and veins. Coupled together, these components enable full circulatory system simulation. Our early study has demonstrated the effectiveness of the modeling method for investigating the transmural myocardial flow (*50*). Using experimentally measured time-varying LV volume and HR as inputs, the model outputs dynamic hemodynamic parameters, such as blood pressure, carotid artery diameter, SVs, COs.

The 1D model of the main branches of the arterial system (including coronary circulation) and a 0D model of the peripheral circulation were constructed on the basis of population-averaged anatomical data of the human vascular system reported in our previous work (*51*). Recent studies have demonstrated that 0/1D modeling matches the diagnostic accuracy of standard clinical methods for assessing patient stenosis (*52*). The main parameters of the 1D model include the length of each artery and the proximal/distal radius of each blood vessels, and the main parameters of the 0D model include the reference total impedance and the compliance of intramyocardial blood vessels at the distal end of the coronary artery. The 1D governing equation for arterial blood flow is the classic conservation of mass and momentum equations, which is obtained by integrating the 3D Navier-Stokes equations on the vascular cross section on the basis of specific assumptions about arterial blood flow (such as assuming that blood pressure is uniformly distributed on the vascular cross section, and the blood flow velocity is dominated by axial components, while radial components can be ignored)

$$\frac{\partial A}{\partial t}+\frac{\partial Q}{\partial z}=0$$ (3)

$$\frac{\partial Q}{\partial t}+\frac{\partial }{\partial z}\left(\gamma \frac{Q^{2}}{A}\right)+\frac{A}{\rho }\frac{\partial P}{\partial z}+F_{r}\frac{Q}{A}=0$$ (4)

where t represents the time, z represents the axial coordinates of blood vessels, ρ represents the blood density (= 1.06 g/cm^3^), A represents the cross-sectional area of blood vessels, Q is the volumetric flow rate, P is the blood pressure, γ is the momentum flux correction coefficient (herein set to 4/3), and $F_{r}$ represents the frictional force per unit length of blood vessels. The blood flow velocity profile is based on Poisson’s law and according to the general consensus that the blood flow in coronary arteries is close to Newtonian fluid.

As illustrated in fig. S37, the cardiac model is positioned upstream of the 1D arterial model, serving as the driving force for the entire circulatory system. In this study, we used a simplified elastic chamber model to represent the cardiac function, where blood flows from the left ventricle into the 1D arterial network. The cardiac model features time-dependent periodic variations in chamber pressure to simulate dynamic cardiac contraction-relaxation cycles. The blood pressure in each cardiac chamber is given by

$$P\left(t\right)=E\left(t\right)\left(V-V_{0}\right)+S\frac{\mathrm{dV}}{\mathrm{dt}}$$ (5)

Herein, V is the volume of each chamber of the heart, $V_{0}$ is the unstressed volume, and *S* is the viscoelastic coefficient of the heart wall, which is linearly related to heart pressure (*53*). E(t) is a time-varying elasticity (*54*)

$$E\left(t\right)=E_{\text{sva}}e\left(t\right)+E_{\text{svp}}$$ (6)

Herein, the two terms on the right side of the formula are the time-varying chamber elasticity caused by myocardial disease and the baseline stiffness of the chamber (passive elasticity) without active stimulation. In the four-chamber model, *E*_sva_ and *E*_svp_ of the left ventricle, left atrium, right ventricle, and right atrium were represented by *E*_lva_ and *E*_lvp_, *E*_laa_ and *E*_lap_, *E*_rva_ and *E*_rvp_, and *E*_rasa_ and *E*_rasp_, respectively. *E*(*t*) is a normalized time-varying function of active elasticity, expressed as a piecewise function. It should be noted that the piecewise functions of the ventricle (*e*_ventricle_) and atrium (*e*_atrium_) are represented in different forms.

For the left and right ventricles, the piecewise functions were expressed as

$$e_{\text{ventricle}}=\left\{ \begin{array}{c} 0.5\left[1-\text{cos}\left(\pi t/T_{\text{vcp}}\right)\right],0\leq t\leq T_{\text{vcp}}\\ 0.5\left\{1+\text{cos}\left[\pi \left(t-T_{\text{vcp}}\right)/T_{\text{vcp}}\right]\right\},T_{\text{vcp}}<t\leq T_{\text{vcp}}+T_{\text{vrp}}\\ 0,T_{\text{vcp}}+T_{\text{vrp}}<t\leq T_{0} \end{array}\right.$$ (7)

and for the left and right atrium, the function is expressed as

$$e_{\text{atrium}}=\left\{ \begin{array}{c} 0.5\left\{1+\text{cos}\left[\pi \left(t+T_{0}-t_{\text{ar}}\right)/T_{\text{arp}}\right]\right\},0\leq t\leq t_{\text{ar}}+T_{\text{arp}}-T_{0}\\ 0,t_{\text{ar}}+T_{\text{arp}}-T_{0}<t\leq t_{\text{ac}}\\ 0.5\left\{1-\text{cos}\left[\pi \left(t-t_{\text{ac}}\right)/T_{\text{acp}}\right]\right\},t_{\text{ac}}<t\leq t_{\text{ac}}+T_{\text{acp}}\\ 0.5\left\{1+\text{cos}\left[\pi \left(t-t_{\text{ar}}\right)/T_{\text{arp}}\right]\right\},t_{\text{ac}}+T_{\text{acp}}<t\leq T_{0} \end{array}\right.$$ (8)

Under actual physiological conditions, the left and right ventricles are an organic unity rather than working alone. To characterize the mechanical interaction between the left and right ventricles through the interventricular septum, we introduced a mathematical model consisting three elements (namely, $E_{\text{lv}}$, $E_{\text{rv}}$, and $E_{s}$) to characterize them (*53*)

$$\begin{array}{c} p_{\text{lv}}=E_{s}\frac{E_{\text{lv}}}{E_{s}+E_{\text{lv}}}V_{\text{lv}}+\frac{E_{\text{lv}}}{E_{s}+E_{\text{lv}}}p_{\text{rv}}\\ p_{\text{rv}}=E_{s}\frac{E_{\text{rv}}}{E_{s}+E_{\text{rv}}}V_{\text{rv}}+\frac{E_{\text{rv}}}{E_{s}+E_{\text{rv}}}p_{\text{lv}} \end{array}$$ (9)

where $E_{s}$ represents the elastic compartment due to the shift of the septum, $p_{\text{lv}}$ represents the left ventricle pressure, and $p_{\text{rv}}$ represents the right ventricle pressure.

Pathological changes are modeled by varying the corresponding parameters, as described below. In this study, CAD was modeled by introducing stenosis into the common carotid artery at two severity levels: 50%, representing a clinically significant threshold where flow reduction becomes hemodynamically relevant (i.e., a relatively early stage of carotid artery disease), and 70%, representing a severe stage. CHD was modeled by reducing the myocardial compliance parameters ($E_{\text{lva}}$ and $E_{\text{lvb}}$) by 10 and 20% from their baseline values. A 10% reduction corresponds to an early stage of CHD, while a 20% reduction represents a more advanced, moderate stage.

The 1D governing equations offer limited capability in predicting stenosis-related pressure gradients. For accurate representation, we supplemented it with a data-fitting stenosis model (*55*) that maps geometric parameters of arterial narrowing to their hemodynamic consequences

$$\Delta P=\frac{K_{v}\mu }{A_{0}D_{0}}Q+\frac{K_{t}\rho }{2A_{0}^{2}}{\left(\frac{A_{0}}{A_{s}}-1\right)}^{2}Q|Q|+\frac{K_{u}\rho L_{s}}{A_{0}}\dot{Q}$$ (10)

The hemodynamic variables include the following: Δ*P* (pressure differential across the stenosis) and *Q* (volumetric flow rate, with $\dot{Q}$ representing its temporal derivative). Geometric parameters comprise *A*_0_ (unconstrained cross-sectional area), *A*_s_ (minimum stenotic area), *L*_s_ (axial length of stenosis), and μ (dynamic blood viscosity). The empirical coefficients are specified as follows: *K*_v_ = 32(0.83*L*_s_ + 1.64*D*_s_)(*A*_0_/*A*_s_)^2^/*D*_0_, *K*_t_ = 1.52, and *K*_u_ = 1.2, where *D*_0_ and *D*_s_ represent the diameters corresponding to *A*_0_ and *A*_s_, respectively. Stenosis severity is quantified as the percentage diameter reduction: (1 − *D*_s_/*D*_0_) × 100%. Detailed simulation parameters are listed in table S3.

### System setup and data collection

With ethical approval from Jinhua Municipal Central Hospital (ethic approval no. 2025-Ethics Review-225), we recruited two participant groups for in vivo measurements (table S4). We obtained informed consent for the measurements. The first group comprised five healthy participants who completed a graded bicycle exercise protocol. Exercise intensity was increased from rest (0 W) with an increment of 25 W every 2 min, and all participants in this group discontinued the test at 125 W because of fatigue. The second group included three patients with confirmed CAD, CHD, or CAD-CHD comorbidity, who were assessed at rest for safety considerations. All participants underwent clinical-standard screening at Jinhua Municipal Central Hospital (a grade three class A hospital) to confirm their suitability and safety for attending our test. Our measurement system mainly features a programmable multichannel back-end controller (Verasonics Vantage 128 system), two front-end patches, and electronically controlled resistance spin bikes with precision workload calibration. One hundred twenty-eight channels of the back-end controller were equally allocated (64 channels per patch) via a customized cable and printed circuit boards (fig. S9). Ultrasound transceiving sequences, data acquisition, and storage are all programmed and controlled through MATLAB. MATLAB generates control commands that orchestrate the back-end hardware to drive both ultrasound patches with programmed imaging sequences. As illustrated in fig. S1, a customized acquisition program was developed, which enables synchronous carotid-cardiac imaging through alternating activation of each patch. The system sequentially calls the cardiac and carotid patch for data acquisition and then saves both image datasets. This acquisition loop continues until manual termination, achieving functionally simultaneous monitoring of both the carotid and heart. Results from one healthy participant are presented in the main text (Figs. 3 to 5), while those from additional healthy participants are provided in the Supplementary Materials. Patient testing results are reported in Fig. 6.

## Supplementary Materials

### The PDF file includes:

Supplementary Text Figs. S1 to S43 Tables S1 to S4 Legends for movies S1 and S2 References
