# Supplementary material for: Synchronous wearable ultrasound for early detection of coronary and carotid artery comorbidity
Source: Sci Adv. 2026 Jun 19;12(25):eaed2114. doi: 10.1126/sciadv.aed2114 (PMC13281808; doi:10.1126/sciadv.aed2114)
Supplement: Supplementary file 1 — Supplementary Text Figs. S1 to S43 Tables S1 to S4 Legends for movies S1 and S2 References [file sciadv.aed2114_sm.pdf]

Science Advances
AAAS

Supplementary Materials for

Synchronous wearable ultrasound for early detection of coronary and carotid artery comorbidity

Shengrong Lin *et al.*

Corresponding author: Kang Chen, kangchen@zjnu.edu.cn; Jianming Wen, wjming@zjnu.cn; Dexing Kong, dkong@zju.edu.cn

Sci. Adv. **12**, eaed2114 (2026)
DOI: 10.1126/sciadv.aed2114

The PDF file includes:

Supplementary Text
Figs. S1 to S43
Tables S1 to S4
Legends for movies S1 and S2
References

Other Supplementary Material for this manuscript includes the following:

Movies S1 and S2

Supplementary Text

Traditional diagnosis methods for concomitant coronary and carotid artery disease

Since concomitant coronary and carotid artery disease involves both heart and carotid, its screening requires examination of both sites.

The severity and extent of coronary artery stenosis correlate with the urgency for carotid artery evaluation (*56*). Patients with a confirmed diagnosis of coronary heart disease may undergo carotid artery auscultation. If clear positive findings are detected on carotid auscultation, carotid ultrasound should be performed as the initial non-invasive imaging test. If ultrasound confirms significant stenosis, further evaluation with carotid angiography may be necessary if intervention is being considered.

For patients with a confirmed diagnosis of carotid artery stenosis, a clinical history may be taken to assess for coronary heart disease, and an electrocardiogram (ECG) may be performed[7]. If coronary heart disease is suspected and no contraindications exist, exercise stress ECG and coronary computed tomography angiography (CTA) are recommended (*57*).

Traditional clinical methods and measurement metrics are listed in the following.

- CAD examination: 1) Doppler ultrasound is a first-line examination method that provides key diagnostic metrics, including peak systolic velocity (PSV) and end-diastolic velocity. For clinical evaluation, a PSV >230 cm/s suggests severe (≥70%) stenosis, a key threshold for intervention (*58*). 2) CTA is a method for precise evaluation, offering detailed anatomical visualization to confirm ultrasound findings and assist in pre-procedural planning (*59*). Key diagnostic metrics include percentage diameter stenosis and plaque ulceration.
- CHD examination: 1) Exercise stress ECG can indicate exercise-induced myocardial ischemia by assessing ST-segment depression (*60*). 2) Coronary CTA anatomically identifies obstructive plaque and provides an overview of overall atherosclerotic burden via the calcium score (Agatston score). Key diagnostic metrics include percentage diameter stenosis and the calcium score (*61*). 3) Invasive angiography with fractional flow reserve (FFR) is the gold standard evaluation method. It provides percentage diameter stenosis and FFR, with an FFR ≤0.80 identifying specific lesions that cause ischemia and require stenting (*62*).

Advantages for adopting the stress echocardiography protocol

The selected stress echocardiography protocol involves the simultaneous capture of cardiac-carotid dynamics under graded exercise intensities, which is superior to static assessments as it moves beyond mere anatomical measurement to capture the integrated physiological response of the cardiovascular system under stress. Atherosclerosis is a systemic disease, and a protocol that challenges both the heart and the carotid arteries simultaneously can reveal vulnerabilities that remain hidden during rest. By using physiological stress as a trigger, this method provides a dynamic window into the functional health of two critical vascular beds, potentially allowing for the identification of co-morbidity before symptoms or catastrophic events like heart attacks or strokes occur.

A primary advantage is its unique capacity for the simultaneous and provocative assessment of both organ systems. The stress echocardiogram by the wearable patch evaluates coronary artery disease by unmasking regional wall motion abnormalities in the heart when myocardial oxygen demand exceeds supply. Concurrently, high-resolution ultrasound patch can measure the functional response of the carotid artery under the same stress, assessing metrics such as vessel

distensibility and flow-mediated dilation. This dual assessment means that a single, coordinated test can identify hemodynamically significant disease in both the coronary and carotid circulations that might not be apparent under resting conditions, providing a comprehensive picture of systemic atherosclerotic burden.

Furthermore, this methodology might be effective for detecting unmasking subclinical disease. Many individuals possess atherosclerotic plaques that do not cause significant narrowing or symptoms at rest. However, the increased cardiac output, heart rate, and blood pressure generated during stress act as a valuable physiological challenge. A coronary lesion may only impede flow during exertion, just as a carotid plaque may only demonstrate reduced vascular compliance or create turbulent flow when subjected to higher shear stress. The stress echo protocol may effectively provoke these hidden vulnerabilities, enabling clinicians to identify at-risk patients possibly before the development of irreversible damage or clinical events.

The protocol also offers practical advantages, being both safe and highly informative for risk stratification. Unlike other imaging modalities such as CTA tests, stress echocardiography utilizes ultrasound and involves no exposure to ionizing radiation, which makes it an ideal tool for continuous monitoring. The collected dynamic data, such as exercise capacity, blood pressure response, and the dynamic interaction between cardiac and carotid function, would provide an unprecedented risk profile that goes beyond traditional static factors. This allows for more personalized and aggressive preventive strategies in patients who demonstrate poor cardiovascular reserve during testing.

In conclusion, the stress echocardiography protocol was employed in this study for dynamic evaluation of the cardiac-carotid status. It recognizes the interconnectedness of the cardiovascular system and leverages a controlled stressor to potentially reveal early-stage co-morbidity that static scans cannot detect. By providing a simultaneous assessment of cardiac and carotid vulnerability within a safe and widely available framework, this protocol would stand as a valuable tool for pre-emptive medicine, possibly facilitating intervention to mitigate the significant risks associated with advanced coronary and carotid artery disease.

Design of two matching layers

The significant acoustic impedance mismatch between human skin (~1.5 MRayl) and piezoelectric materials (20-30 MRayl) causes substantial ultrasound energy reflection at the interface. Multilayer matching systems can effectively bridge this impedance transition, with optimal designs achieving >90% energy transmission. In this work, we adopt the dual-matching layer design scheme. Based on the KLM transmission theory (*63*), employing two quarter-wavelength thick matching layers enables efficient ultrasound transmission. The acoustic impedance of two matching layers can be calculated by

$$Z_1 = (Z_p^4 \cdot Z_t^3)^{1/7} \tag{S1}$$

$$Z_2 = (Z_p^1 \cdot Z_t^6)^{1/7} \tag{S2}$$

where Z_p and Z_t represent the acoustic impedance of the piezoelectric material and tissue, respectively. In this study, Z_p and Z_t are 12.8 and 1.5 MRayls, respectively.

For both cardiac and carotid patches, the first matching layer was made of a mixture of carbon graphite and Epo-Tek 301 (Epoxy Technology Inc, Billerica, MA, USA) which measures around

5.5 MRayls in acoustic impedance, and the second matching layer was made of pure Epo-Tek 301 (~2.5 MRayls). The difference of matching layers between two patches lies in the thickness: for the cardiac patch, the thickness of two matching layers are around 0.23 mm and 0.2 mm, and those for the carotid patch are 0.14 mm and 0.12 mm.

Significance of evaluating transient pulsation and cardiac motion

M-mode ultrasound images can be extracted from the collected cardiac-carotid B-mode images, which enables the analysis of heart rate (HR), pulse rate (PR), and respiratory rate (RR). Their variability, waveform characteristics, and dynamic interplay provide valuable metrics for evaluating the coronary and carotid artery co-morbidity.

HR and PR: In a healthy person at rest, HR and PR are identical. However, in certain arrhythmias, the pulse rate can be lower than the heart rate (a pulse deficit). For early diagnosis, we look beyond the absolute number to its variability and response to stress: (1) A consistently high resting HR (>80 bpm) is a marker of increased sympathetic tone and is a risk factor for cardiovascular events (*64*). In the context of comorbidity, it indicates that the heart is working harder to overcome reduced blood flow from coronary disease, while the high-pressure pulsations may further damage the already stenotic carotid arteries (*65*). (2) Heart rate variability (HRV) measures the beat-to-beat fluctuations in heart rate. A patient with both coronary and carotid disease will often show significantly lower HRV than a healthy individual, even before major symptoms appear (*66*), which presents as a sensitive indicator of overall cardiovascular health. (3) HR response to stress: A blunted rise in heart rate during exercise or an abnormally slow recovery of heart rate after exercise are strong indicators of autonomic dysfunction and underlying coronary artery disease (*67*). (4) Anacrotic and dicrotic notches: Anacrotic notch can become more prominent in conditions like aortic stenosis, which can co-exist; In stiff arteries, the waveform morphology changes, the dicrotic notch may be lower, and the diastolic wave may be less pronounced. Advanced analysis of this contour can reveal details about ventricular-vascular interaction (*68*).

RR and respiratory sinus arrhythmia (RSA): (1) An elevated resting respiratory rate can be a sign of heart failure, a common consequence of advanced CHD (*69*). (2) Blunted or absent RSA is a clear sign of poor vagal tone, which is a hallmark of autonomic dysfunction caused by widespread atherosclerosis (*70*). Monitoring RSA provides a simple, non-invasive way to assess autonomic health and overall cardiovascular risk in co-morbid patients (*71*).

Significance of the cardiac metrics

Our AI algorithm enables the automatic extraction of key cardiac metrics, such as left ventricular internal diameter, stroke volume, ejection fraction, and cardiac output. While these parameters do not directly visualize the coronary or carotid arteries, they reveal the functional impairment of the heart resulting from disease in these vessels. This indirect assessment facilitates the early diagnosis of their comorbidity. Detailed descriptions are as follows.

The left ventricular internal diameter (LVID), measured as LVIDd (diastole) and LVIDs (systole), is a key indicator of cardiac remodeling. Chamber dilation, specifically an increase in LVIDd, often occurs as a compensatory response to chronic ischemia from coronary heart disease (CHD) (*72*). Consequently, discovering an enlarged LVID in a patient with known carotid disease strongly suggests comorbidity. Conversely, in a patient with established CHD, an increasing LVID signals disease progression and a heightened risk for systemic complications like carotid stenosis (*73*).

Stroke volume (SV) and ejection fraction (EF) are intrinsically linked metrics, with EF representing the percentage of blood ejected from the left ventricle per contraction (calculated as SV/End-diastolic volume). A critical early sign of CHD is a regional wall motion abnormality (RWMA). Since CHD often affects the heart unevenly, an echocardiogram can detect hypokinesis (reduced movement) or akinesis (absent movement) in specific myocardial segments supplied by a blocked artery (*74*). Importantly, the global EF may remain normal initially due to compensatory hypercontraction of healthy segments, even while the regional SV is impaired (*75*). The detection of an RWMA is a direct red flag for significant CHD. Therefore, when identified in a patient undergoing evaluation for carotid disease, it provides powerful evidence of silent, co-existing artery disease, elevating the patient's cardiovascular risk profile.

Cardiac output (CO), the total blood volume pumped per minute (CO = SV × Heart rate), serves as a measure of the heart's overall pumping efficiency. Typically, CO is maintained until late disease stages, as the heart increases its rate to compensate for a declining stroke volume. Consequently, a normal CO can be deceptive, masking underlying impairment. However, a low CO in the context of suspected vascular disease is an ominous sign, indicating failed compensatory mechanisms and signifying severe, advanced ventricular dysfunction (*76*). This finding underscores the critical nature of the comorbidity and highlights the urgent need for intervention.

<u>Significance of the carotid metrics</u>

The developed AI algorithm enables the automatic extraction of key carotid metrics, such as arterial geometry variations and dynamic blood pressure fluctuations. These metrics present important measures for assessing carotid status.

Carotid artery circularity describes the roundness of the artery's cross-section. This circularity decreases as atherosclerosis develops, directly reflecting a loss of vascular elasticity. Monitoring this metric provides crucial data for the detection of arterial stiffening, enabling timely intervention.

Artery diameter, specifically the luminal diameter, is a critical metric for assessing vascular health. The development of atherosclerosis is characterized by plaque buildup within the arterial wall, which narrows the lumen, reduces elasticity, and obstructs blood flow. Consequently, tracking changes in arterial diameter provides valuable insight into vessel compliance and hemodynamics, enabling the early detection of cardiovascular pathologies and the assessment of associated risks.

Dynamic blood pressure fluctuations reflect how arteries respond to the pulsatile nature of blood flow, providing insight into arterial stiffness and wave reflections. The carotid arteries are located much closer to the heart and aorta than the arm arteries. Therefore, pressure measured at the carotid artery (or estimated central aortic pressure) more accurately reflects the true pressure exerted on vital organs such as the brain, heart, and kidneys. Carotid or central aortic pressure allows accurate assessment of cardiac workload, as it closely approximates the pressure the heart must pump against to eject blood into the systemic circulation (cardiac afterload) (*77*). This provides a measure of myocardial work. Additionally, changes in the central blood pressure waveform provide valuable support for the early detection of atherosclerosis and other cardiovascular issues (*78*).

<u>Detailed description of the cardiac segmentation/reconstruction model</u>

MTANet-based segmentation model is used to segment cardiac structures from echocardiograms and generate 2D masks, while nnU-Net is employed to reconstruct the complete 3D cardiac structure from these 2D masks. The segmentation codes are available online (https://doi.org/10.5281/zenodo.19470593). It should be noted that nnU-Net is trained at the mask

level, where no substantial difference exists between clinical ultrasound and patch ultrasound data in terms of cardiac structures themselves. Through 2D restoration, we further ensure structural comparability between the two. The segmentation model was built upon the MTANet architecture (*45*). Thanks to the diverse sources of training data and the model's inherent structural assumptions, MTANet can effectively mitigate the impact of domain shift. In practice, rather than performing pure semantic segmentation, this model operates more like identifying a specific structure within an image where its presence is assumed. While this may introduce some false positives, it proves more robust in high-noise image segmentation scenarios. For the MTANet component, we did not fine-tune it specifically for patch ultrasound data. Instead, we directly applied the model trained and externally validated on clinical ultrasound data to perform inference on patch ultrasound images. The details of the models have been disclosed in fig. S29.

Our 3D reconstruction nnU-Net originates from our published study (*79*). This algorithm utilizes high-quality CCTA segmentation results as the gold standard and simulates 2D mask outputs from patch ultrasound by mimicking applicable acoustic windows and slice orientations, thereby training a reconstruction network that accommodates various input slice combinations. In this study, we employ a network model based on the parasternal long-axis single slice for cardiac structure reconstruction. This reconstruction process operates entirely at the mask level, unaffected by the quality of the acquired images, and only requires structural similarity in the input data.

The training details and relevant information of the AI models are described separately below to enhance transparency:

(1) The MTANet segmentation model

(1.1) Training/test data and annotation

We trained a comprehensive cardiac structure segmentation model for echocardiographic parasternal long-axis (PLA) views based on the previously developed MTANet (*45*). The original echocardiography data were sourced from the public dataset EchoNet-LVH (https://echonet.github.io/lvh/) and a self-established dataset (https://doi.org/10.5281/zenodo.19470593). The public dataset contains 12,000 clear PLA view videos, while the self-established dataset includes only 78 frames of end-systolic or end-diastolic images from 59 cases. We annotated the end-systolic and end-diastolic frames from each video, with a small number of frames annotated during cardiac motion. A total of 6,448 frames were annotated, comprising 6,370 frames from 3,208 videos in the public dataset and 78 frames from the self-established dataset. All cardiac ultrasound images were converted to PNG format. Subsequently, the contours of all relevant structures were delineated using the annotation system developed by Demetics Medical Technology Co., Ltd. (software version 4.4.0.1). When videos were unavailable, the annotating physician manually identified the cardiac phase for static images as end-systole, end-diastole, or in motion. The annotation and delineation of regions of interest (ROIs) in the above images were performed by a radiologist with 10 years of ultrasound experience, followed by review and revision by a radiologist with 40 years of ultrasound experience.

Given variations in annotation content across different cardiac phases, for example, the right ventricle (RV) contour was annotated only in end-diastole, and the left atrium (LA) contour only in end-systole, and the presence of ambiguous structural boundaries in some images, we maximized the utilization of available annotated data by performing annotation feature extraction and standardization for each structure separately (left ventricle (LV), LA, RV, aorta (AO), interventricular septum (IVS), left ventricular posterior wall (LVPW), and right ventricular anterior wall (RVAW)). Subsequently, a corresponding MTANet model was trained for each

structure using the public dataset portion as the training set and the self-established dataset portion as the test set. Specifically, for aortic annotations, the segment from the aortic valve annulus to the sinotubular junction was delineated. However, localization issues in defining these boundaries may lead to incomplete segmentation of the aortic portion, which will be addressed and completed in the 2D nnU-Net stage discussed later in this paper.

Since MTANet involves a multi-task learning framework encompassing both segmentation and classification, during training, in addition to segmentation supervision for target structures, classification labels for cardiac phases (end-systole: 0, in motion: 1, end-diastole: 2) were also incorporated as supervision.

- Regarding the left ventricle, the training set contains 6,360 frames, including 3,149 frames at end-systole, 39 frames during motion, and 3,172 frames at end-diastole; the test set includes 11 frames.
- For the left atrium, the training set comprises 3,187 frames, including 3,149 frames at end-systole and 38 frames during motion; the test set contains 29 frames.
- As for the left ventricular posterior wall, the training set has 6,359 frames, consisting of 3,149 frames at end-systole, 39 frames during motion, and 3,171 frames at end-diastole; the test set includes 76 frames.
- In the case of the right ventricle, the training set includes 3,175 frames, with 39 frames during motion and 3,136 frames at end-diastole; the test set contains 48 frames.
- Regarding the interventricular septum, the training set consists of 6,370 frames, including 3,158 frames at end-systole, 39 frames during motion, and 3,173 frames at end-diastole; the test set includes 76 frames.
- For the aorta, the training set contains 2,780 frames, with 35 frames during motion and 2,745 frames at end-diastole; the test set includes 6 frames.

(1.2) Experimental setup

- Data augmentation strategy: During the training phase, we implemented a comprehensive data augmentation scheme to enhance model generalization. Specifically, this included color enhancement (brightness adjustment ±0.2, contrast ±0.1, saturation ±0.1, hue ±0.1) and spatial transformation augmentation (random 90-degree rotations, vertical flipping with a probability of 0.5, and horizontal flipping with a probability of 0.5). All training images and their corresponding annotations were uniformly resized to a resolution of 352×352 pixels and normalized using the ImageNet standard statistics. During the validation and testing phases, only necessary resizing and normalization were performed, with no random augmentation operations introduced.
- Training parameter configuration: The experiment was configured for a total of 200 training epochs, with training resuming from the 15th epoch of a pre-trained model, resulting in 185 actual training epochs. The AdamW optimizer was employed with an initial learning rate of 1e-5 and a weight decay set to 1e-4. A step-decay learning rate scheduler was used, reducing the learning rate to 0.1 times its previous value every 15 epochs. The training batch size was set to 16, while the testing batch size was set to 1. A multi-scale training strategy was also implemented, randomly selecting one of three scaling factors, [0.75, 1, 1.25], in each iteration.
- Loss function design: The model adopts a multi-task learning framework. For the segmentation task, a composite loss function combining weighted binary cross-entropy and weighted intersection-over-union (IoU) is applied across four lateral output layers of the decoder. The classification task is formulated as a regression problem to predict cardiac phase parameters,

with its loss function consisting of a linear combination of mean squared error loss and L1 loss. The total loss is simply the sum of the segmentation layer losses and the classification loss, with no special weighting coefficients applied.

- Model architecture and training details: The MTANet architecture is employed. During training, gradient clipping with a threshold of 0.5 is implemented to prevent gradient explosion. A checkpoint is saved after each training epoch, and comprehensive evaluations are performed on both the internal test set and an external validation set.

(1.3) Segmentation test results

After finalizing the model parameters, we evaluated the model on the test set by calculating the Dice coefficient. The overall average Dice coefficient was 0.843±0.029, and the segmentation of each structure demonstrated consistent performance across institutions (fig. S29).

Given these results, we directly applied this segmentation pipeline to patch-based ultrasound images. Given the current lack of a large, annotated dataset for patch-based ultrasound, using this model represents a balanced compromise, and arguably the most feasible option, especially considering the need to avoid overfitting on limited data.

(2) The nnU-Net reconstruction model

(2.1) Training/test data and annotation

Three-dimensional whole heart shape reconstruction was achieved by the nnU-Net model (fig. S30). For training and testing the mode, we randomly selected 2,200 patients with chest pain from Jinhua Municipal Central Hospital (our collaborator of this work) and collected their corresponding coronary computed tomography angiography (CCTA) images. A publicly available CCTA segmentation network (*79*) was applied to the dataset in this study. Both diastole and systole images from 2,183 patients were successfully processed, with each frame annotated for 10 structures: left ventricle (LV), left ventricular myocardium (LVM), right ventricle (RV), left atrium (LA), right atrium (RA), aortic root (AOR), pulmonary artery (PUL), left pulmonary vein (LPV), right pulmonary vein (RPV), and left atrial appendage (LAA). Subsequently, an experienced radiologist manually reviewed the automatically generated segmentation results, focusing on cases with apparent segmentation failures. Finally, 2,169 patients were confirmed to have acceptable segmentation across all images. To ensure dataset balance, we ultimately randomly selected 1,000 female and 1,000 male cases, with both groups exhibiting similar age distributions. We trained our models using simulated sparse volumes and dense ground truth volumes from 1000 pairs of CCTA images in best diastolic and systolic frames and tested them on another 1000 pairs.

(2.2) Experimental setup and test results

To evaluate the impact of using one or multiple acoustic windows, and the effect of cardiac motion on reconstruction performance, we designed four test groups:

- Single-view input data, including the four-chamber view (4CH) and the papillary muscle-level short-axis view (PLA).
- Input data consisting of two orthogonal views from the same acoustic window, including the four-chamber view plus the two-chamber view (4CH + 2CH), and the papillary muscle-level short-axis view plus the apical-level short-axis view (PLA + PSA).
- Multi-view input data from two different acoustic windows, including the four-chamber view plus the papillary muscle-level short-axis view (4CH + PLA), as well as all four views combined.
- Input data containing all four views along with motion artifacts.

Model performance was evaluated using the Dice Score and the Hausdorff Distance, with the respective results presented in fig. S30. Results demonstrated that integrating two orthogonal imaging windows (apical and parasternal) provided the highest reconstruction accuracy, with a mean Dice score of 0.95 for the four chambers and the left ventricular myocardium. The most cost-effective approach utilized the parasternal long-axis (PLA) view, which produced a Dice score of 0.87 for all seven labels and proved particularly effective for aortic reconstruction. Additionally, our motion simulation strategy revealed that reconstruction accuracy was well preserved despite the presence of substantial cardiac motion. In this work, we used the PLA view modeling.

Detailed description of the carotid segmentation model

In light of the scarcity of high-quality manually annotated time-series data at the initial stage of the study, and to achieve precise automated segmentation of the carotid artery region under limited data conditions, this work adopted a targeted model training strategy. During the model design phase, priority is given to ensuring the accuracy and effectiveness of the segmentation task, without initially focusing on generalization capability as the core optimization objective. Instead, a controlled overfitting training approach is employed to fully exploit the anatomical structural features embedded in the small annotated dataset. The specific implementation process is as follows: For each case of carotid artery time-series imaging data, 50 representative images were selected via random sampling to construct a dedicated training subset. This subset was then meticulously annotated by experienced imaging specialists to ensure the accuracy and consistency of the labels. Subsequently, using this annotated subset as training samples, the model was iteratively trained until an overfitted state is reached, enabling the model to thoroughly learn the morphological features, grayscale distribution, and boundary information of the target region. Upon completion of training, the overfitted model was directly applied to the remaining unannotated frames of the same case, thereby achieving automated segmentation of the entire time-series data.

It should be noted that the aforementioned strategy represents a phased approach developed under the initial data constraints. In the future, by constructing a comprehensive, multi-center, and multi-sample dataset, and employing standardized machine learning optimization methods, such as K-fold cross-validation, data augmentation (e.g., rotation, scaling, grayscale perturbation), and regularization, the model's adaptability to varying data distributions, individual differences, and imaging acquisition conditions will be systematically enhanced. This, in turn, will achieve significant improvements in the model’s generalization performance, thereby laying a solid foundation for its clinical application and broader implementation.

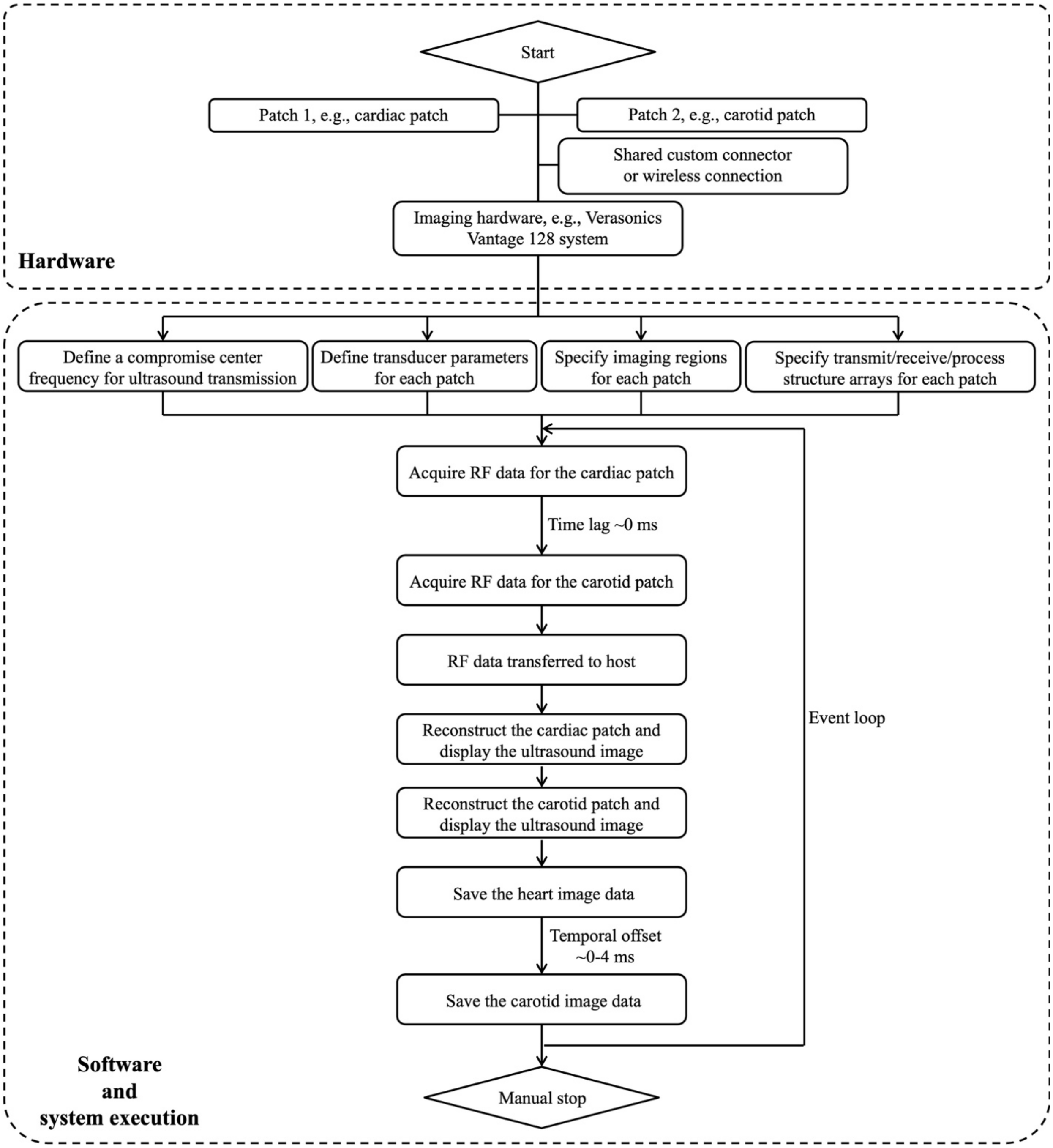

Fig. S1.

Dual-patch synchronous imaging strategy. For ease of understanding, one patch is assumed for cardiac imaging, while the other is for carotid imaging. The proposed dual-patch synchronous imaging strategy is explained as follows. (1) Hardware requirement: The cardiac and carotid patches are connected to the same back-end imaging hardware (e.g., the Verasonics Vantage 128 system) via a shared custom connector, each patch is pre-assigned with a distinct array address range (e.g., 1-64 for the cardiac patch and 65-128 for the carotid patch, see fig. S8). (2) Software requirement: Synchronous imaging algorithm was developed, with the transmission protocols, reception parameters, and reconstruction algorithms customized for each patch. (3) System execution: The RF (radiofrequency) data from both cardiac and carotid patches were acquired

sequentially within the same frame. This frame was then transmitted to the imaging hardware host for successive reconstruction and display before being saved locally on the host computer. The acquisition loop operates continuously until manually terminated.

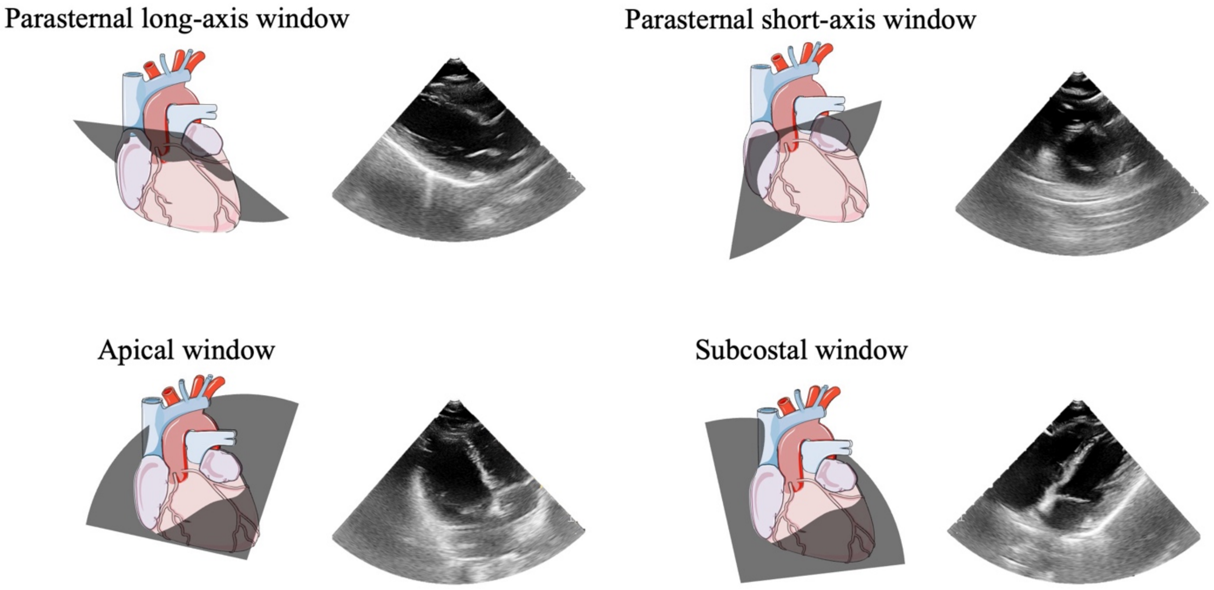

Fig. S2.

Four common acoustic windows for cardiac ultrasound imaging. Illustration of each window and the corresponding ultrasound images. Ultrasound images were captured using commercial ultrasound equipment (Philips EPIQ 7C). The heart models were illustrated using PowerPoint.

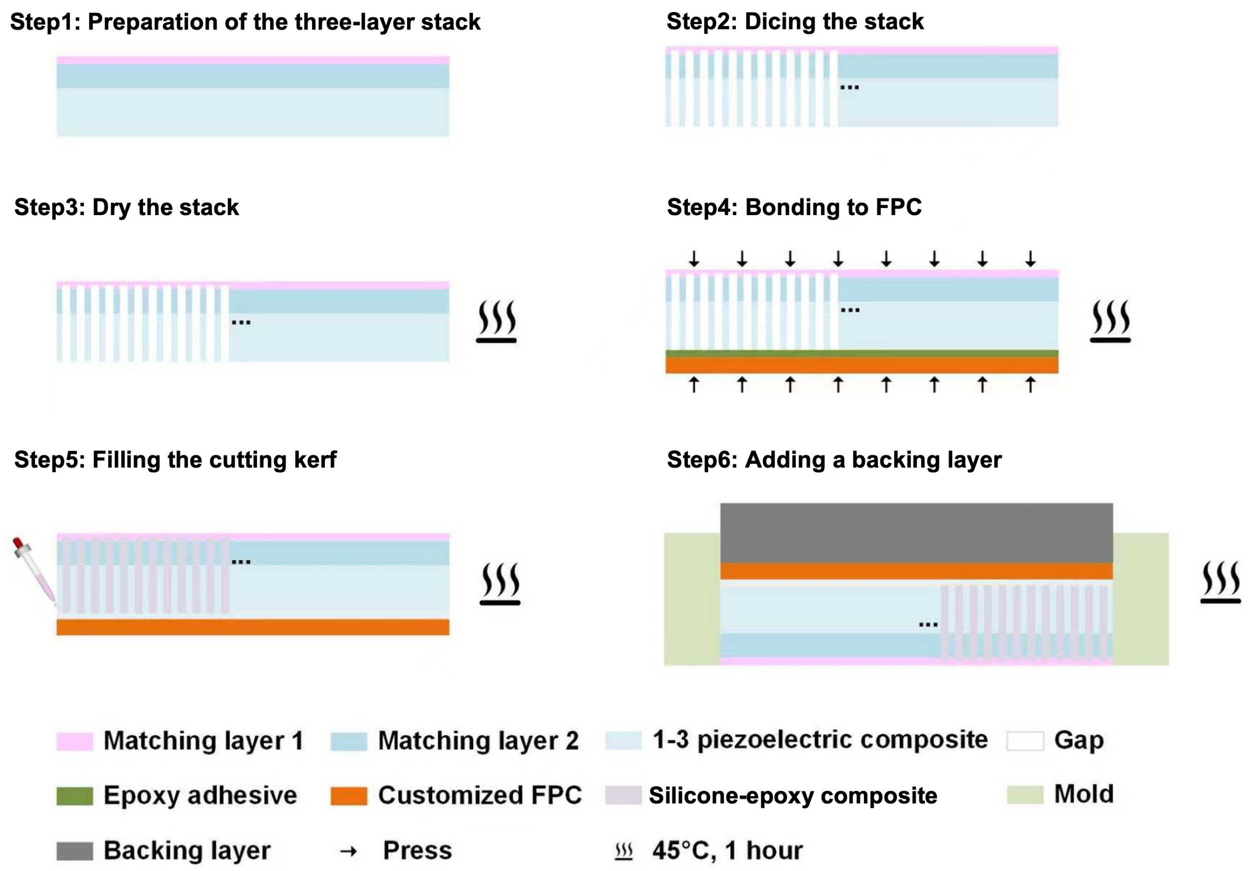

Fig. S3.
Fabrication process for the cardiac and carotid ultrasound patches.

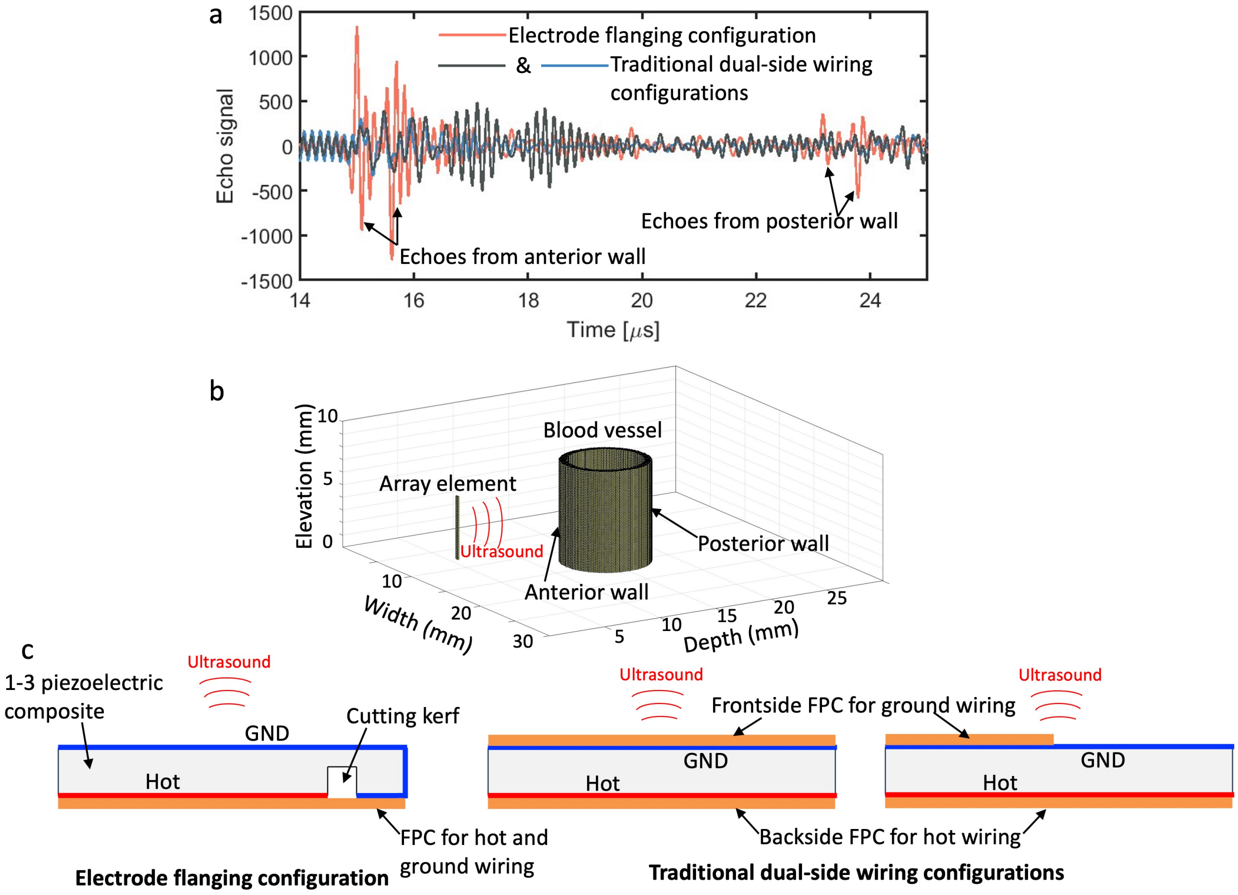

Fig. S4.

Improve the imaging quality by the electrode flanging process. **a**, Numerical simulations using the k-Wave toolbox demonstrate that the electrode flanging configuration provides superior carotid artery echo signal identification compared to conventional dual-side wiring methods. **b**, The simulation model implemented in k-Wave, showing an array element facing towards the carotid artery. **c**, Comparison of the electrode flanging configuration and traditional dual-side wiring configuration. In this work, the patches were fabricated using an electrode flanging process, enabling both the hot and ground electrode wires to be routed from the bottom side of the array element. This design allows the top surface to be dedicated to matching layers for optimized ultrasound transmission. In conventional ultrasound patches, electrode wires typically extend from both sides of the array element. This configuration interferes with matching layer design and compromises imaging quality.

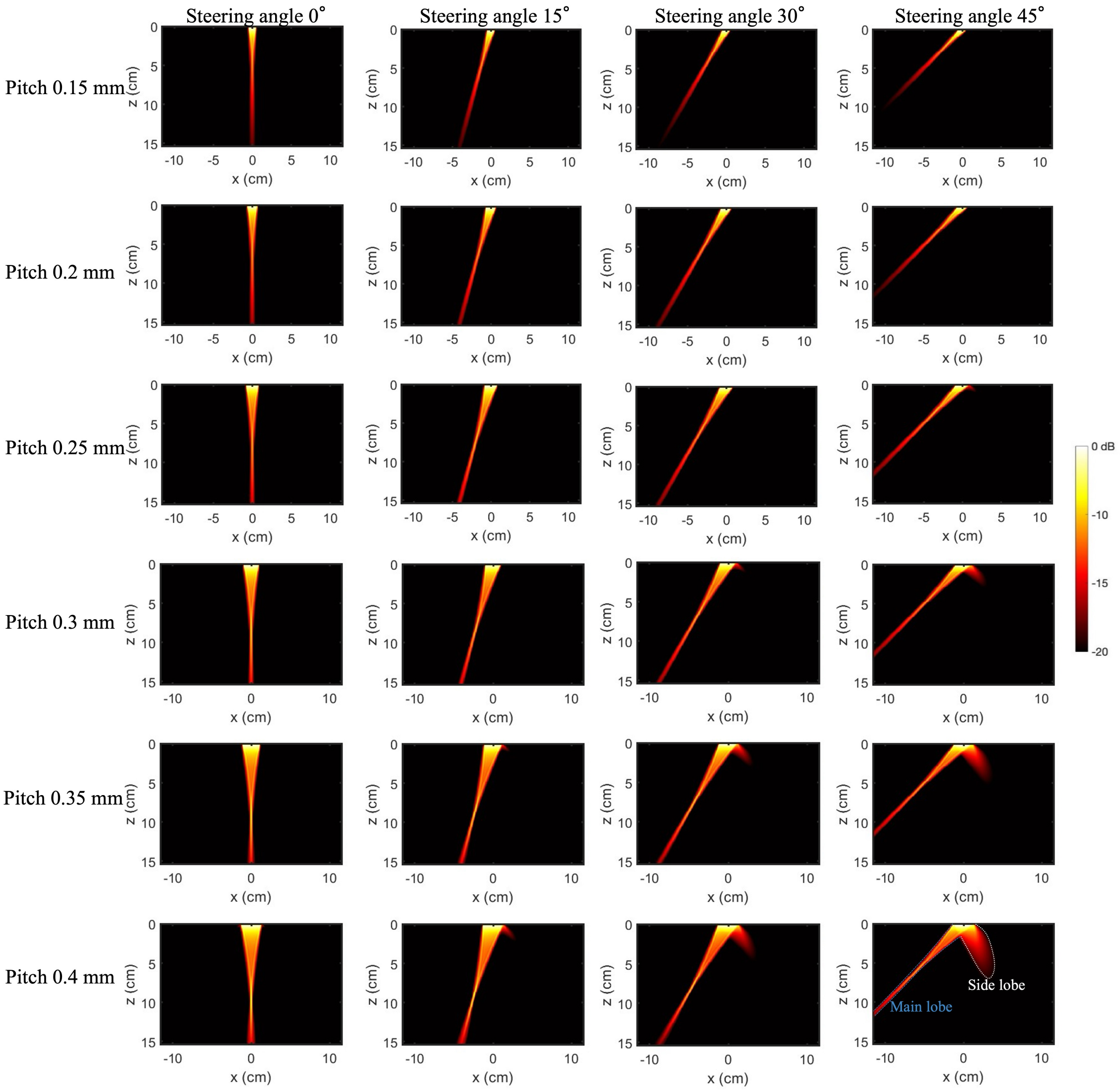

Fig. S5.

Effects of the array pitch on side lobes. Simulated acoustic pressure distributions for phased-array imaging with focused transmits, evaluated across varying array pitches and steering angles. Results were obtained at the center frequency of 3 MHz and focus distance of 10 cm. Results demonstrate that an array pitch smaller than 0.25 mm (approximately half-wavelength) is optimal for phased-array imaging with focused transmits, as it suppresses side lobe generation. Here, side lobes are undesired secondary beams of acoustic energy that propagate at angles offset from the main ultrasound beam (main lobe). They are an inherent artifact of transducer array beamforming and can degrade image quality.

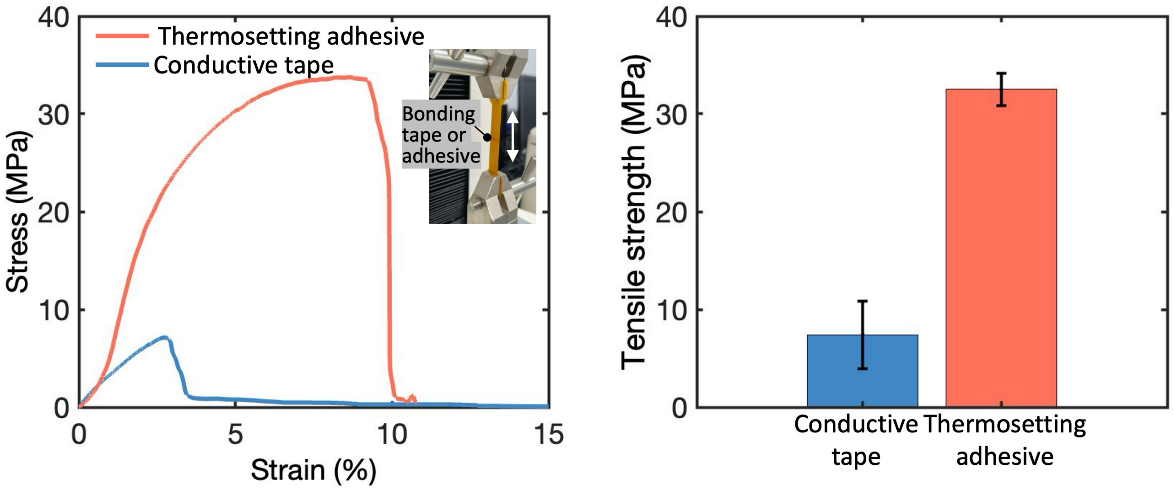

Fig. S6.

Tensile strength tests of the bonding adhesive between the transducer array and flexible printed circuit. Test samples were prepared by bonding two FPCs either using the thermosetting adhesive (Epo-Tek 301) or anisotropic conductive tape (3M9703). The epoxy adhesive enhanced tensile strength by approximately 4-fold, achieving >30 MPa, which was selected for ensuring stable array connectivity. Five replicates were tested for each bonding material in the right panel, with error bars indicating standard deviations.

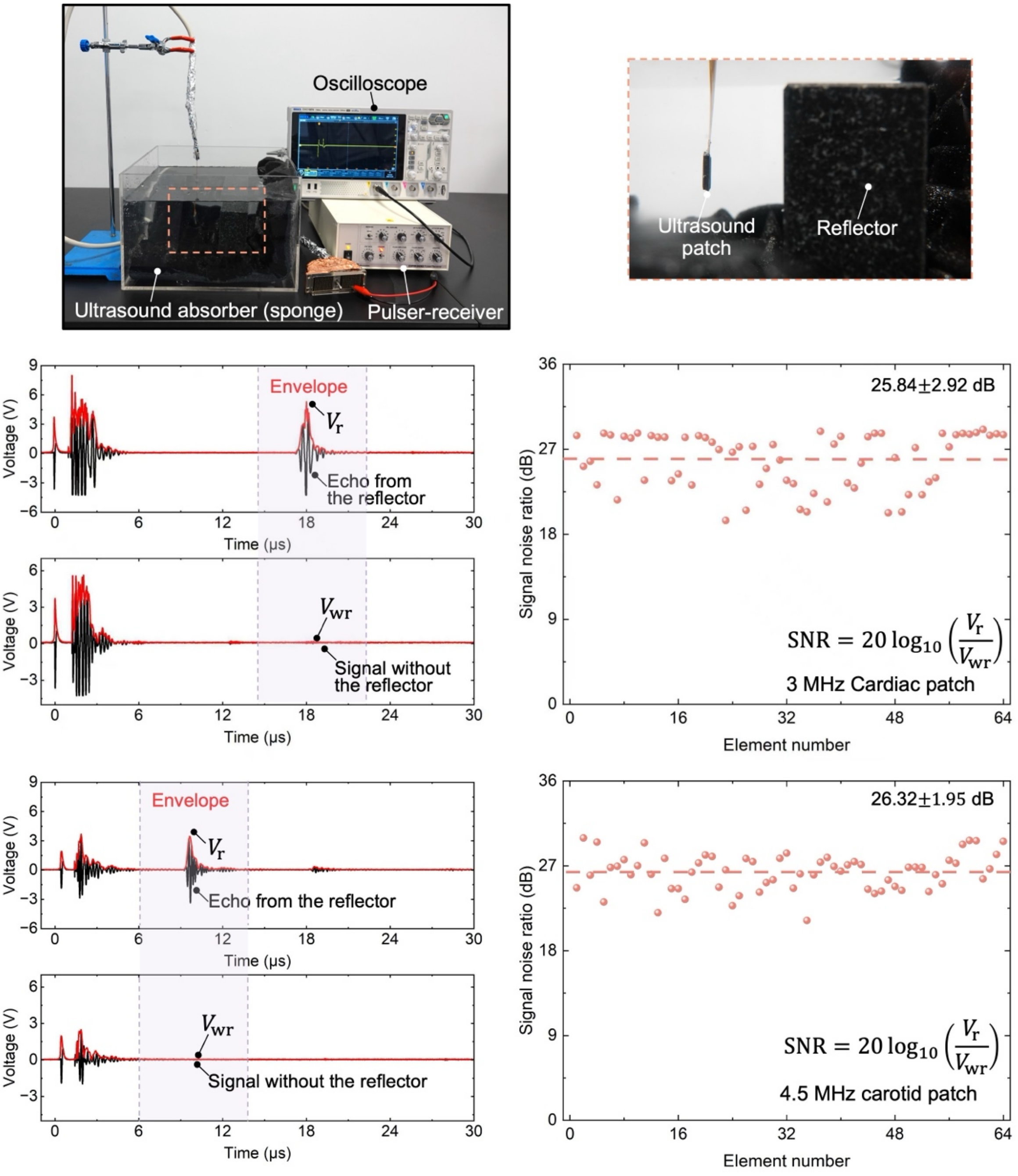

Fig. S7.

SNR (signal to noise ratio) measurements of the cardiac and carotid patches. Top panel: experimental setup for measurement. Middle panel: SNR of the cardiac patch. The radiofrequency signal from a single array element was recorded with and without a reflector, and its envelope was extracted to determine the signal amplitude (V_r) for SNR calculation. The cardiac patch exhibited an SNR of 25.84 ± 2.92 dB. Bottom panel: SNR of the carotid patch, showing a value of 26.32 ± 1.95 dB.

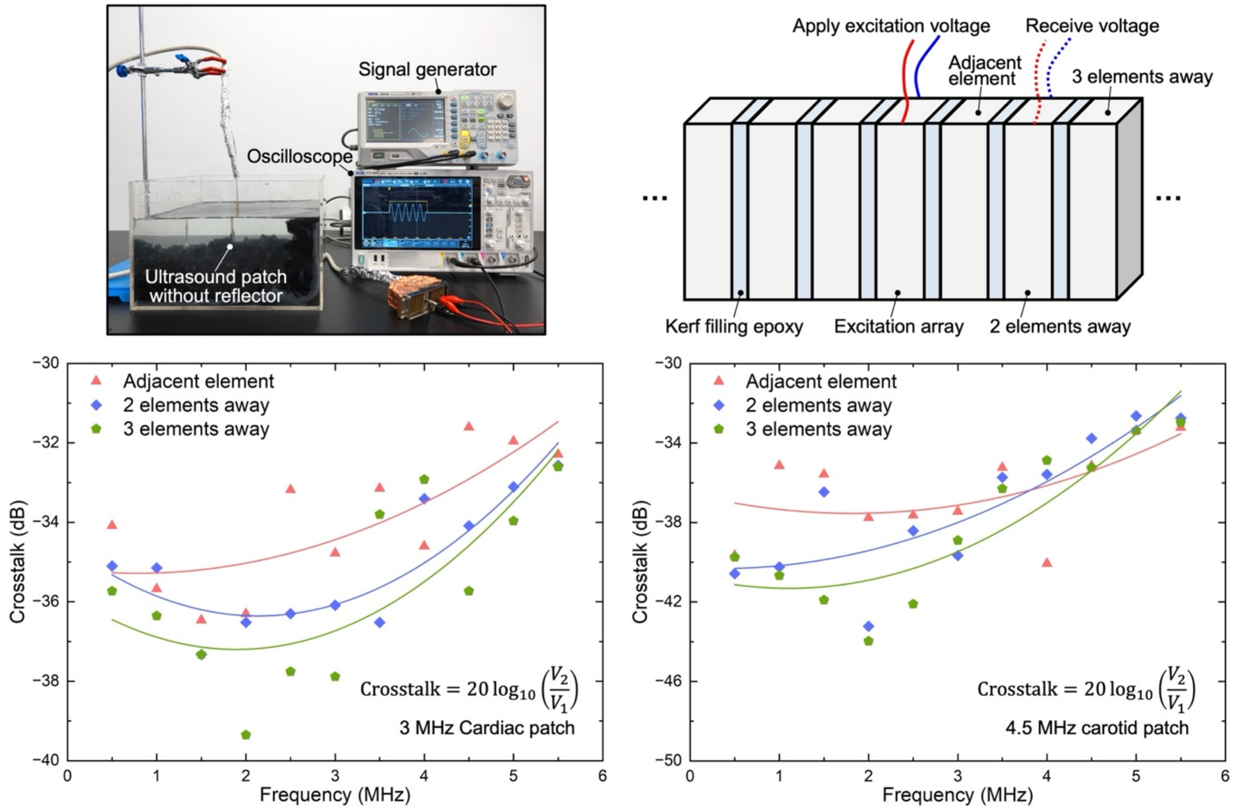

Fig. S8.

Crosstalk measurements of the cardiac and carotid patches. Top panel: experimental setup for measurement. Crosstalk was measured by exciting a randomly selected array element and recording the radiofrequency signal from adjacent elements. The excitation was a 5-cycle burst with an amplitude (V_1) of 2.5 V. The induced voltage in the adjacent element (V_2) results from vibration propagating along the kerf filling direction. The cardiac patch exhibited a crosstalk of approximately -35 dB at 3 MHz, while the carotid patch showed approximately -34 dB at 4.5 MHz.

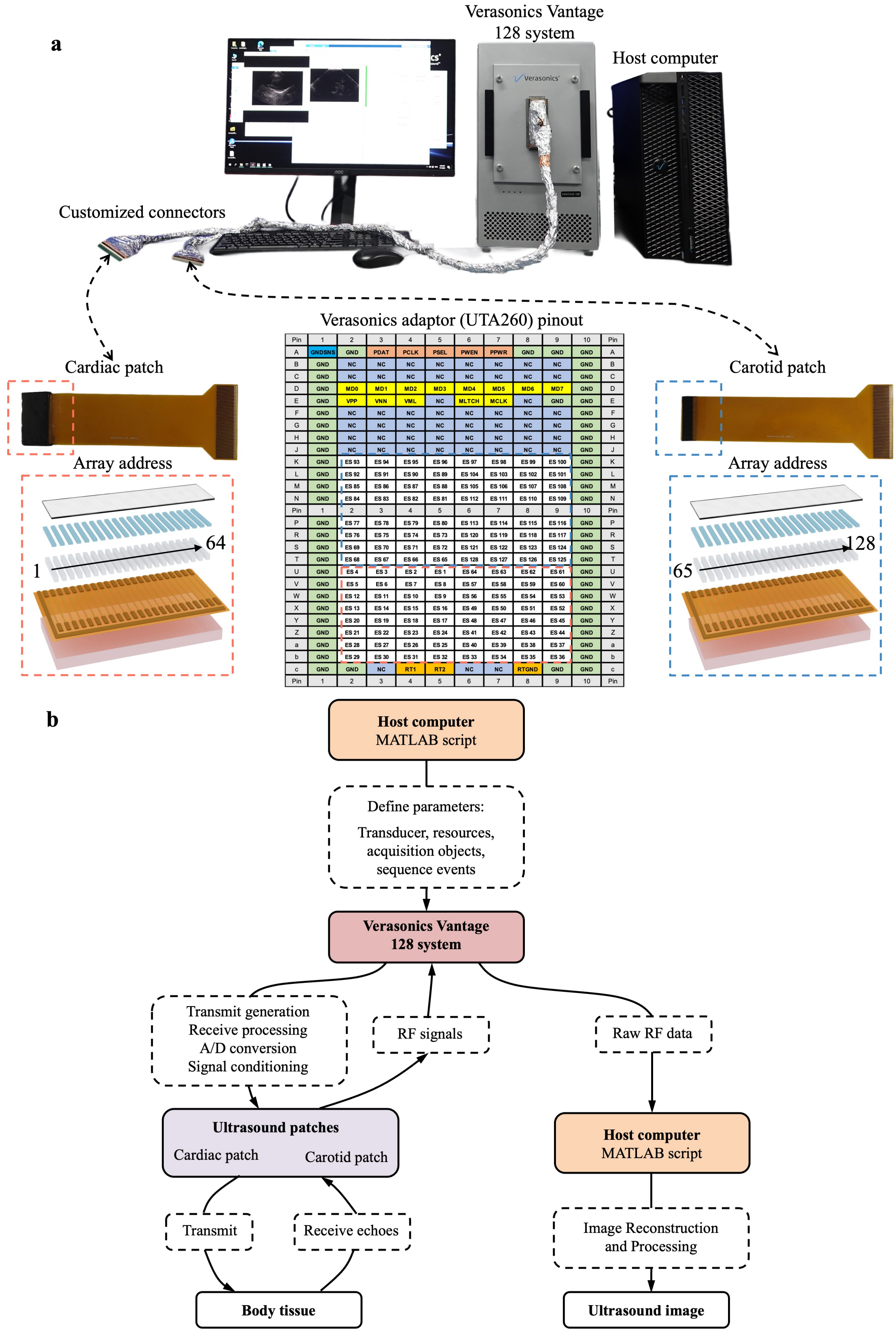
a
Verasonics Vantage 128 system
Host computer
Customized connectors
Verasonics adaptor (UTA260) pinout
Cardiac patch
Carotid patch
Array address
Array address
1
64
65
128
b
Host computer
MATLAB script
Define parameters:
Transducer, resources, acquisition objects, sequence events
Verasonics Vantage 128 system
Transmit generation
Receive processing
A/D conversion
Signal conditioning
RF signals
Raw RF data
Ultrasound patches
Cardiac patch
Carotid patch
Host computer
MATLAB script
Transmit
Receive echoes
Image Reconstruction and Processing
Body tissue
Ultrasound image

Fig. S9.

System configuration for performing synchronous ultrasound imaging. **a**, Array address assignment for two patches. Two patches are connected to the back-end controller (Verasonics Vantage 128 system) via customized connectors. The cardiac patch is assigned with array address from 1 to 64, while the carotid patch is with 65 to 128. The customized connector is designed to match the array address to the pin address of the back-end controller. **b**, Work flow chart of the system. Sequence callback of two patches for RF acquisition, reconstruction and data save are programed based on the synchronous imaging strategy in fig. S1.

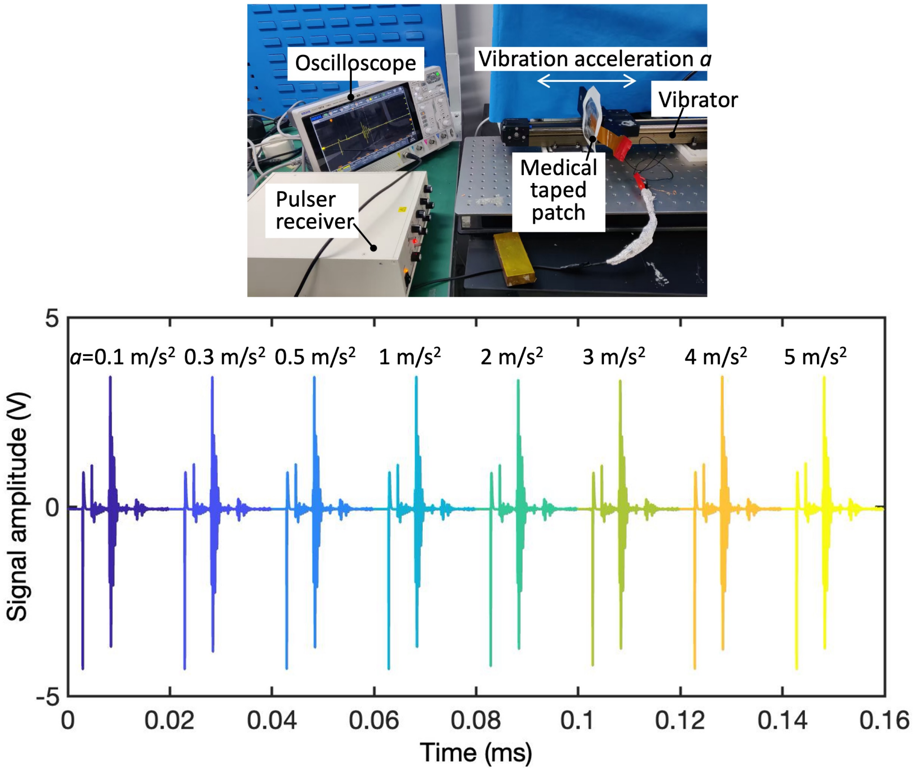

Fig. S10.

Stable ultrasound signal acquisition by medical taped patches. The ultrasound patch was acoustically coupled to a PDMS substrate (for mimicking the skin) using a medical-grade adhesive film (3M Tegaderm), with ultrasound transmission gel applied at the interface. The assembled device was securely mounted on an electrodynamic shaker, and pulse-echo signals were acquired from the PDMS under controlled vibrational accelerations (0.1-5 g) to assess the stability characteristics. As can be observed, stable ultrasound signal acquisition can be ensured even under the acceleration of 5 m/s^2 (0.5 g, g is the acceleration of gravity), which is equivalent to the acceleration by brisk walking, low-intensity cycling, jogging or light fitness training.

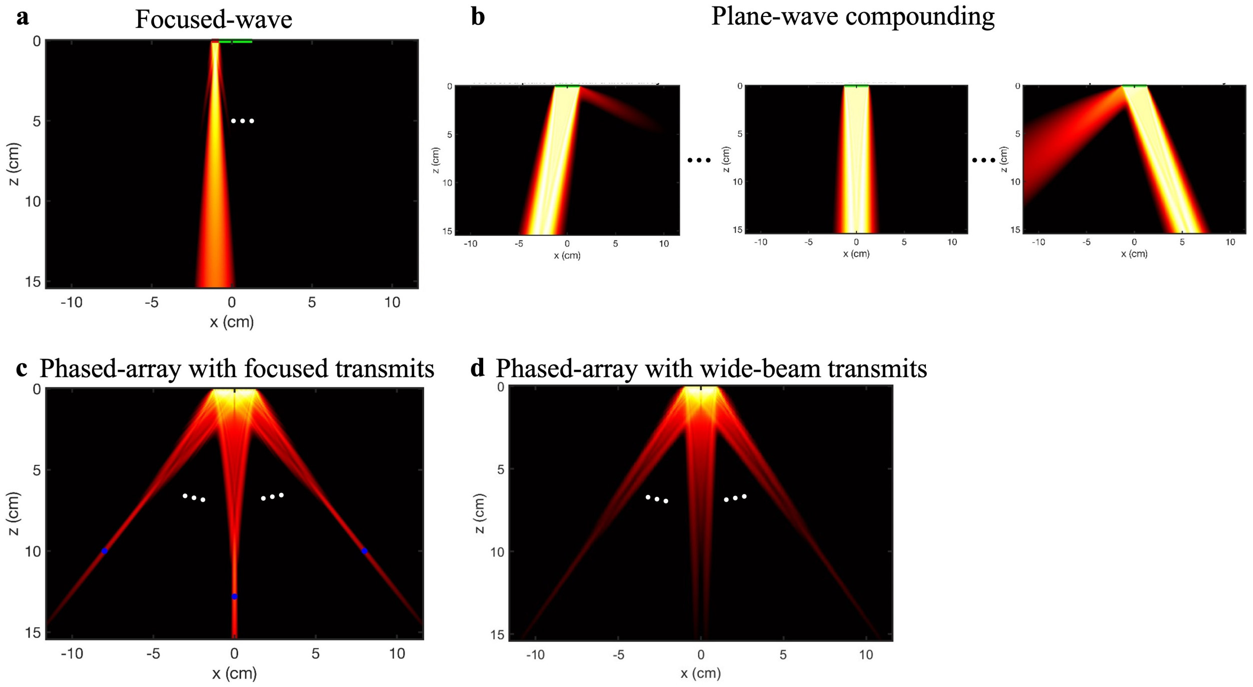

Fig. S11.

Comparison of different imaging methods. The acoustic pressure distributions are plotted in the figures to illustrate the working principle of each imaging method. **a** Focused-wave imaging is performed by sequentially acquiring scanlines along the transducer's pitch direction, with each scanline formed by transmitting and receiving a dynamically focused beam along the depth axis. **b** Plane-wave compounding imaging is achieved by transmitting unfocused plane waves across the entire imaging field in a single shot, then combines multiple angled acquisitions to improve image quality. Phased-array imaging uses electronic beam steering and dynamic focusing with an array of small transducer elements to generate real-time, sector-shaped images. Unlike mechanical scanners, it achieves beam direction and focus control purely through precisely timed excitation delays. The main difference between **c** and **d** lies in their transmit beamforming strategies. Phased-array imaging with focused transmits uses sequential narrow and focused beams to achieve high resolution at specific depths, while phased-array imaging with wide-beam transmits uses unfocused beams to achieve high imaging resolution uniformly distributed along the scanline.

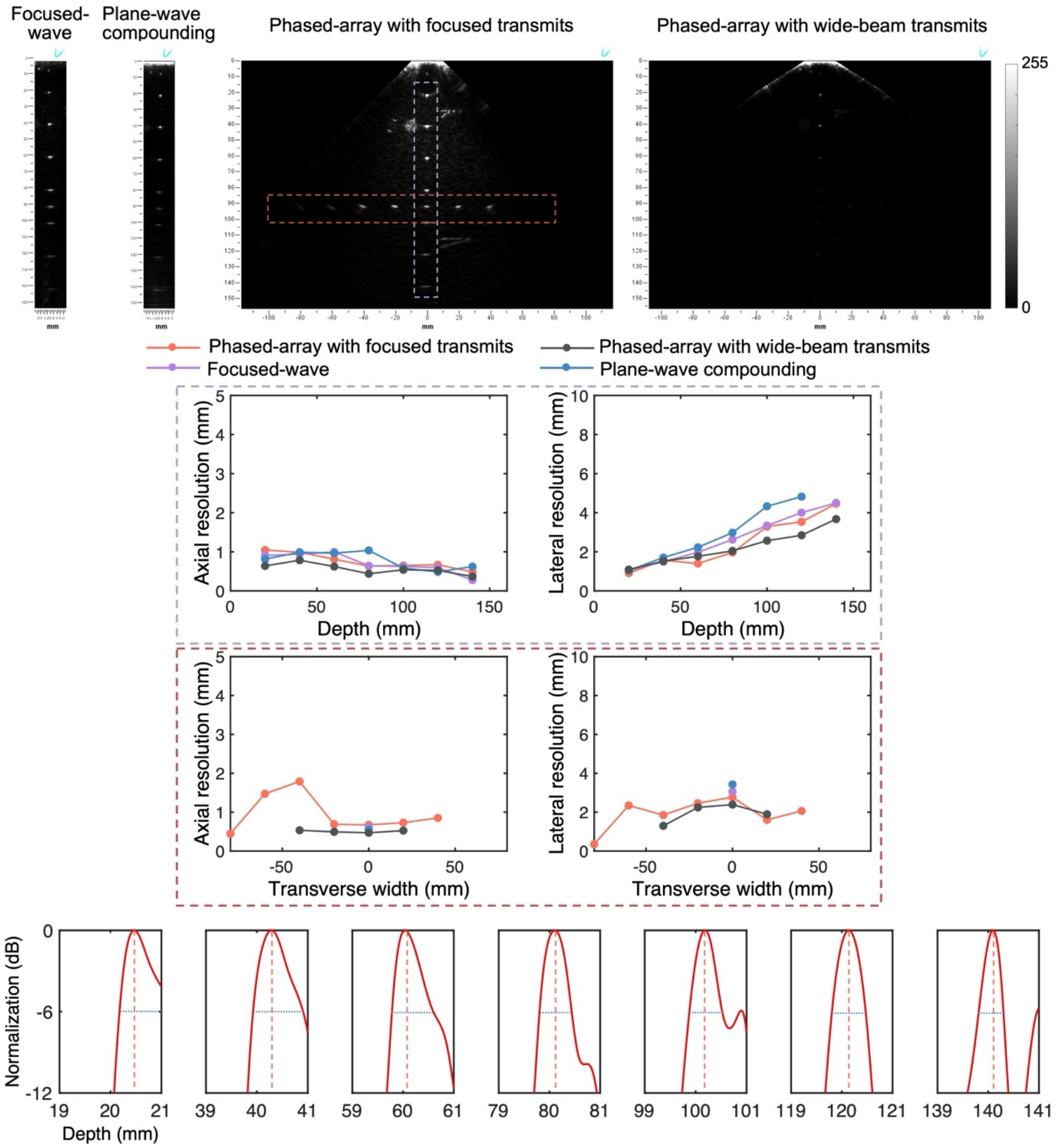

Fig. S12.

Cardiac patch characterization using the ultrasound phantom. Images were acquired with a standard phantom (Model 054GS, CIRS). The heart patch requires wide field of view and deep ultrasound penetration. In the axial direction (marked in purple), the phantom wires (100 um in diameter and visible as white dots in ultrasound images) exhibit similar trends in measured axial and lateral resolution among different imaging methods: the axial resolution remains nearly constant, while the lateral resolution degrades with increasing imaging depth. However, in the lateral direction (marked in yellow), the phased-array imaging method with focused transmits captures the full view of the phantom wires, and this method was selected as the imaging method for the cardiac patch.

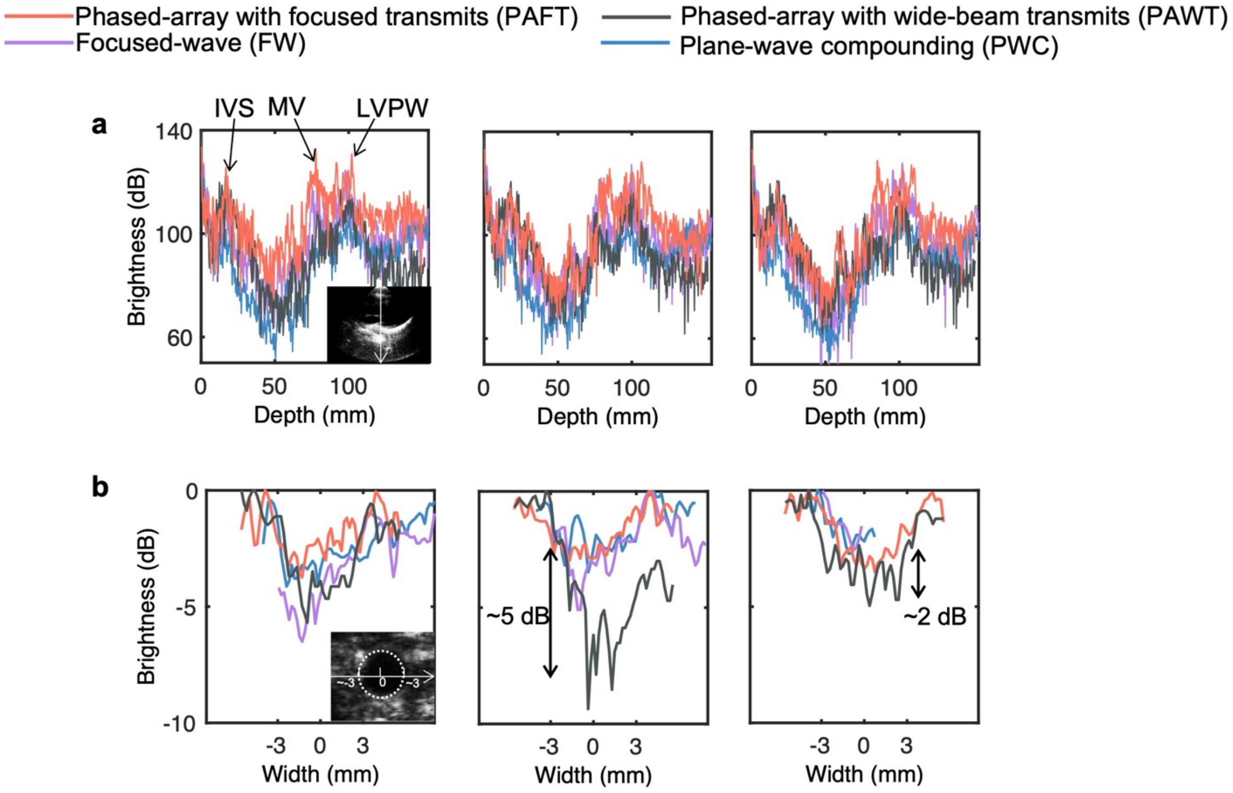

Fig. S13.

Contrast characteristics of the cardiac and carotid patches for in vivo imaging. **a**, Consistent high-contrast echoes across postures (forward stretching, deep breathing, backward stretching respectively from left to right, Fig. 2C) confirmed the suitability of PAFT for cardiac imaging. **b**, High imaging contrast of the PAWT imaging method across postures (left twisting, normal, right twisting, Fig. 2D).

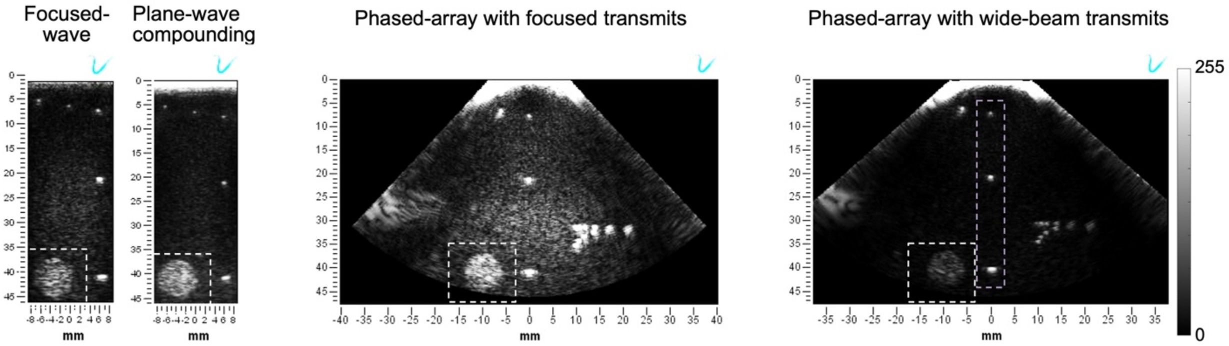

Imaging methods	Focused-wave	Plane-wave compounding	Phased-array with focused transmits	Phased-array with wide-beam transmits
Contrast ratio (dB)	6.62	6.12	7.78	**9.07**

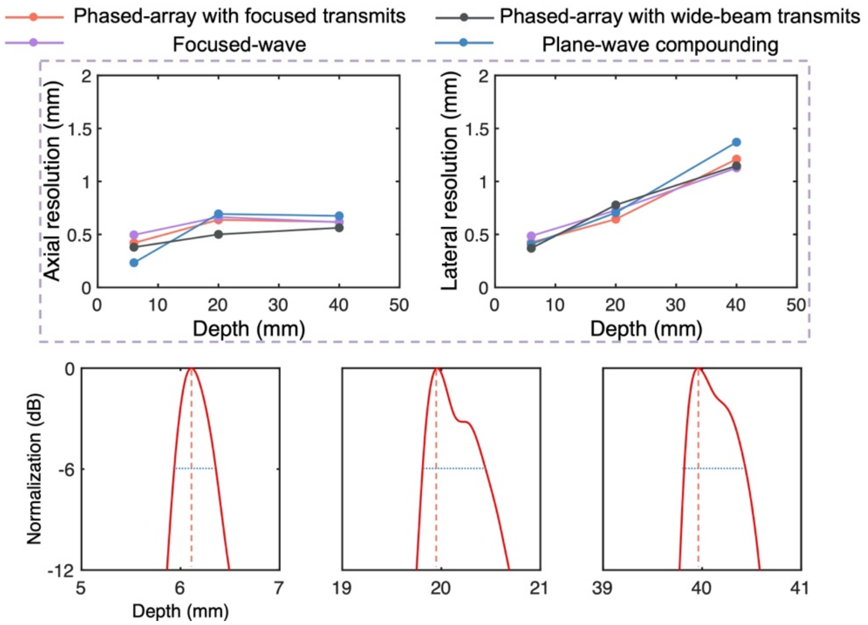

Fig. S14.

Carotid patch characterization using the ultrasound phantom. Images were acquired with a standard phantom (Model 054GS, CIRS). The carotid patch requires both high imaging contrast for clear visualization of the target artery and a wide field of view to prevent measurement loss of the target during physical movements. The contrast ratio quantifies the difference in signal intensity between a region of interest and its surrounding background in an ultrasound image. Results show that phased array imaging with wide-beam transmits demonstrate the highest contrast ratio with a wide field of view. In addition, this imaging method presents the best imaging resolutions, thus was selected for carotid imaging.

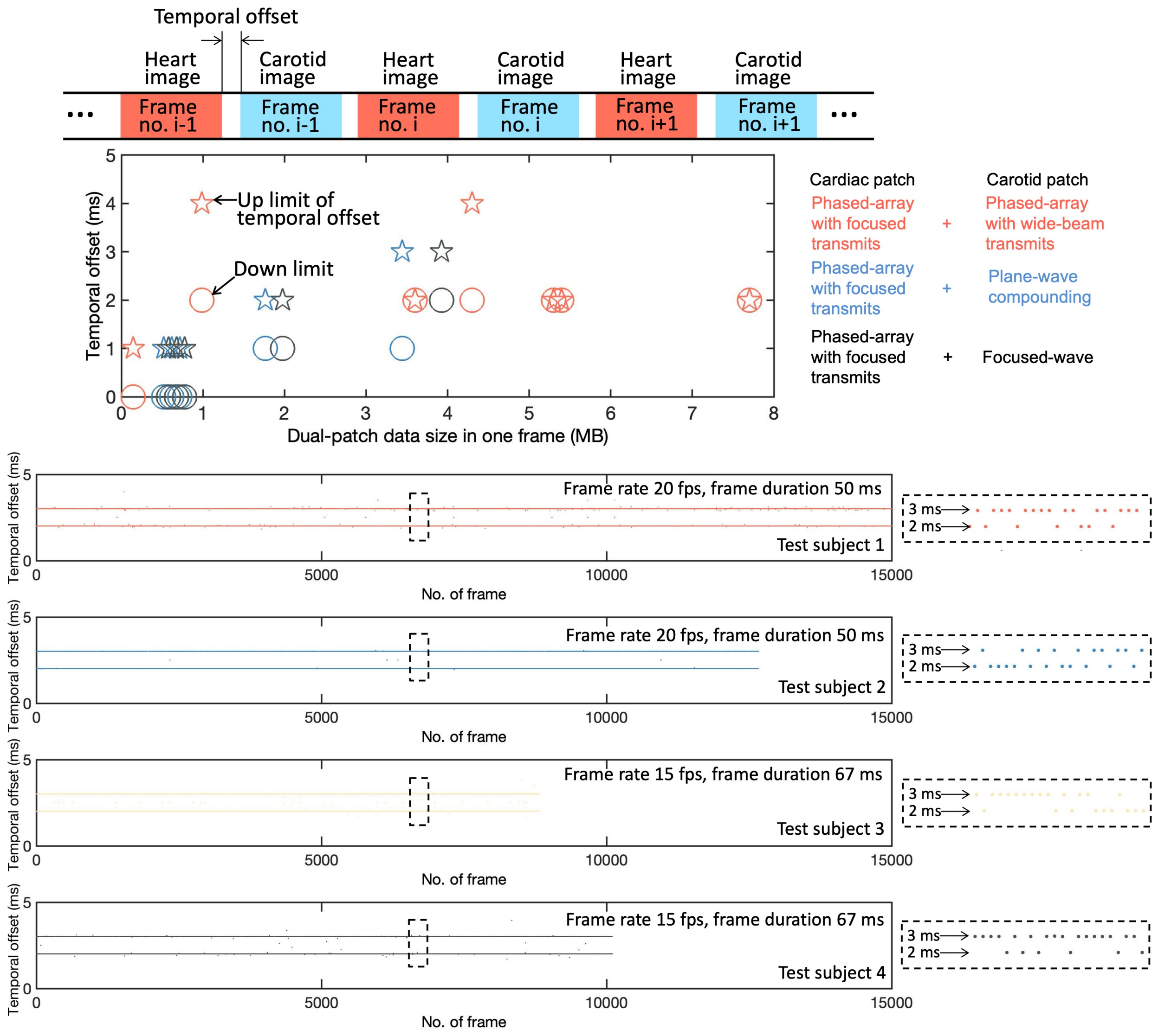

Fig. S15.

Validation of synchronous ultrasound imaging. For dual-patch synchronous imaging, sequential data acquisition by two patches introduces an inherent temporal offset between image captures (top figure). This precludes perfect synchronization in the strictest sense. However, comprehensive testing across multiple imaging methods, fields of view, parameter sets, and frame counts demonstrates that while image data size is a key influencing factor, its impact on the temporal offset remains minimal, varying within a 0-4 ms range (middle figure). Considering the typical frame duration for performing dual-patch imaging (~50 ms), the temporal offset only contributed to ~0-8%. Analysis of collected image data following the exercise protocol across 10,000 frames reveals a consistent temporal offset of 2-3 ms, which is limited when compared to the frame duration (>~50 ms). It is considered that the system achieves functionally synchronous imaging for practical purposes.

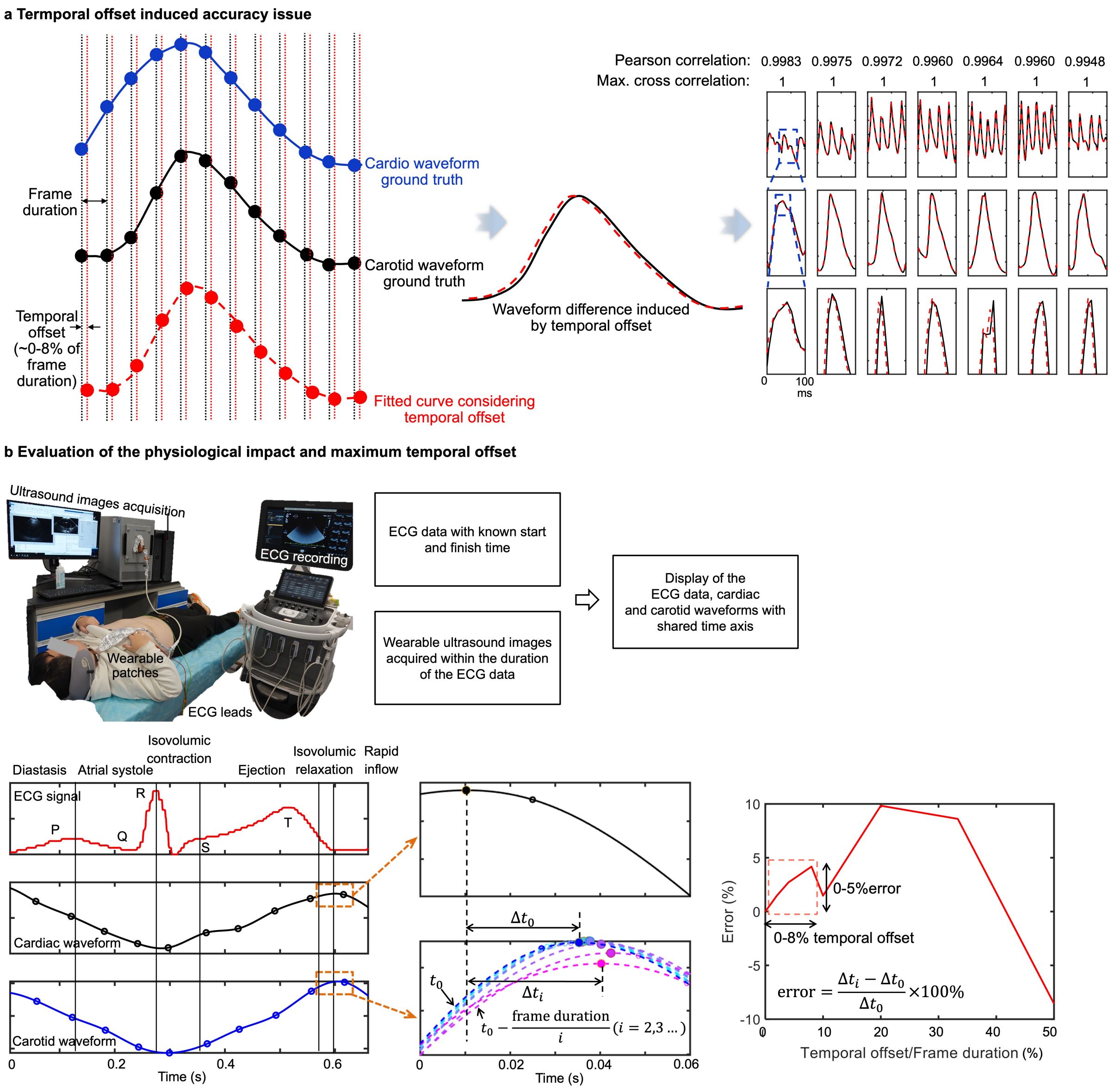

Fig. S16.

The temporal offset induced waveform accuracy issue and the corresponding physiological impact. **a**, Impact of temporal offset on waveform accuracy. Assume we have temporally synchronized ground-truth cardiac and carotid waveforms (LVPW motion and arterial pulsation waveforms, respectively), the measurements acquired by the cardiac and carotid patches appear as scatter points distributed along these waveforms, with point density determined by the frame rate. In practice, since the ground-truth waveforms are unknown, we estimate them by performing curve fitting on the scatter points obtained from the ultrasound patches. If the scatter points contain a temporal offset, the resulting fitted waveform will differ slightly from that fitted without offset, leading to a waveform accuracy concern (fig. S16a). To evaluate the impact of such temporal offsets, carotid blood pressure waveforms (Fig. 5F) were tested. Artificially introducing a 4 ms temporal shift produced a subtle change in the waveform shape. The Pearson correlation between the original and shifted curves remained nearly 1, indicating that the effect on waveform

morphology is minimal. **b**, Evaluation of the physiological impact. The ECG was measured synchronously with the ultrasound patches, and all waveforms were processed along a shared time axis. The ECG delineates key cardiac periods: diastasis, atrial systole, isovolumic contraction, ejection, isovolumic relaxation, and rapid inflow. These phases correspond directly to mechanical events visible in the cardiac and carotid waveforms. For example, during atrial systole, left ventricular (LV) volume increases and the LVPW extends deeper; during isovolumic relaxation and rapid inflow, LV volume reaches its minimum and then gradually rises, accompanied by shallower LVPW motion. The physiological impact of temporal offset arises primarily from two factors: heart rate (or pulse rate) estimation, and the timing difference between the cardiac waveform peak and the adjacent carotid waveform peak, which reflects heart-carotid pulse transmit time (PTT). Given the nearly perfect correlation (~1) between original and temporally shifted waveforms, the effect on heart rate is negligible. However, the influence on PTT estimation warrants closer examination. Analysis shows that a temporal offset within 50% of the frame duration yields a PTT estimation error within ±10%. In the current setup, a temporal offset of 0-8% corresponds to a PWV error of 0-5%. Healthy adults typically exhibit a heart -carotid PTT of ~36 ms. In patients with carotid stenosis or coronary artery disease, PTT is often reduced by roughly 10 ms (~27% of the normal value) due to localized decreases in tissue elasticity. Across the studied temporal offset range, the resulting PTT error does not exceed this 27% pathological variation. Therefore, the error of 0-5% introduced by our current setup remains within an acceptable margin.

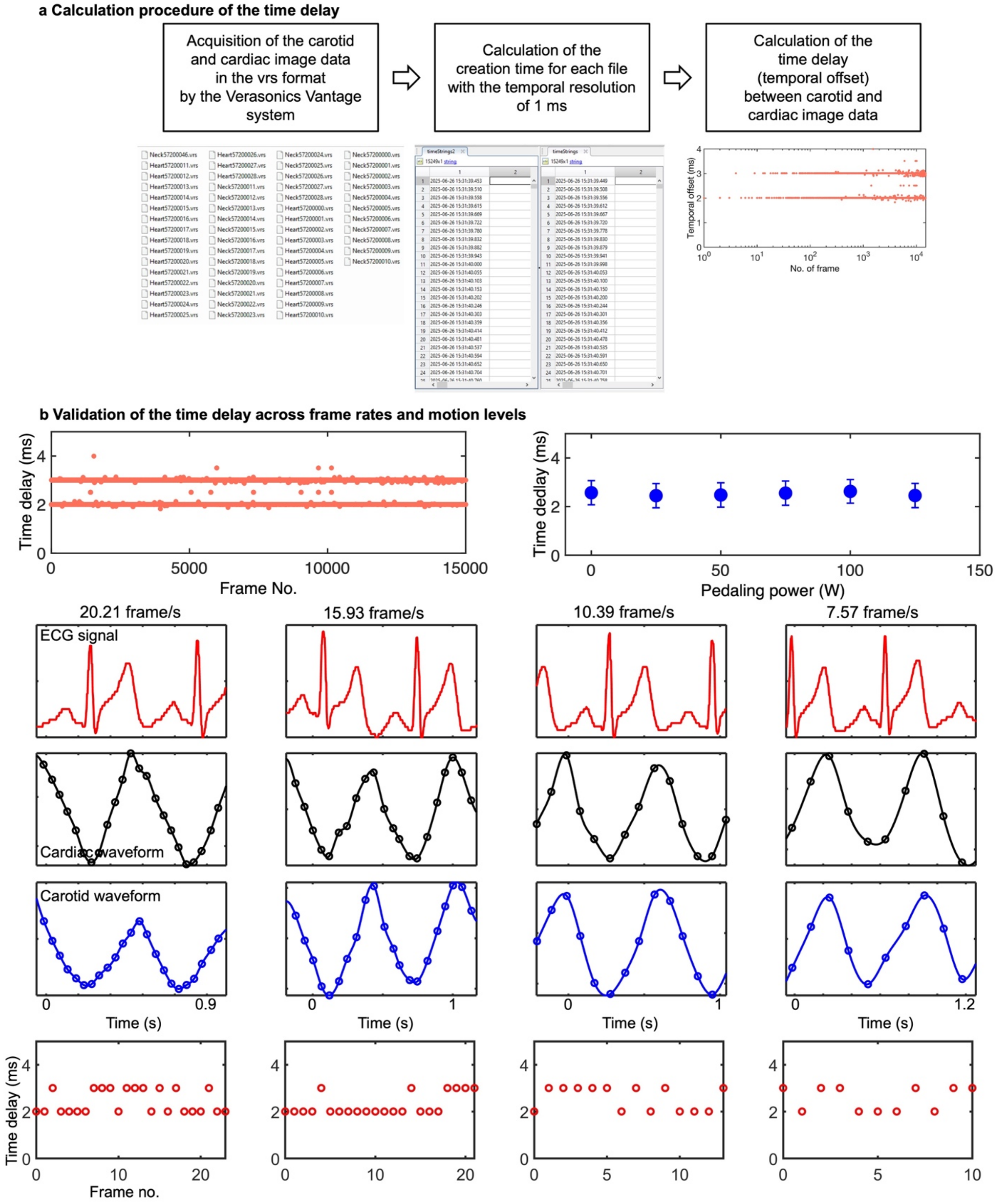

Fig. S17.

Calculation and validation of the time delay. **a**, Calculation procedure of the time delay. First, carotid and cardiac image data were acquired in the vrs format using the Verasonics Vantage system. Second, the creation time of each file was extracted with a custom program at a temporal resolution of 1 ms. Finally, the temporal offset between adjacent carotid and cardiac image file

was calculated. This method determines the time delay directly from the file creation timestamps, eliminating the need for an additional timing reference. **b**, Validation of the time delay across frame rates and motion levels. ECG waveforms were measured synchronously with ultrasound data across all acquisition patches and processed along a shared time axis. Across different imaging frame rates, the ECG, cardiac, and carotid waveforms were synchronously captured, revealing clear multi-phase variations. However, at lower frame rates, the cardiac and carotid waveforms rely more heavily on curve fitting to resolve temporal details. Time delays were calculated following the procedure described in **a** and showed similar values across different frame rate. Furthermore, these delays appear independent of motion amplitude (fig. S17b middle right panel), as the creation times of the cardiac and carotid data files are mainly determined by the hardware specifications of the host computer (current setup: Intel Xeon W-2255 CPU @ 3.70 GHz, 32 GB RAM, 64-bit OS). The use of a higher-performance computer is anticipated to reduce the time delay.

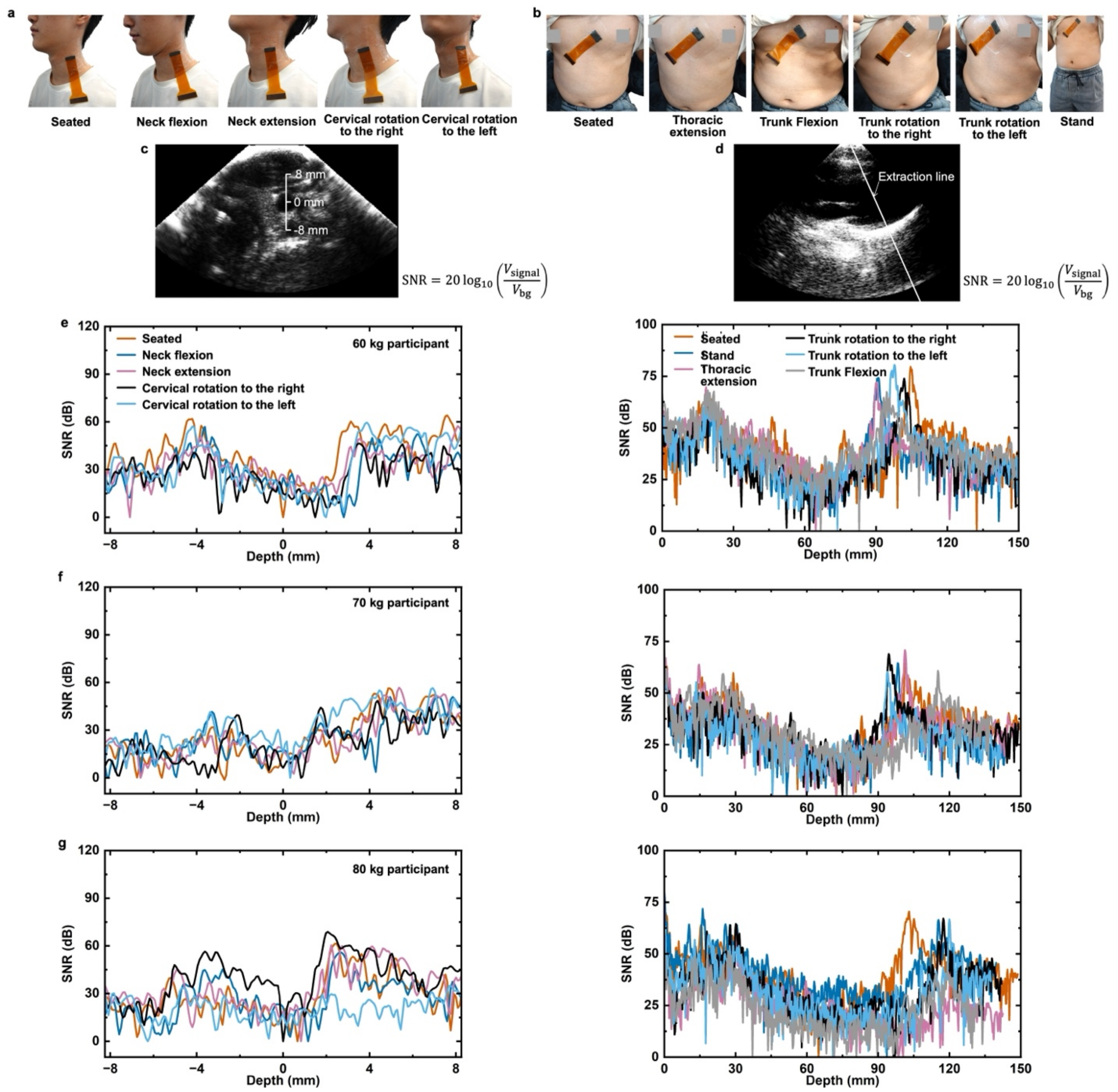

Fig. S18.

Imaging SNR vs. posture across participants. **a**, Carotid imaging postures: seated (baseline), neck flexion, neck extension, and left/right cervical rotation. **b**, Cardiac imaging postures: seated (baseline), thoracic extension, trunk flexion, left/right trunk rotation, and standing. **c-d**, Schematics illustrate the SNR calculation for carotid and cardiac images, defined as the logarithmic ratio of the target region brightness (V_{signal}) to the background brightness (V_{bg}), with the background selected as the lowest brightness point in the target region. The calculated SNR across participants with the body weight of **e**, 60 kg, **f**, 70 kg, and **g**, 80 kg. Results reveal two primary influences. First, participant weight: lighter participants generally achieved higher imaging SNR, likely due to reduced acoustic attenuation by fat tissue. Second, body postures: cervical rotation to the left or right (for carotid imaging), and trunk flexion (for cardiac imaging) consistently resulted in low SNR values across weight groups; other postures generally maintained SNR levels comparable to

the seated baseline. These results demonstrate that imaging SNR is influenced by participant weight and is degraded by cervical rotation and trunk flexion postures.

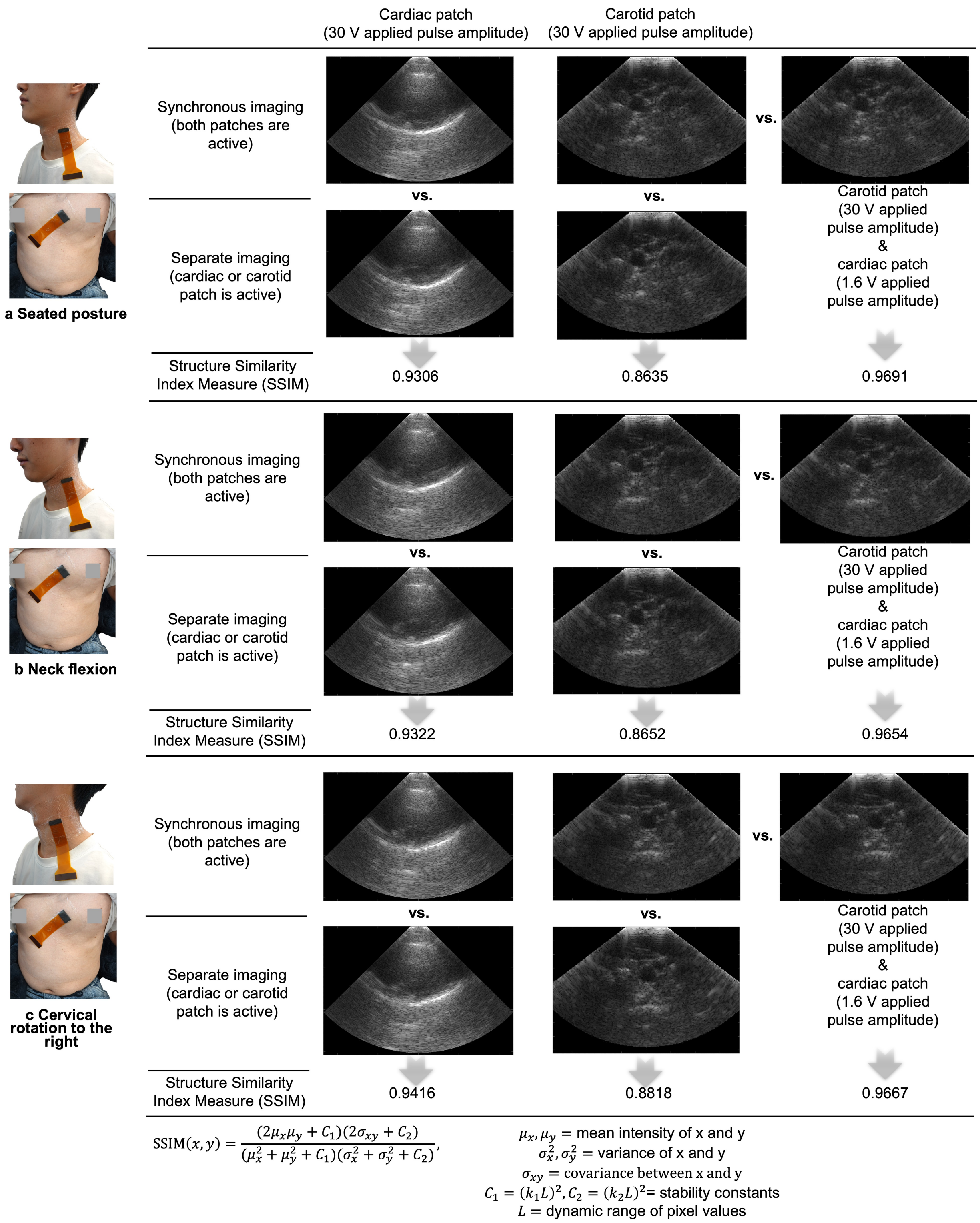

Fig. S19.

Crosstalk in the dual-patch system under different carotid postures. Crosstalk was evaluated under **a**, seated, **b**, neck-flexed, and **c**, cervical-rotation postures by acquiring three image types per posture: synchronous imaging of both patches at the applied pulse amplitude of 30 V, separate

imaging of each patch at 30 V, and synchronous imaging with the cardiac patch reduced to 1.6 V. Comparisons between the first and second image types enables the assessment of crosstalk effects introduced by synchronous operation. Comparisons between the first and third image types enables the evaluation of the crosstalk effects introduced by changing the patch's transmit pulse voltage amplitude. Using the Structural Similarity Index Measure (SSIM) for quantitative comparison, cardiac images showed high similarity (SSIM > 0.93) across image types, with minor variations attributable to crosstalk and inter-scan anatomical motion. Carotid images exhibited a slightly lower similarity (SSIM > 0.86), likely attributable to inter-scan anatomical motion. Given that carotid imaging captures detailed structural information, even minor muscle movement can alter the acquired ultrasound image. The conclusion that this variance stems from motion, rather than electrical crosstalk, is reinforced by the measured near-unity SSIM between the first and third imaging setups, which indicates minimal voltage-induced interference. These collective results demonstrate that crosstalk is limited when both transducer arrays are active.

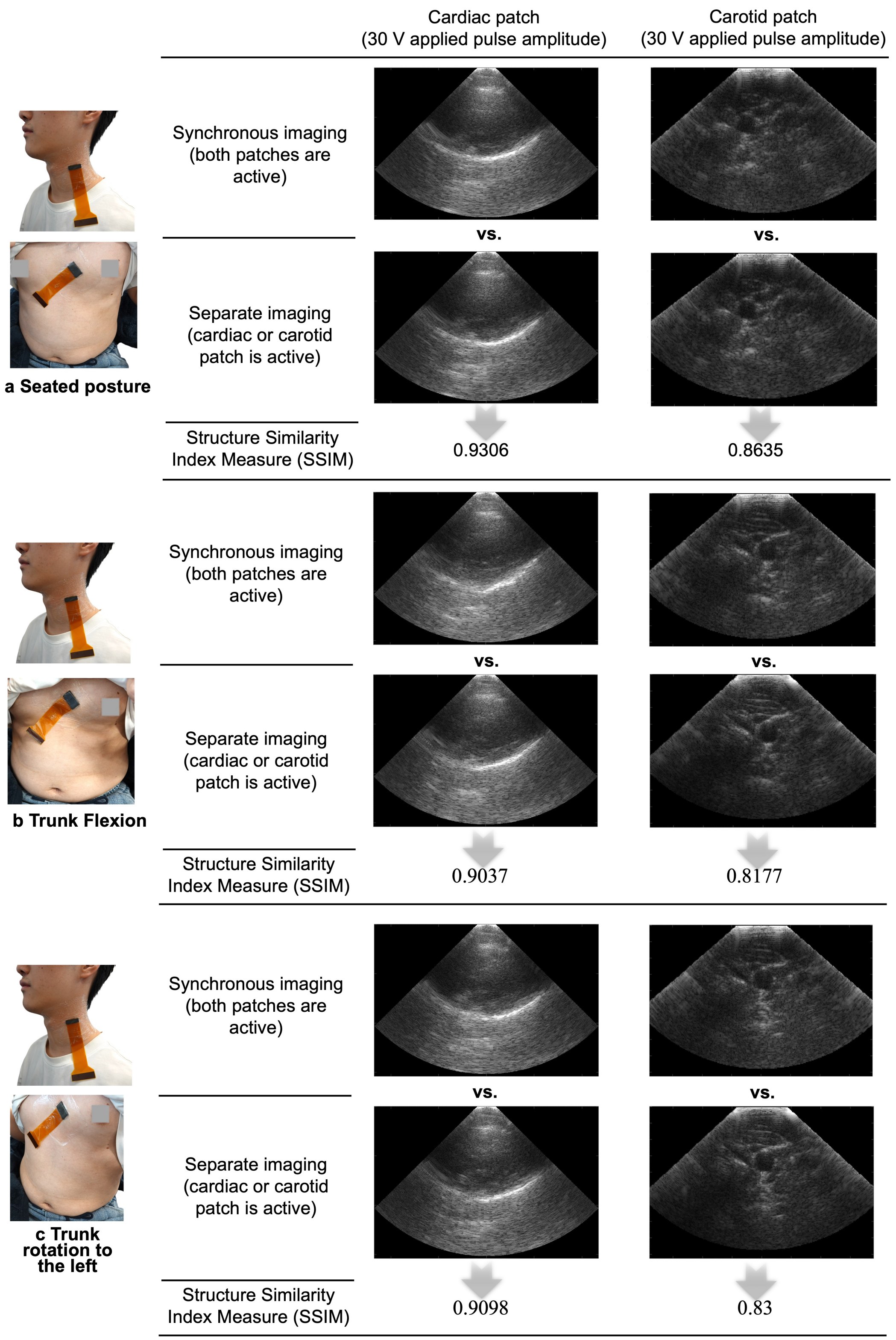

Fig. S20.

Crosstalk in the dual-patch system under different cardiac postures. Crosstalk was evaluated under **a**, seated, **b**, trunk flexion, and **c**, trunk rotation postures by acquiring two image types per posture:

synchronous imaging of both patches at the applied pulse amplitude of 30 V, and separate imaging of each patch at 30 V. Comparison between the image types was evaluated by SSIM. Cardiac images maintained consistently high similarity (SSIM > 0.90) across all postures, whereas carotid images exhibited moderately lower similarity (SSIM > 0.81). This residual dissimilarity, primarily attributed to anatomical motion between scans, further supports the conclusion that crosstalk between the active arrays is limited.

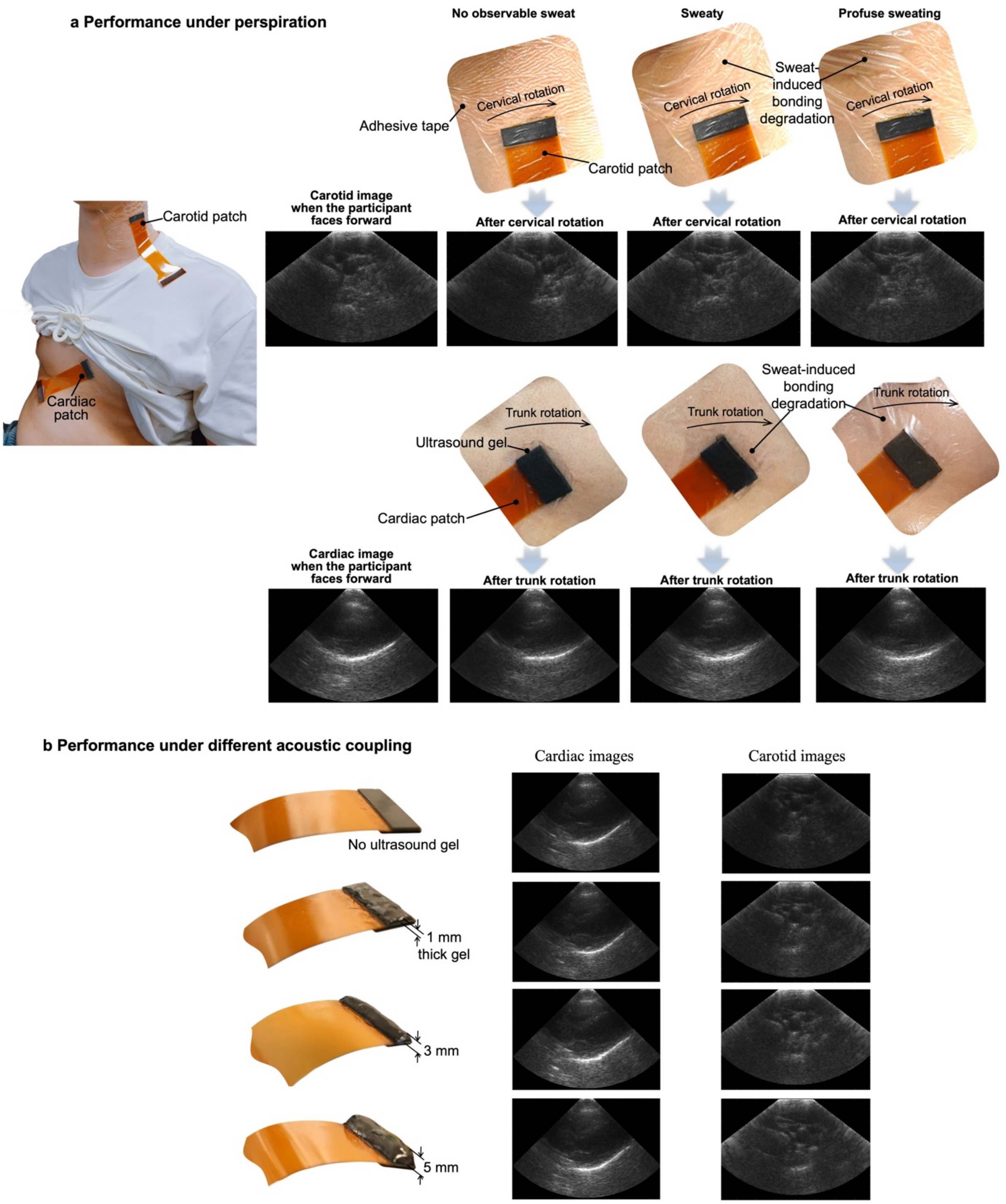

Fig. S21.

Working performance of the imaging system under **a**, perspiration and **b**, varying acoustic coupling conditions. In **a**, the participant performed continuous bicycle exercise to induce varying levels of sweating. Ultrasound images were acquired at baseline (no sweat), under moderate sweat, and under profuse sweat conditions. Without sweat, the medical-grade adhesive maintained secure skin contact. However, after continuous cycling (~20 and 30 minutes at 50 W, respectively),

moderate to profuse sweating caused slight adhesive loosening and sweat accumulation, degrading the bond. This led to minor patch sliding during cervical rotation, displacing the carotid artery in the image plane. Nonetheless, the wide field-of-view of the phased-array imaging strategy ensured continuous artery capture, allowing unimpeded diameter analysis and blood pressure calculation. This demonstrates the system's robust performance despite sweat-induced motion. The cardiac ultrasound patch exhibited similar behavior. In **b**, the ultrasound patch performed adequately without coupling gel, indicating that the acoustic matching layer of the patch facilitates effective ultrasound transmission through direct skin contact. However, different body postures inevitably introduced small air gaps, degrading image quality. Therefore, ultrasound gel was used for all in vivo measurements. Imaging performance remained consistent across different gel thicknesses, demonstrating robustness under varying coupling conditions.

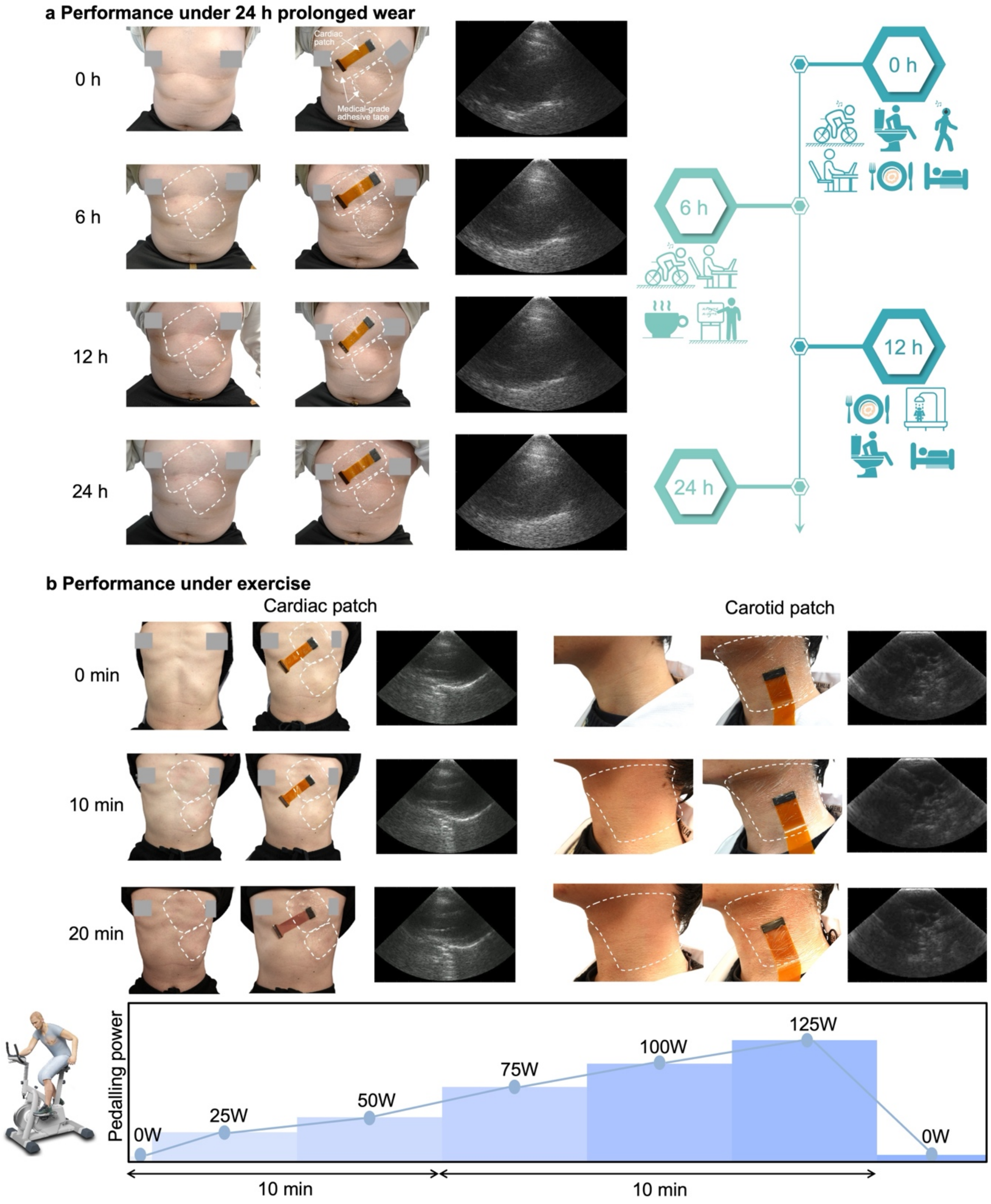

Fig. S22.

Working performance of the imaging system under **a**, 24 h prolonged wear and **b**, intensive exercise. In **a**, the participant wore the cardiac ultrasound patch continuously for 24 hours. Ultrasound images were captured in situ at 6, 12, and 24-hour intervals. Throughout this period, the participant engaged in a range of daily activities, including bicycling, walking, working, sleeping, drinking, and showering. The results showed no significant degradation in image quality

and no adverse symptoms, such as skin irritation, demonstrating the system's robust and safe performance during extended use. In **b**, the participant followed a controlled bicycle exercise protocol. Photographs and ultrasound images were acquired at 10-minute intervals. Similarly, no substantial decline in imaging quality or adverse symptoms (e.g., skin allergies) were observed, confirming the system's robust and safe performance under conditions of intense physical exertion. The illustration of the pedaling figure at the bottom left was created using C4D and Photoshop software. The illustrations in the upright panel were created using PowerPoint.

a Temperature measurements

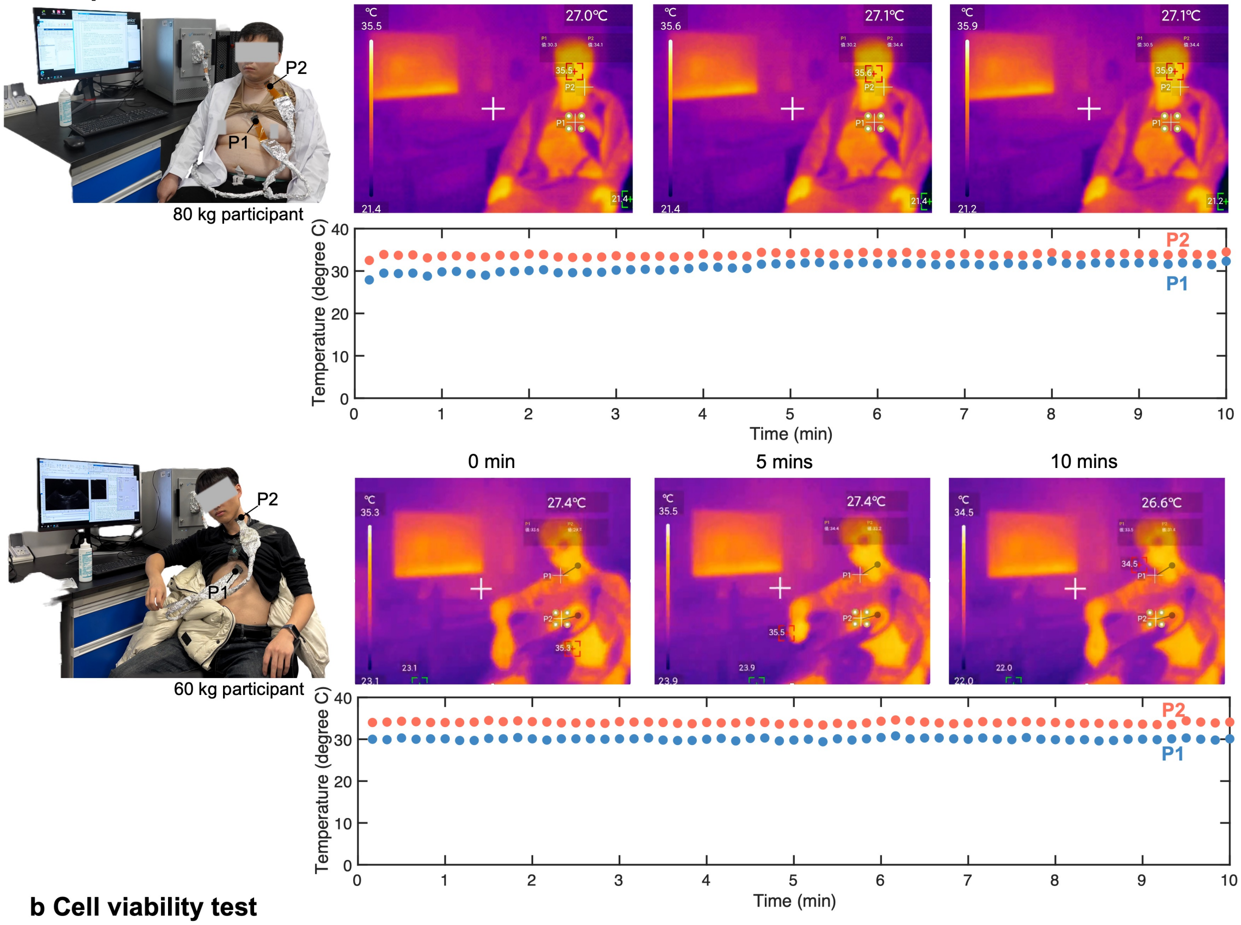

b Cell viability test

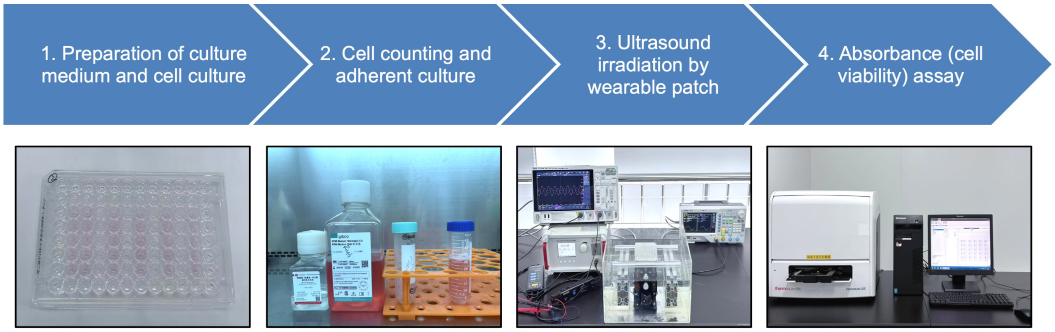

	Absorbance (cell viability) assay for five repeats					Mean	Std.
	No. 1	No. 2	No. 3	No. 4	No. 5		
Without ultrasound	1.1419	1.1569	1.0947	1.1291	0.9962	**1.10376**	**0.06436**
10 mins 3 MHz ultrasound	1.1495	1.1242	1.145	1.0813	0.9939	**1.09878**	**0.06454**
10 mins 4.5 MHz ultrasound	1.0096	1.1828	1.1918	1.1418	1.1604	**1.13728**	**0.07400**

Fig. S23.

Acoustic safety tests during prolonged imaging, validated by **a**, temperature measurement and **b**, cell viability test. For temperature measurements, two participants (body weights 60 kg and 80 kg) were recruited. Ultrasound patches were activated for approximately 10 minutes. A thermal infrared camera (NF-586s, China) was used for continuous temperature monitoring during patch activation. Results demonstrated stable temperatures with no observable increase, confirming the thermal safety of the imaging procedure. Acoustic exposure safety was further assessed using cell viability. PC-9 cancer cell was selected for the test. Ultrasound exposure parameters matched the imaging setup. Absorbance was used as the index to evaluate cell viability. The cardiac and carotid patches were positioned 5 cm and 1 cm beneath the petri dish, respectively. The experiment consisted of five separate tests per exposure condition. Results indicated no statistically significant difference in absorbance between cells with and without ultrasound exposure, confirming the acoustic safety of the patches for prolonged use.

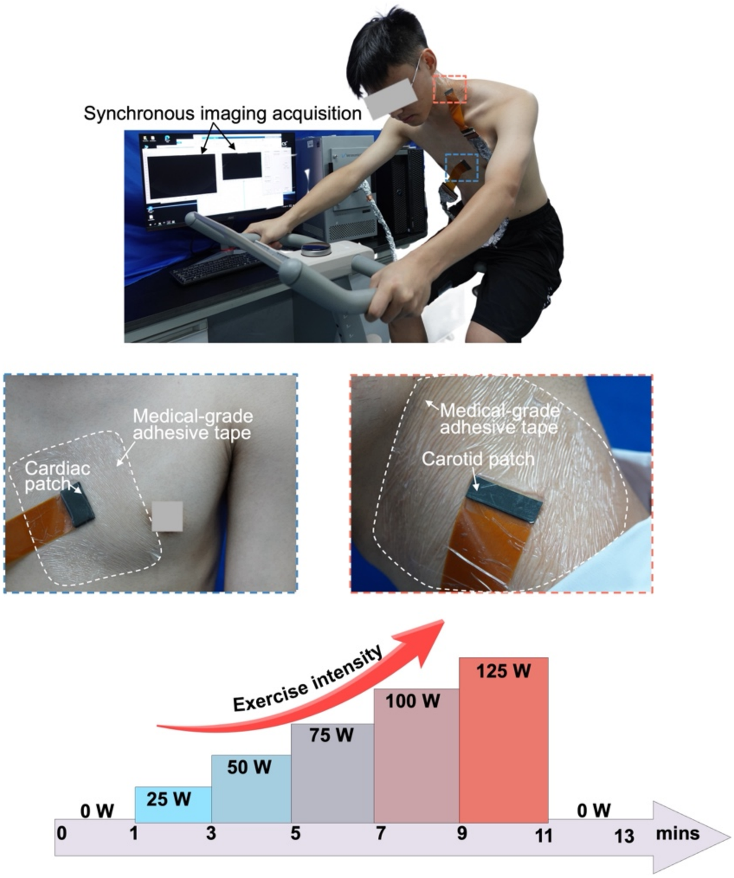

Fig. S24.

Experimental setup for synchronous cardiac-carotid data collection. The participant was instructed to ride a bicycle following the standardized exercise protocol (table S1), while cardiac and carotid ultrasound patches were securely adhered to the chest and neck for continuous, real-time imaging. To enable synchronized acquisition, custom imaging algorithms were developed. Exercise intensity was gradually increased from 0 W by raising the pedal resistance. Participants maintained each power output for approximately two minutes and retained the right to discontinue the protocol at any point due to excessive fatigue or discomfort.

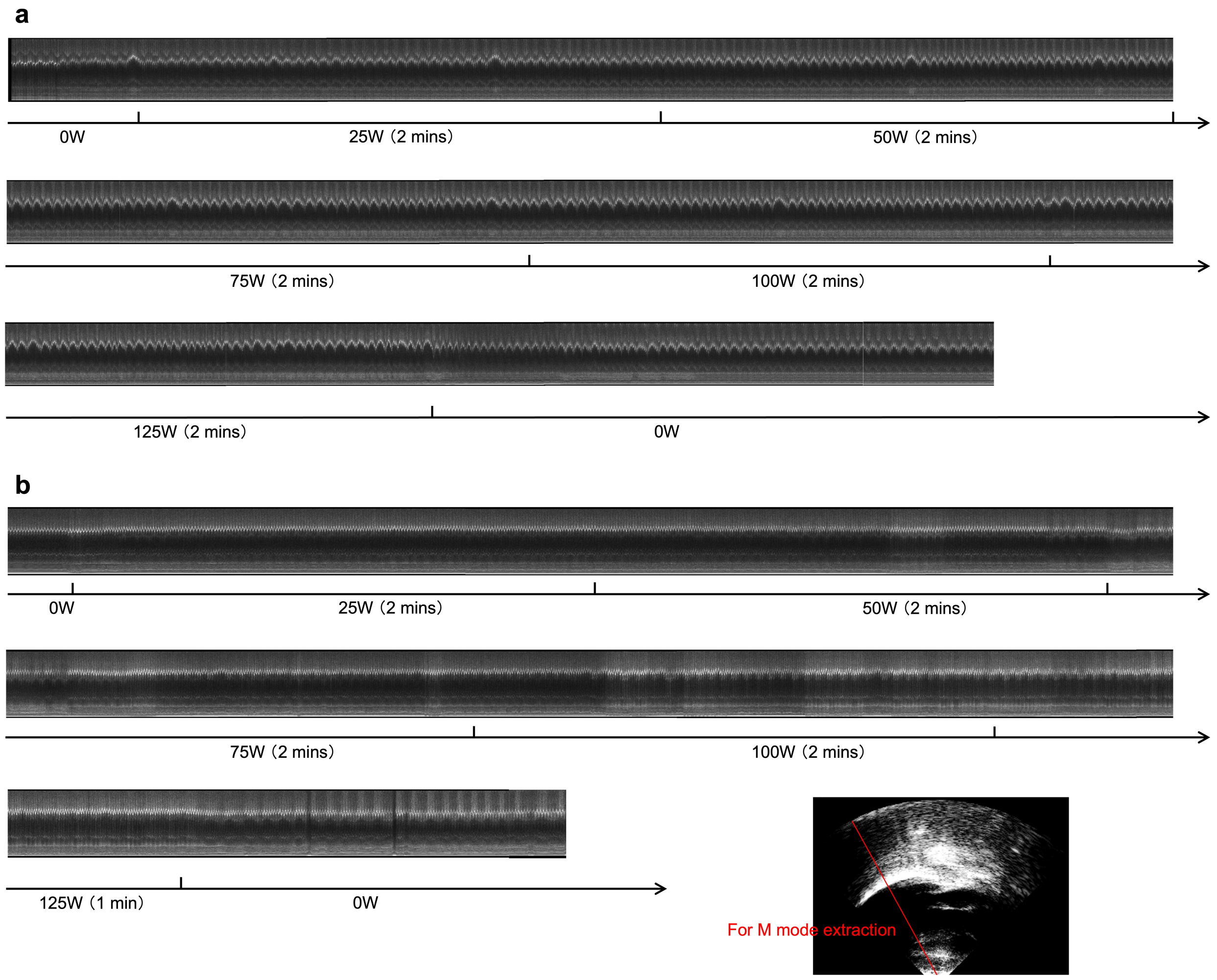

Fig. S25.

Full sequence of cardiac M-mode ultrasound images plotted in Fig. 3. **a** and **b** represent images extracted from two participants with the weight of 60 kg and 80 kg, respectively. As participants could voluntarily stop the test at any time based on their comfort, the participant in **b** cycled at 125 W but only completed half of the target duration.

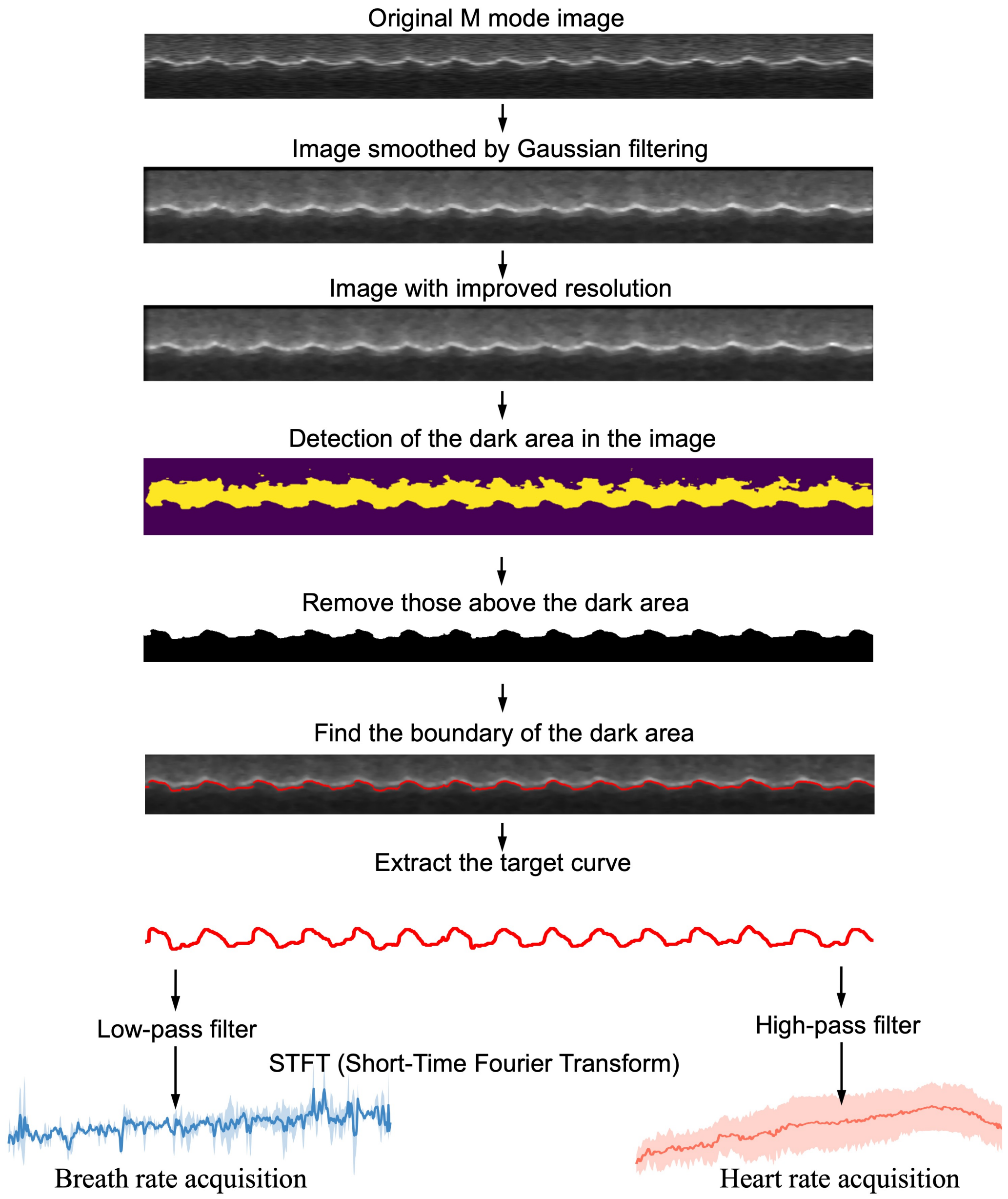

Fig. S26.

Processing pipeline for obtaining breath and heart rates from cardiac-carotid M-mode images. It is noted that the breath rate is lower than (~1/5) the heart/pulse rates. Therefore, low-pass filter can be used to extract the breath rate signals.

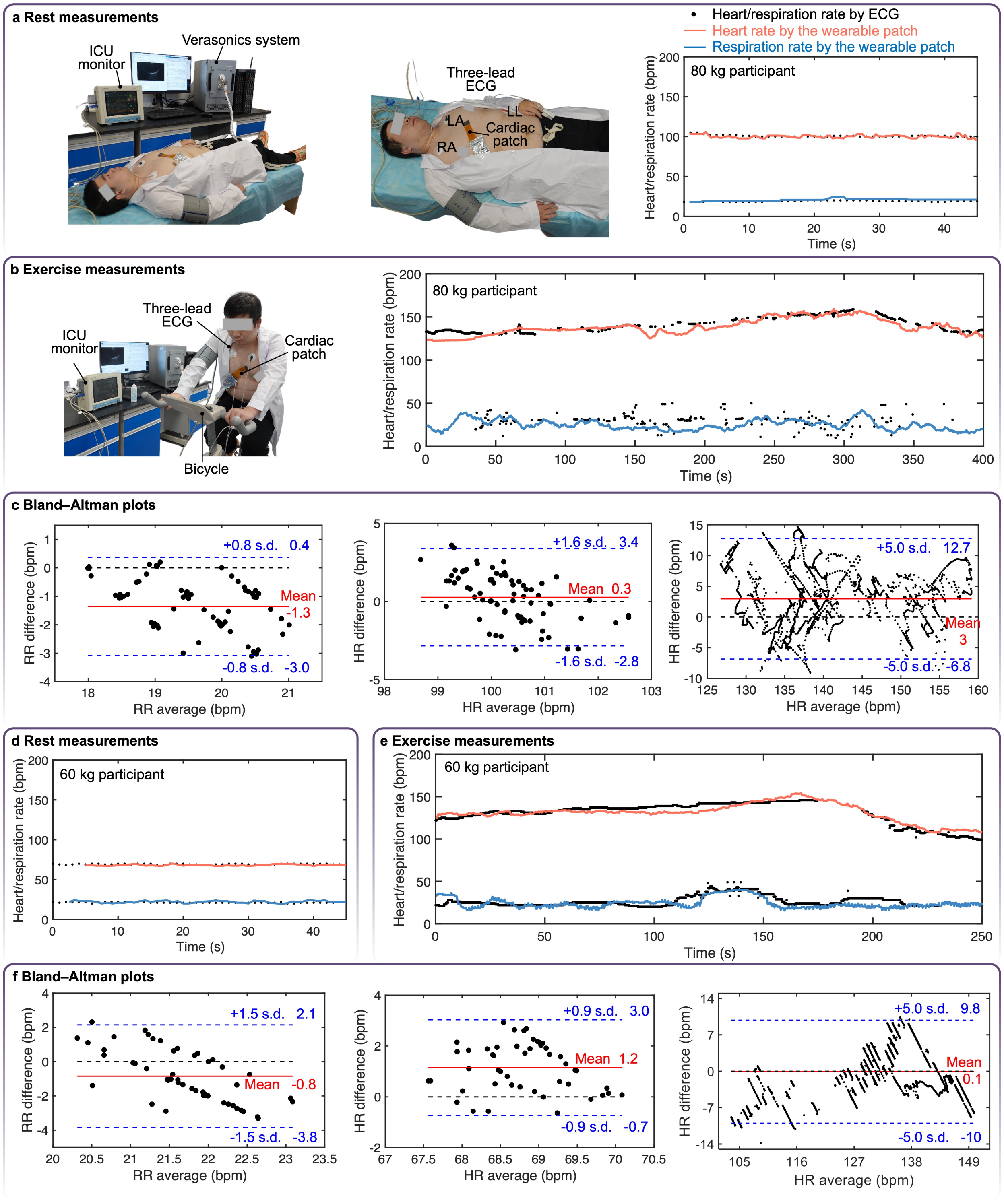

Fig. S27.

Monitoring performance comparison between the commercial ECG (MEC 1000 system) and the wearable cardiac patch. Two participants with body weights of 60 kg (**a-c**) and 80 kg (**d-f**) were recruited. Each participant was monitored under both resting and exercise conditions using both devices simultaneously to capture heart rate (HR) and respiration rate (RR). While the commercial

ECG derives HR directly from cardiac electrical signals and estimates RR from respiratory modulation of the ECG waveform, the wearable patch extracts both HR and RR via Short-Time Fourier Transform (STFT) analysis of the M-mode ultrasound images. For the resting condition, HR and RR measurements from the wearable patch showed strong agreement with the commercial ECG for both participants. Bland-Altman analysis for the resting state indicated a mean bias of approximately -0.3 bpm for HR and +1.3 bpm for RR. The limits of agreement (LOA) were 6.2 bpm for HR and 3.4 bpm for RR, with scatter points indicating relatively consistent disagreement across the measurement range. During exercise, HR trends were similar for both devices and participants. However, the ECG-derived RR for the 80 kg participant exhibited greater variability than for the 60 kg participant, a discrepancy potentially attributable to physiological differences. Bland-Altman analysis during exercise showed average HR difference of 3 bpm and 0.1 bpm for the two participants, respectively. Collectively, these results demonstrate the reliable performance of the developed wearable cardiac patch in extracting both HR and RR across different physiological states.

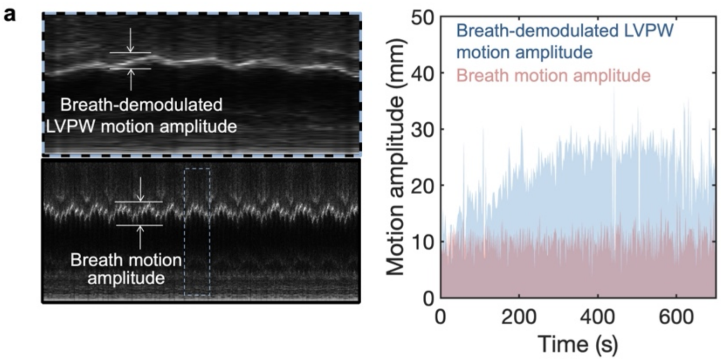

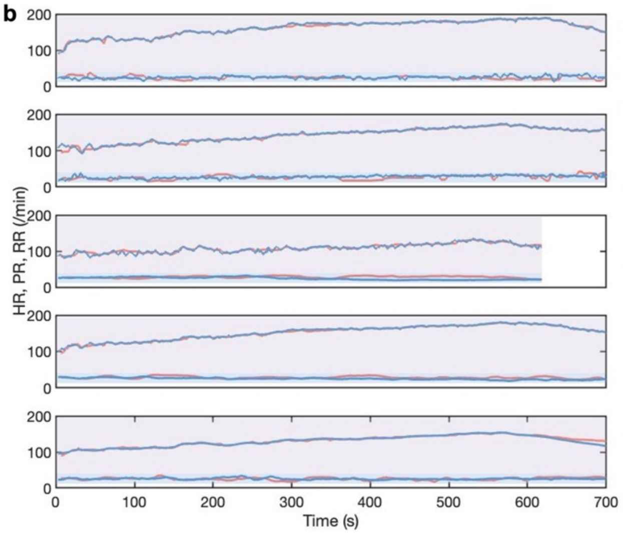

		Pearson correlation coefficient	95% confidence interval	Mean absolute difference (bpm)	Standard deviation
Participant 1	HR vs. PR	0.9935	[0.9930, 0.9941]	1.56	2.42
	RR vs. RR	<0.5		3.87	5.56
Participant 2	HR vs. PR	0.9851	[0.9837, 0.9865]	1.98	3.51
	RR vs. RR	<0.5		4.35	5.53
Participant 3	HR vs. PR	0.9500	[0.9466, 0.9532]	2.59	3.69
	RR vs. RR	<0.5		5.63	5.35
Participant 4	HR vs. PR	0.9953	[0.9950, 0.9956]	1.36	1.97
	RR vs. RR	<0.5		4.06	3.29
Participant 5	HR vs. PR	0.9882	[0.9874, 0.9892]	1.55	2.32
	RR vs. RR	<0.5		3.16	3.90

Fig. S28.

a, Cardiac and respiratory motion amplitude. **b**, HR/PR/RR curves of five healthy participants and the correlation metrics. The metrics include concordance, Pearson correlation coefficient with confidence interval, mean absolute difference, and standard deviation. The Pearson correlation coefficients and confidence intervals for HR and PR are consistently high, and the mean absolute difference for these measure ranges from 1.36 to 2.59 bpm, indicating reliable extraction of HR and PR from cardiac and carotid images. In contrast, RR extraction is more variable. It is considered more reliable with cardiac images, where the left ventricular posterior wall (LVPW) motion waveform couples with obvious, high-amplitude breathing motion, but the carotid pulsation amplitude is relatively small to resolve detailed respiration motion.

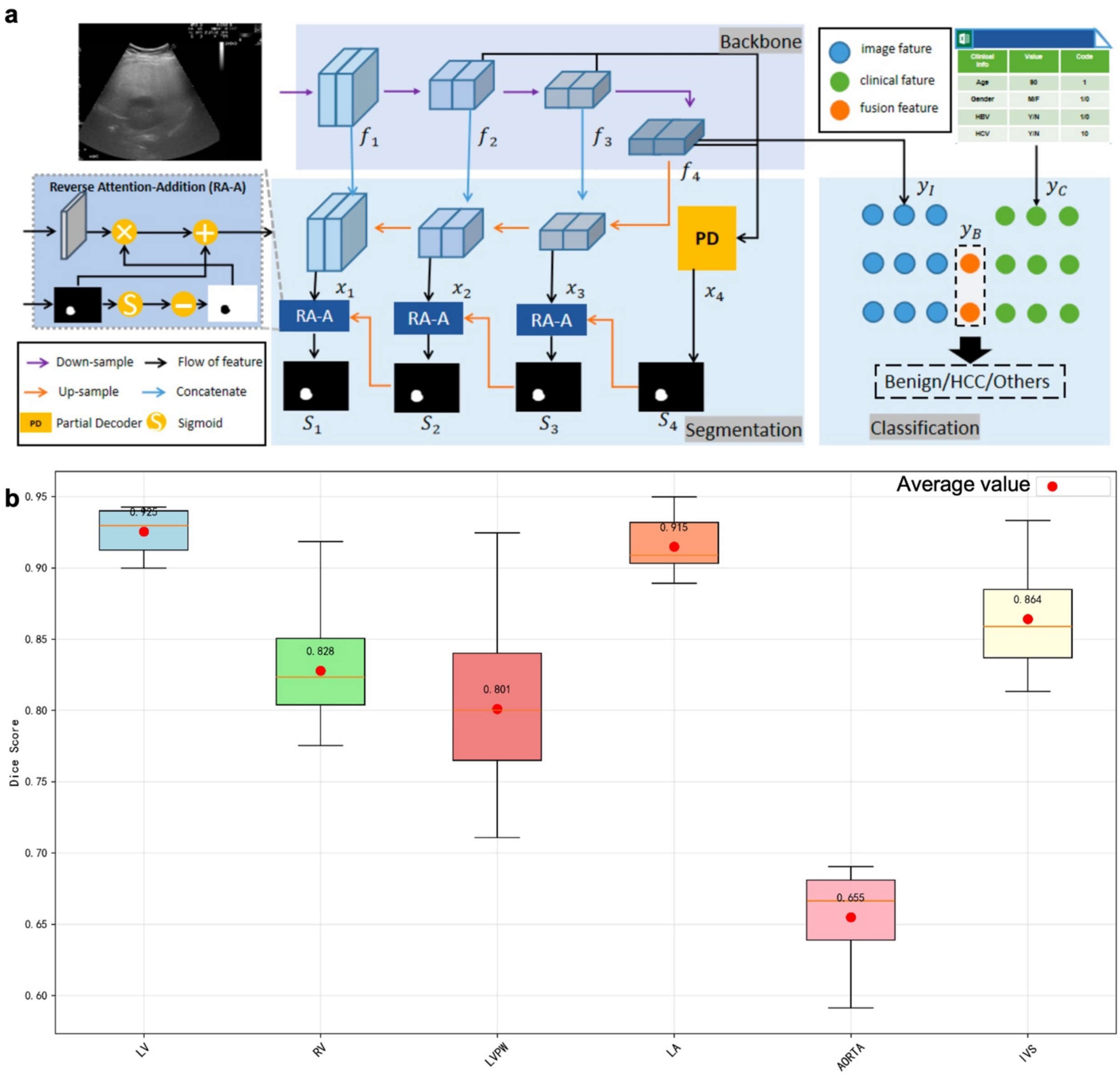

Fig. S29.

Cardiac AI model structure and performance. **a**, The main structure of the MTANet (*45*) model for cardiac wearable ultrasound image segmentation. MTANet employed a reverse addition attention module alongside a parallel partial decoder within the basic UNet's decoder to enhance high-resolution feature extraction for the segmentation branch. Additionally, it integrated attention bottleneck modules in the fully connected layers to fuse imaging features with clinical features for the classification branch. In this study, the MTANet model was initially trained on clinical cardiac ultrasound images and subsequently adapted for segmenting cardiac images acquired from wearable ultrasound patches, detailed description of the cardiac image processing has been added to Supplementary Text. **b**, The Dice scores achieved by the model for different heart segments.

a

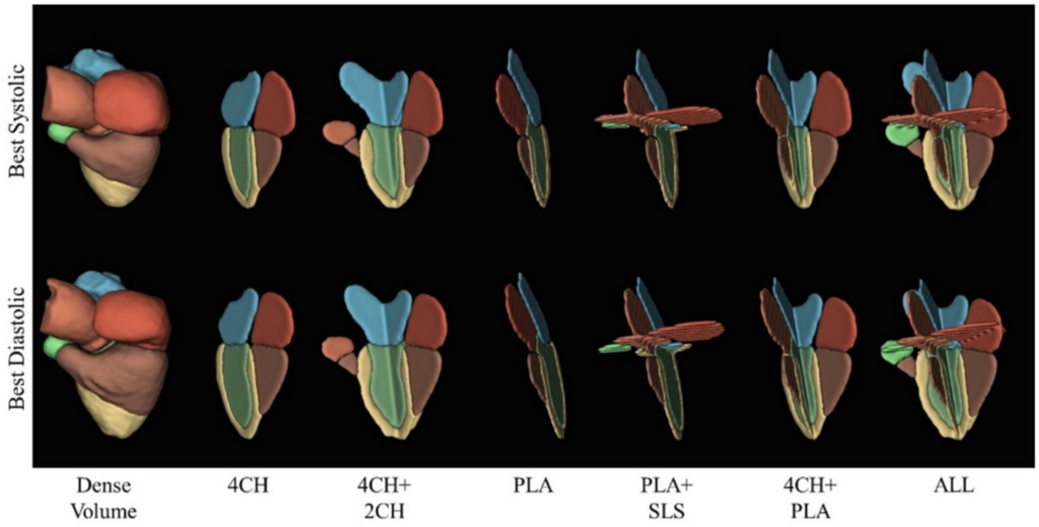

b

	LV	LVM	RV	LA	RA	AOR	PUL
4CH	0.922 ± 0.028	0.862 ± 0.062	0.899 ± 0.046	0.908 ± 0.042	0.901 ± 0.048	0.793 ± 0.096	0.621 ± 0.182
4CH+ 2CH	0.959 ± 0.013	0.935 ± 0.035	0.926 ± 0.040	0.948 ± 0.028	0.914 ± 0.044	0.845 ± 0.071	0.809 ± 0.150
PLA	0.926 ± 0.039	0.868 ± 0.035	0.899 ± 0.053	0.905 ± 0.048	0.842 ± 0.073	0.913 ± 0.057	0.735 ± 0.147
PLA+ PSA	0.942 ± 0.024	0.903 ± 0.042	0.918 ± 0.042	0.906 ± 0.042	0.863 ± 0.060	0.913 ± 0.053	0.779 ± 0.134
4CH+ PLA	0.951 ± 0.016	0.919 ± 0.036	0.936 ± 0.035	0.939 ± 0.030	0.918 ± 0.044	0.914 ± 0.052	0.745 ± 0.142
4CH+ PLA+*	0.948 ± 0.017	0.913 ± 0.039	0.926 ± 0.036	0.935 ± 0.031	0.904 ± 0.047	0.874 ± 0.084	0.689 ± 0.160
ALL	**0.966** ± 0.011	**0.945** ± 0.036	**0.951** ± 0.030	**0.958** ± 0.024	**0.932** ± 0.036	**0.925** ± 0.046	**0.855** ± 0.113
ALL+ MA	0.966 ± 0.011	0.944 ± 0.028	0.950 ± 0.029	0.958 ± 0.024	0.931 ± 0.036	0.925 ± 0.047	0.851 ± 0.121

c

	LV	LVM	RV	LA	RA	AOR	PUL
4CH	5.07 ± 1.53	5.09 ± 1.51	7.64 ± 4.77	6.67 ± 2.50	7.57 ± 2.76	9.04 ± 3.30	7.11 ± 3.89
4CH+ 2CH	3.64 ± 1.04	3.91 ± 1.45	6.52 ± 4.79	5.56 ± 4.85	7.20 ± 3.51	7.57 ± 3.54	5.09 ± 4.87
PLA	5.18 ± 1.53	5.30 ± 1.77	6.73 ± 2.26	7.58 ± 3.90	8.03 ± 2.54	6.24 ± 3.15	5.13 ± 2.38
PLA+ PSA	4.12 ± 1.28	4.67 ± 1.75	6.66 ± 3.43	7.07 ± 2.33	7.51 ± 1.99	6.24 ± 2.77	4.62 ± 2.06
4CH+ PLA	4.03 ± 1.30	4.09 ± 1.11	6.39 ± 3.89	5.28 ± 1.46	6.09 ± 1.75	5.84 ± 3.19	5.39 ± 4.06
4CH+ PLA+*	4.12 ± 1.13	4.33 ± 1.44	6.53 ± 3.10	5.97 ± 3.62	6.46 ± 2.52	6.30 ± 2.66	5.51 ± 2.22
ALL	**3.22** ± 1.02	**3.75** ± 1.72	**5.08** ± 2.56	4.51 ± 1.71	**5.60** ± 2.22	**5.22** ± 2.28	**3.83** ± 2.05
ALL+ MA	3.63 ± 3.46	4.20 ± 4.44	5.18 ± 2.63	**4.50** ± 1.41	6.17 ± 4.40	5.41 ± 2.24	4.29 ± 5.05

Fig. S30.

Demonstration of the cardiac reconstruction model accuracy**.** The accuracy was evaluated as Dice similarity coefficients under different combinations of acoustic windows, arrays, and levels of motion of the heart by our early study (*79*). **a**, Illustrations of the reconstructed dense and sparse volumes in the best diastolic and systolic frames. Reconstruction accuracy (reported as mean ± standard deviation) using the **b**, Dice Score and **c**, Hausdorff Distance. The asterisk (*) denotes testing on input volumes with patch position variations. The abbreviations are listed as follows: parasternal long axis (PLA), parasternal short axis (PSA), two chamber (2CH) and four chamber (4CH) apical views, left ventricle (LV), left ventricle myocardium (LVM), right ventricle (RV), left atrium (LA), right atrium (RA), aortic root (AOR), pulmonary artery (PUL), left pulmonary veins (LPV), right pulmonary veins (RPV) and left atrial appendage (LAA). The ultrasound patch was attached under different standard views or their combinations (PLA, PSA, 2CH, 4CH). The nnU-Net model was then used to reconstruct the 3D shape of the heart with the acquired images. The reconstruction accuracy by the ultrasound patch images was compared to the ground truth reconstructed by Coronary Computed Tomography Angiography (CCTA). Results show that the most cost-effective solution was using a single linear array in the parasternal long-axis (PLA) view, as the case for this study, which presents a Dice Score of 0.87 for all seven labels, particularly effective for aortic reconstruction.

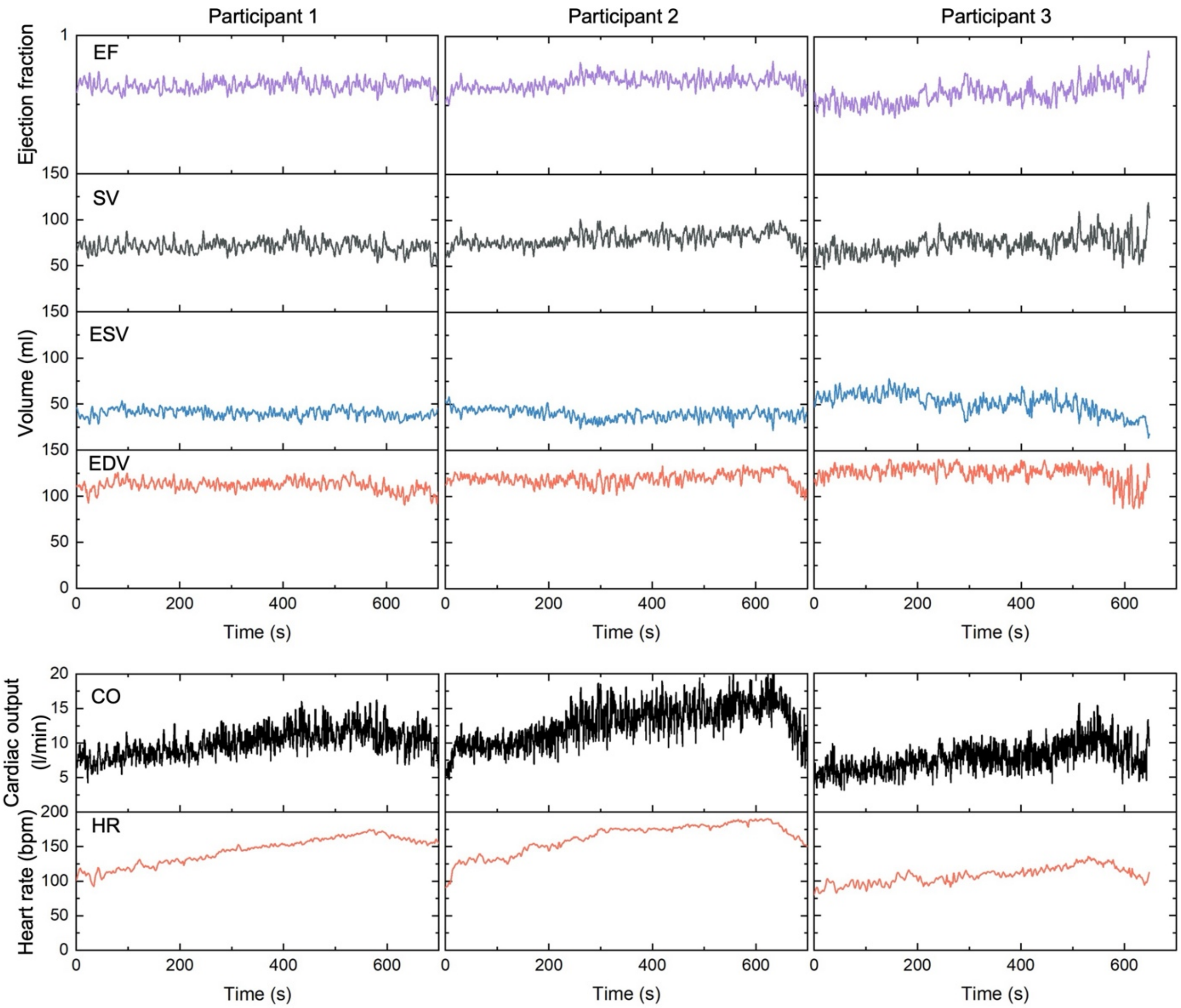

Fig. S31.

Variations of the cardiac metrics across participants. Depending on the physical fitness of each participant, slightly different variations were observed. However, in general, SV, EF, and CO all tended to increase with exercise intensity before declining during recovery. These trends were consistent across participants. Results from participant 1 are shown in Fig. 4.

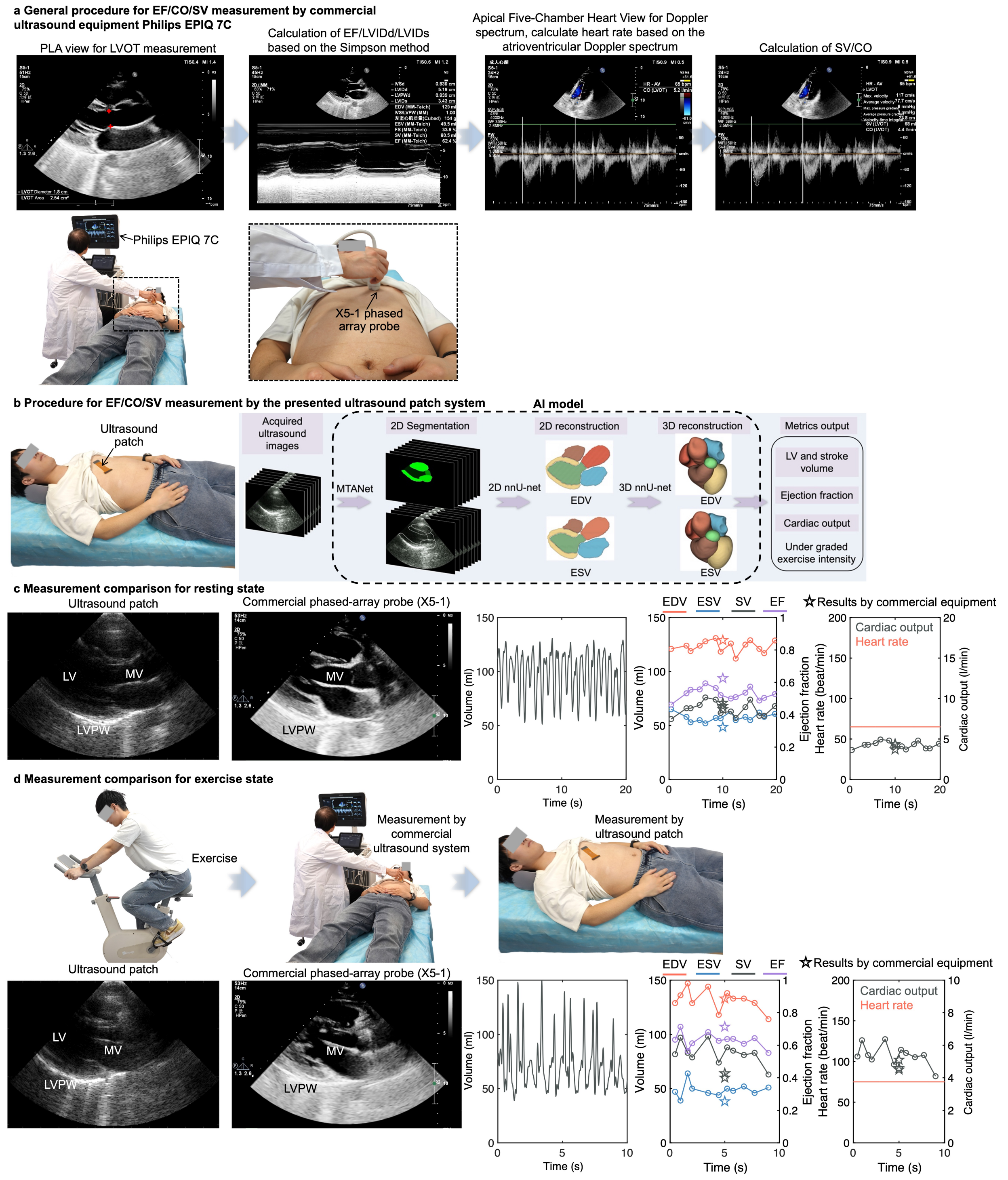

Fig. S32.

Performance validation of the cardiac ultrasound patch against commercial ultrasound equipment (Philips, EPIQ 7c). **a**, General procedure for EF/CO/SV measurement by commercial ultrasound equipment. Measurement of EF, CO, and SV was performed according to a standardized ultrasound protocol. The procedure commenced with acquisition of a PLA view for assessment of the left ventricular outflow tract (LVOT). Ejection fraction was subsequently calculated via the

Simpson (biplane) method. Heart rate was determined from the atrioventricular Doppler spectrum obtained in the apical five-chamber view. Stroke volume and cardiac output were then derived from these core measurements. **b**, Procedure for EF/CO/SV measurement by the presented ultrasound patch system. Following image acquisition in the PLA view, the data is processed by a trained AI model to generate the hemodynamic metrics of EF/CO/SV. **c**, Measurement comparison between the developed cardiac patch and commercial ultrasound equipment for resting state. Ultrasound images captured by the cardiac patch (left) and commercial equipment (second to the left), and the quantitative comparison of the measured EF/CO/SV metrics (right). Both ultrasound images demonstrate strong similarity, successfully capturing the key cardiac anatomical structures. Metrics by the commercial equipment were obtained via the standard clinical procedure (**a**). The standard clinical procedure yielded the following cardiac metrics: EDV = 129 ml, ESV = 48.5 ml, EF = 0.625, CO = 4.13 L/min, and SV = 68 ml. Measurements from the cardiac patch showed respective differences of -5.97 ± 5.29 ml, 9.28 ± 3.83 ml, -0.0956 ± 0.0385, 0.108 ± 0.443 L/min, and -2.75 ± 6.81 ml, with most differences falling within 5% of the clinical standard. **d**, Measurement comparison for exercise state. The participant completed a five-minute bicycle exercise protocol, after which the participant was in supine posture for image acquisition. Cardiac images were first captured using the commercial ultrasound equipment, followed by measurement with the cardiac patch. Both images reveal key anatomical structures. Representative images from the cardiac patch and the commercial system are shown, alongside a quantitative comparison of the derived cardiac metrics. Under exercise conditions, the standard clinical procedure produced the following measurements: EDV = 133 ml, ESV = 38.2 ml, EF = 0.713, CO = 4.73 L/min, and SV = 62.7 ml. The cardiac patch results showed respective differences of -1.22 ± 9.86 ml, -10.6 ± 6.2 ml, -0.0845 ± 0.0456, -0.658 ± 0.635 L/min, and 20.28 ± 9.77 ml, with most differences falling within 15% of the clinical standard. This larger variation is possibly attributed to the fact that measurements by the cardiac patch was taken around 1 min after the commercial equipment, which results in physiological changes. Quantitative cardiac metrics under both resting and post-exercise states demonstrate the reliable results from the cardiac patch. For clarity and completeness, the data shown in Fig. 4H is also plotted in fig. S32 here.

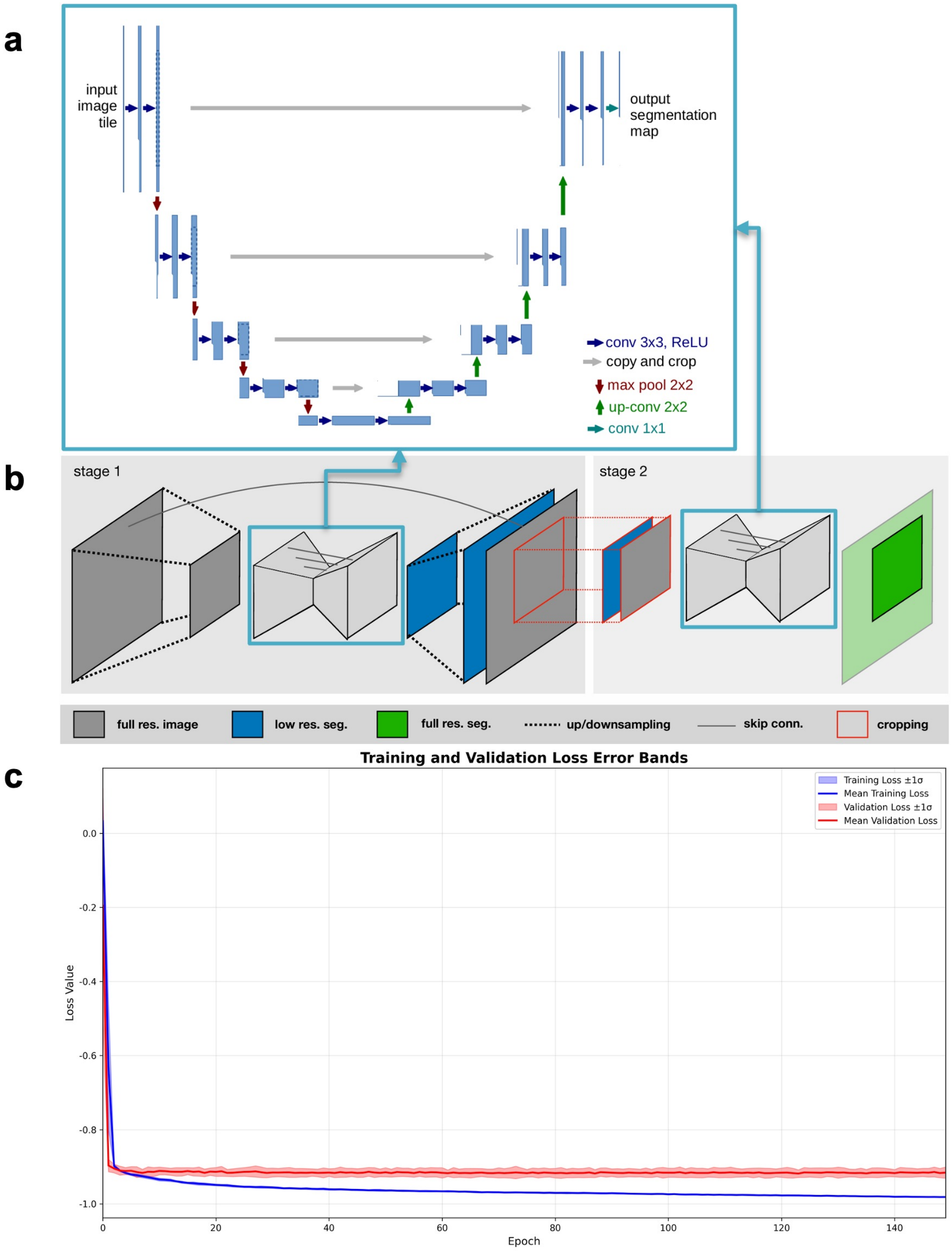

Fig. S33.

The nnU-Net (*47*) model for carotid wearable ultrasound image segmentation. **a**, U-Net model architecture overview. **b**, Illustration of nnU-Net cascade segmentation stages. **c**, Training and validating losses of the network training progress. Detailed description of the model is shown in Supplementary Text.

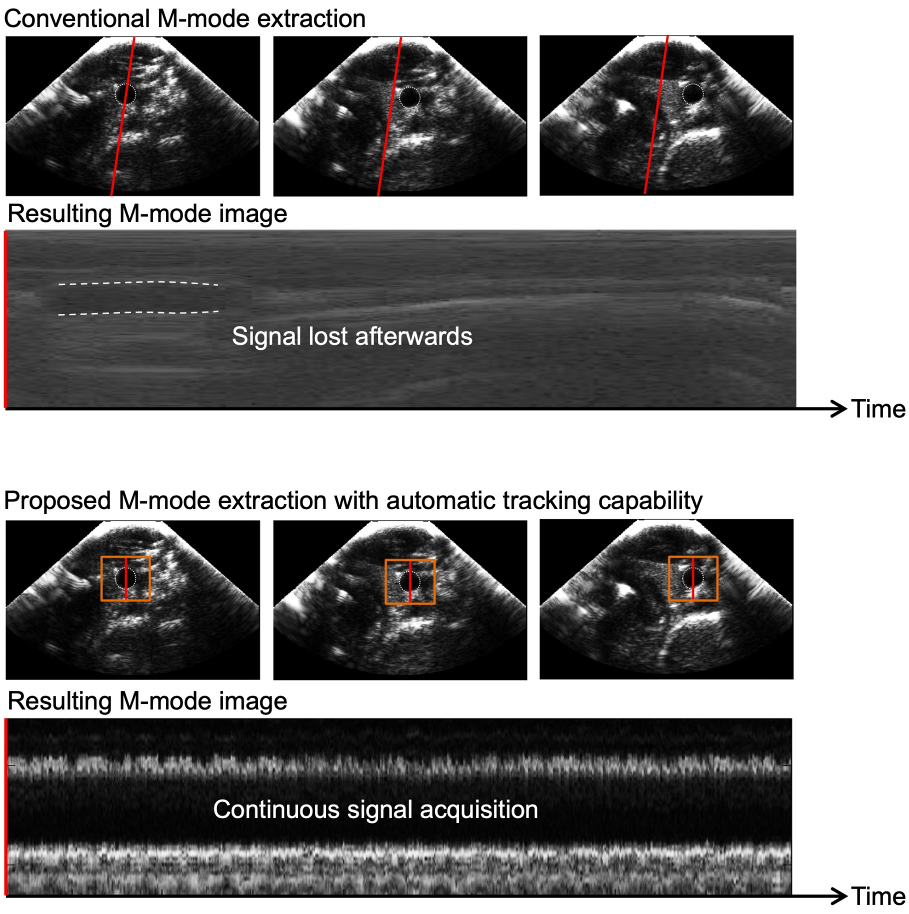

Fig. S34.

The proposed M-mode extraction method for carotid images. It enables automatic tracking of the carotid during extensive body movements. The conventional M-mode extraction method relies on a fixed line to capture image information across all frames. As a result, the M-mode image often loses signals when the carotid artery is displaced due to body movements. In contrast, the proposed method employs automatic carotid tracking, enabling the placement of a fixed-size window for precise and automated M-mode extraction.

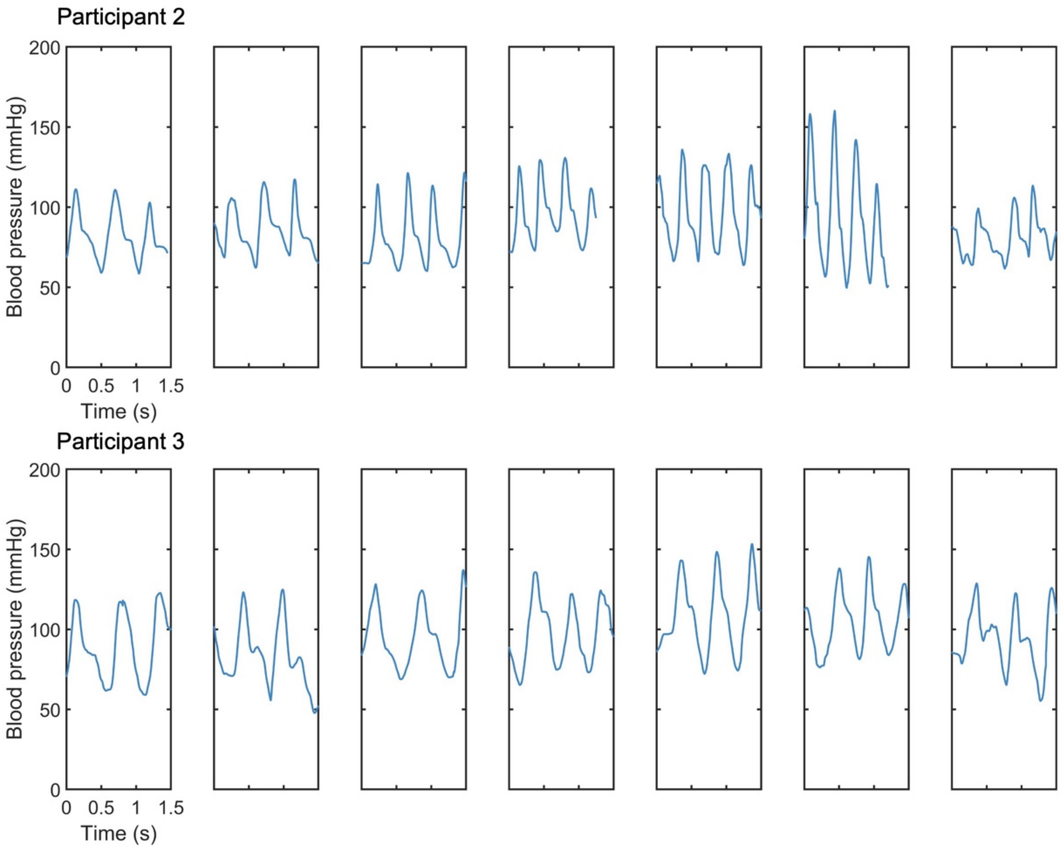

Fig. S35.

Blood pressure waveforms under graded exercise intensity.

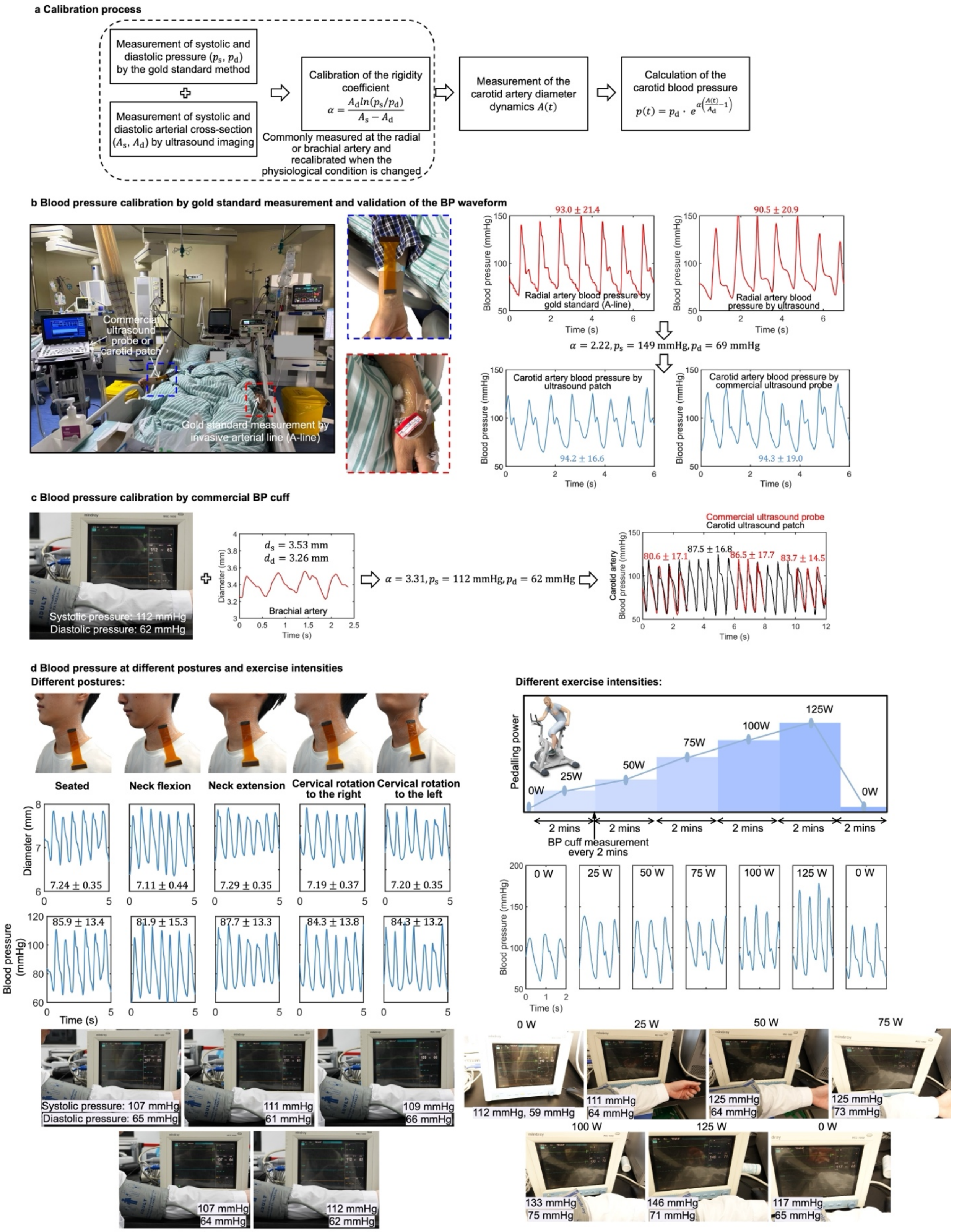

a Calibration process
Measurement of systolic and diastolic pressure (p_s, p_d) by the gold standard method
Measurement of systolic and diastolic arterial cross-section (A_s, A_d) by ultrasound imaging
Calibration of the rigidity coefficient $\alpha = \frac{A_d ln(p_s/p_d)}{A_s - A_d}$
Commonly measured at the radial or brachial artery and recalibrated when the physiological condition is changed
Measurement of the carotid artery diameter dynamics $A(t)$
Calculation of the carotid blood pressure $p(t) = p_d \cdot e^{\alpha\left(\frac{A(t)}{A_d}-1\right)}$
b Blood pressure calibration by gold standard measurement and validation of the BP waveform
Commercial ultrasound probe or carotid patch
Gold standard measurement by invasive arterial line (A-line)
Radial artery blood pressure by gold standard (A-line)
93.0 ± 21.4
Radial artery blood pressure by ultrasound
90.5 ± 20.9
$\alpha = 2.22, p_s = 149$ mmHg, $p_d = 69$ mmHg
Carotid artery blood pressure by ultrasound patch
94.2 ± 16.6
Carotid artery blood pressure by commercial ultrasound probe
94.3 ± 19.0
Blood pressure (mmHg)
Time (s)
c Blood pressure calibration by commercial BP cuff
Systolic pressure: 112 mmHg
Diastolic pressure: 62 mmHg
$d_s = 3.53$ mm
$d_d = 3.26$ mm
Brachial artery
Diameter (mm)
Time (s)
$\alpha = 3.31, p_s = 112$ mmHg, $p_d = 62$ mmHg
Commercial ultrasound probe
Carotid ultrasound patch
80.6 ± 17.1
87.5 ± 16.8
86.5 ± 17.7
83.7 ± 14.5
Carotid artery Blood pressure (mmHg)
d Blood pressure at different postures and exercise intensities
Different postures:
Seated
Neck flexion
Neck extension
Cervical rotation to the right
Cervical rotation to the left
Diameter (mm)
7.24 ± 0.35
7.11 ± 0.44
7.29 ± 0.35
7.19 ± 0.37
7.20 ± 0.35
Blood pressure (mmHg)
85.9 ± 13.4
81.9 ± 15.3
87.7 ± 13.3
84.3 ± 13.8
84.3 ± 13.2
Time (s)
Systolic pressure: 107 mmHg
Diastolic pressure: 65 mmHg
111 mmHg
61 mmHg
109 mmHg
66 mmHg
107 mmHg
64 mmHg
112 mmHg
62 mmHg
Different exercise intensities:
Pedalling power
0W
25W
50W
75W
100W
125W
0W
2 mins
BP cuff measurement every 2 mins
Blood pressure (mmHg)
0 W
25 W
50 W
75 W
100 W
125 W
0 W
Time (s)
112 mmHg, 59 mmHg
111 mmHg
64 mmHg
125 mmHg
64 mmHg
125 mmHg
73 mmHg
133 mmHg
75 mmHg
146 mmHg
71 mmHg
117 mmHg
65 mmHg

Fig. S36.

Performance validation of the carotid ultrasound patch for blood pressure (BP) measurements. **a**, Blood pressure calibration workflow. First, systolic and diastolic blood pressure (p_{s} and p_{d}) are measured via a standard method, such as an invasive arterial line (which provides direct waveform data) or a cuff. Simultaneously, ultrasound is used to measure the transient cross-sectional diameter of the radial artery, from which the systolic and diastolic cross-sectional areas (A_{s} and A_{d}) are calculated. Second, the stiffness coefficient α of the radial artery is calculated using the formula shown in **a**. Notably, for a single subject, the parameters α and p_{d} do not change significantly along the arterial tree. Therefore, a calibration performed at the radial artery can be used to estimate blood pressure waveforms at other locations, such as the carotid artery. It is also important to note that α varies between individuals and must be recalibrated when the subject's physiological state changes (e.g., before and after exercise). Third, the carotid ultrasound patch is used for measuring the transient diameter of the carotid artery, allowing the continuous calculation of carotid cross-sectional area ($A(t) = \pi(d/2)^2$). Finally, the carotid blood pressure waveform is derived using the formula shown in **a**. **b**, Blood pressure calibration by gold standard measurement and validation of the BP waveform. Radial artery BP was measured using an invasive arterial line (A-line), and the calibration procedure followed the workflow described in **a**. The BP waveforms obtained via the A-line and ultrasound methods showed overall similar trends, with mean$\pm$std values of 93.0$\pm$21.4 and 90.5$\pm$20.9 mmHg, respectively. However, the tidal peak following the initial systolic peak was not clearly captured by ultrasound, likely due to the limited frame rate of B-mode imaging. In contrast, A-mode imaging enables more detailed waveform resolution (*23*). The calibration also yielded a stiffness coefficient of 2.22, and systolic and diastolic pressures of 149 and 69 mmHg. Subsequently, carotid BP waveforms were acquired using both the developed carotid ultrasound patch and a commercial ultrasound system (Mindray M9). The results demonstrated close agreement in waveform characteristics (mean$\pm$std values of 94.2$\pm$16.6 mmHg by ultrasound patch and 94.3$\pm$19.0 mmHg by commercial probe), confirming the performance of the developed patch. **c**, Blood pressure calibration by commercial BP cuff. Commercial cuff was chosen as a non-invasive alternative to the invasive arterial line (A-line) method. While the cuff on the participant's wrist provides only discrete systolic and diastolic readings (~112/62 mmHg), combining this with brachial artery diameter data from the ultrasound patch allows for calibration of the stiffness coefficient. After calibration, carotid artery BP was further measured by commercial ultrasound probe and carotid patch. Results show similar waveform characteristics, with mean$\pm$std values of 87.5$\pm$16.8 mmHg by ultrasound patch and 80.6$\pm$17.1 to 86.5$\pm$17.7 mmHg by commercial probe), demonstrating the reliable performance of the developed patch. For clarity and completeness, the data shown in Fig. 5G is also plotted in panels **b** and **c** here. **d**, Blood pressure at different postures and exercise intensities. To ensure accurate measurement across physiological states, blood pressure was calibrated under different conditions. First, systolic and diastolic pressures were recorded in multiple postures (seated, neck flexion, extension, and left/right rotation), revealing consistent values. This stability indicates that the arterial stiffness coefficient can be treated as a constant under static conditions. Subsequently, a dynamic bicycle exercise protocol was conducted. Cuff measurements taken immediately after each pedaling power level showed a progressive increase in blood pressure, with systolic pressure rising markedly from 109 to 146 mmHg and diastolic pressure increasing moderately from 59 to 75 mmHg. The stiffness coefficient was recalibrated at each power level, enabling the final calculation of carotid blood pressure waveforms throughout the exercise.

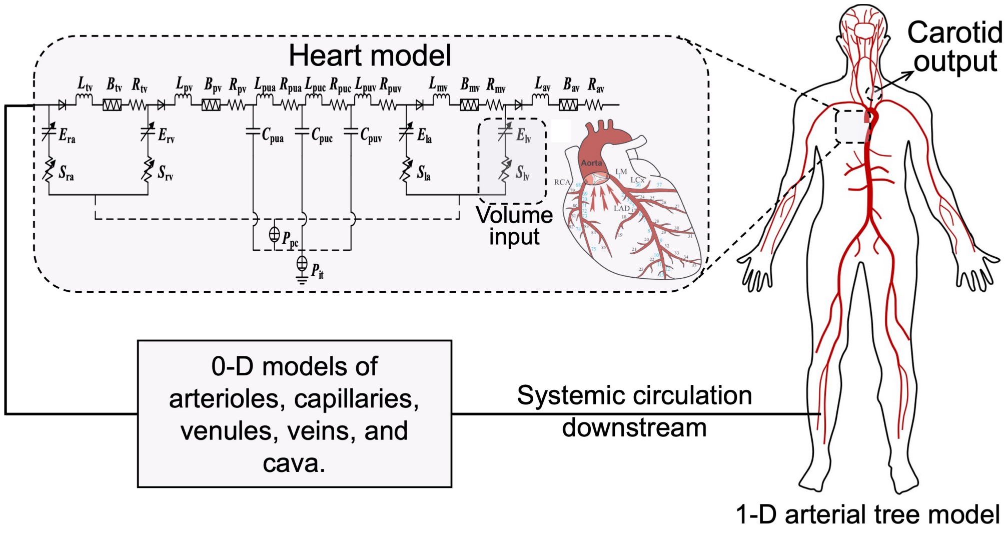

Fig. S37.

The developed human circulatory model. The hybrid model combines simplified 0-D and 1-D representations of the circulatory system. The 1-D component simulates arterial blood flow and wave propagation, while the 0-D lumped-parameter model (derived from linearized 1-D equations) captures flow dynamics in the heart, capillaries, and veins. The 1-D and 0-D components were modeled based on references (*51*, *80*, *81*). Positioned upstream of the arterial network, the heart model drives the entire circulatory system. Detailed equivalent parameters of the heart model are listed in table S3. Together, these coupled components enable complete circulatory simulation (see Materials and Methods for detailed descriptions). Using experimental measurements of time-varying left ventricular volume and heart rate as inputs, the model outputs key hemodynamic parameters at the common carotid artery, including blood pressure, vessel diameter, flow velocity, and pulse wave power. The models were illustrated using PowerPoint.

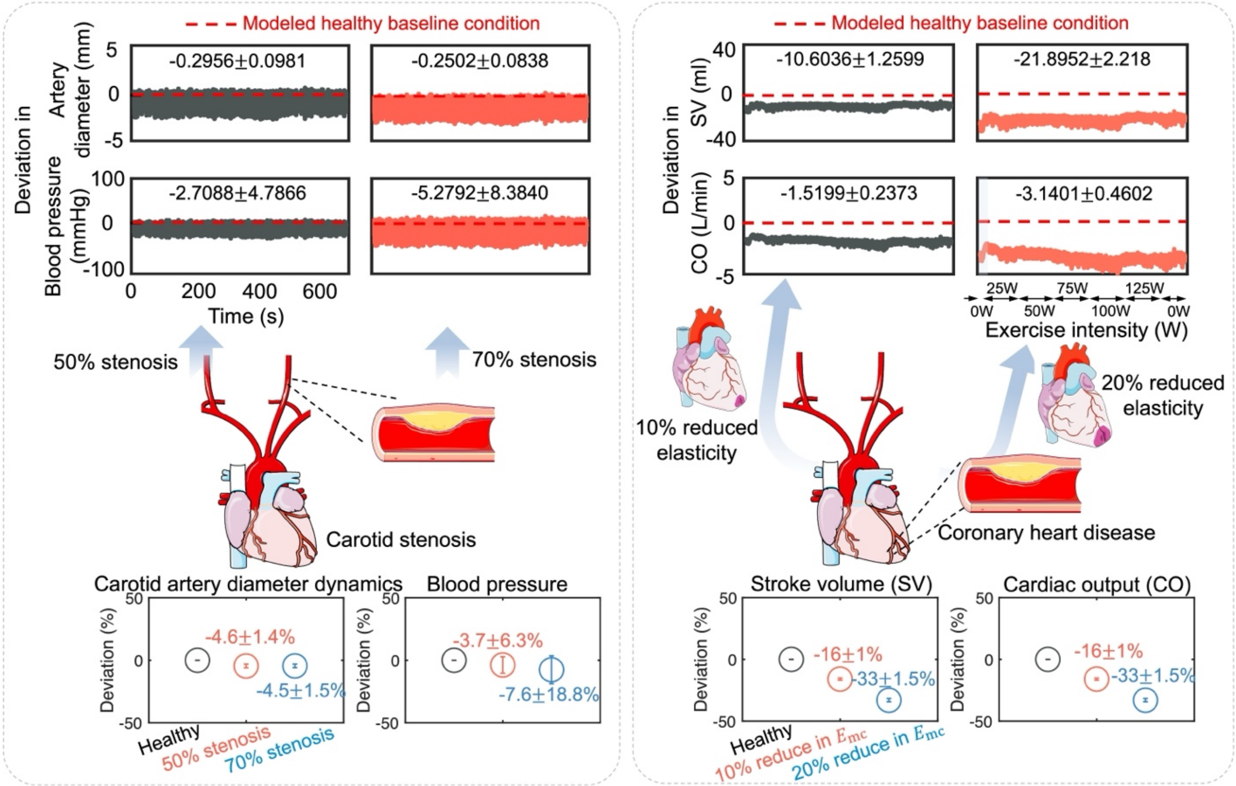

	50% stenosis		70% stenosis		10% reduced elasticity		20% reduced elasticity	
	Artery diameter	Blood pressure	Artery diameter	Blood pressure	CO	SV	CO	SV
Area under curve (AUC)	0.9572	0.5531	0.9313	0.6165	0.8061	0.9838	0.9775	1
95% confidence interval	[0.9537, 0.9602]	[0.5437, 0.5623]	[0.9271, 0.9353]	[0.6063, 0.6253]	[0.7985, 0.8132]	[0.9822, 0.9854]	[0.9751, 0.9796]	[1, 1]
Optimal threshold (Youden)	0.1319	0.2190	0.1977	0.3346	0.0551	0.0747	0.0257	0
Sensitivity at optimal	0.9262	0.3036	0.9087	0.5070	0.5865	0.9524	0.9122	1
Specificity at optimal	0.8681	0.7810	0.8023	0.6654	0.9449	0.9253	0.9743	1
Youden Index	0.7943	0.0846	0.7110	0.1723	0.5313	0.8776	0.8865	1
	Excellent separability	Poor separability	Excellent separability	Poor separability	Good separability	Excellent separability	Excellent separability	Excellent separability

Fig. S38.

Pathological modeling results. Through systematic introduction of pathological conditions (carotid artery stenosis and coronary heart disease, see Materials and Methods), the model reveals distinct hemodynamic alterations. A 50% stenosis is classified as a mild to moderate blockage, representing an early pathological stage, whereas 70% stenosis is considered severe. The arterial diameter and blood pressure show deviations from healthy condition, and are more sensitive to the degree of stenosis than to exercise intensity. Blood pressure shows an average deviation of -4% and -8% at 50% and 70% stenosis, respectively. The progression of coronary artery disease often leads to reduced myocardial compliance. A 10% reduction in compliance represents an early stage of CHD, while a 20% reduction indicates a moderate stage. Modeled SV and CO show clear deviations from the healthy baseline, demonstrating high sensitivity to changes in both myocardial compliance and exercise intensity. The deviation for both SV and CO ranges from -16% to -33% under a 10% and 20% reduction in compliance, respectively. Receiver-operating characteristic

(ROC) analysis shows that artery diameter, CO, and SV metrics exhibit good to excellent separability between pathological and healthy models. While blood pressure shows poor statistical separability, its high percentage deviation from the healthy baseline remains a useful distinguishing factor.

For the cardiac evaluation, a reduction in elasticity of 10% and 20% presents a CO/SV deviation of -16% and -33% from healthy baseline. Extrapolating from this trend via linear fitting allows us to probe the sensitivity limits of our model for detecting subtler, earlier pathological changes. The analysis indicates that a much smaller, 2% reduction in baseline elasticity would be feasible for detection, with a predicted -3.3±1.5% deviation from the healthy functional baseline. To contextualize the clinical relevance of this modeled sensitivity threshold, we have conducted a survey of detection limits inherent in current diagnostic modalities (*82*, *83*). The clinical gold standard for assessing myocardial function and structure is Cardiac Magnetic Resonance Imaging (MRI), which offers superior tissue characterization. Within the MRI suite, left ventricular systolic function is often precisely quantified using Global Longitudinal Strain (GLS), a sensitive parameter that measures the percentage shortening of the myocardium along its long axis during contraction. In clinical practice and supported by reproducibility studies, the smallest meaningful change in GLS that can be reliably detected, beyond the threshold of normal biological variation and inter-observer measurement error, is an absolute change of approximately 1.5 to 2.0 percentage points. For illustrative purposes, this means a clinically recognizable alteration would be a shift from a normal, healthy baseline strain of -20.0% (where the negative sign denotes shortening) to a pathologically reduced value of -18.5% or -18.0%. This established clinical sensitivity window provides a crucial real-world benchmark. The minimum detectable pathological deviation by our model (~2% reduced elasticity) and the established clinical standard (~1.5-2%) is relatively similar. It suggests that the level of pathological impairment our model identifies as theoretically discernible under conditions of high precision aligns with the actual limits of detection in contemporary clinical practice. The heart and carotid models were illustrated using PowerPoint.

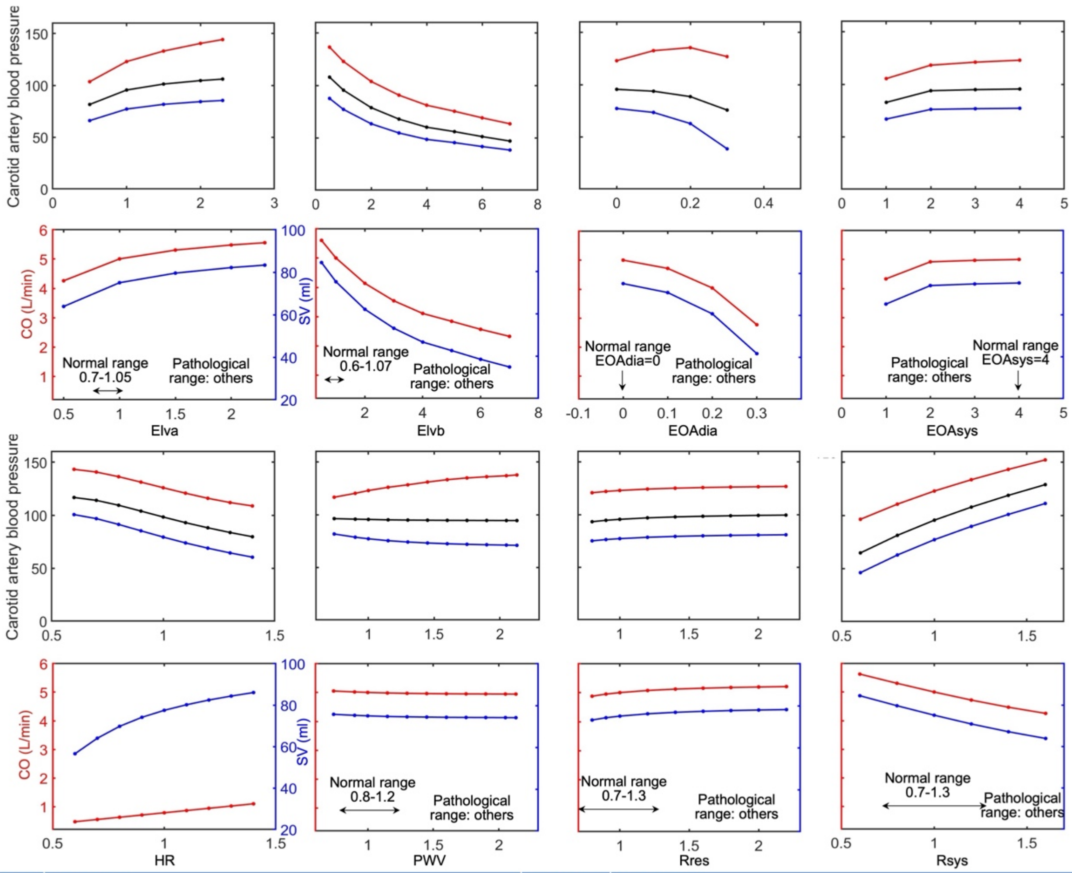

Parameter	Description	Parameter	Description
Elva	Left ventricular systolic compliance.<br>The value of Elva is set to 0.5, 1.0, 1.5, 2.0, and 2.3 (where 1.0 represents the normal state, 0.5 corresponds to 50% of the normal value, and so on for the others). 0.7-1.05 represent the normal ranges, and other values represent pathological ranges.	HR	Heart rate.<br>1.0 represents the normal heart rate, where 0.6 corresponds to 0.6 times the normal rate, and so on.
Elvb	Left ventricular diastolic compliance.<br>Elvb takes the values of 0.5, 1.0, 2.0, 3.0, 4.0, 5.0, 6.0, and 7.0 (where 1.0 represents the normal state, 0.5 corresponds to 50% of the normal value, and so on for the others). 0.6-1.07 represent the normal ranges, and other values represent pathological ranges.	PWV	An indicator of vascular stiffness.<br>The values range from 0.74 to 2.13, where 1.0 represents the normal state, 0.74 corresponds to 74% of the normal value, and so forth for other values. 0.8-1.2 represent the normal ranges, and other values represent pathological ranges.
EOAdia	Aortic valve regurgitation (characterized by the valve's diastolic opening area).<br>Under normal conditions, there is no regurgitation, corresponding to a value of 0.0 cm². With increasing severity of regurgitation, the assigned values are 0.1 cm², 0.2 cm², and 0.3 cm², representing the pathological ranges.	Rres	The magnitude of coronary microcirculatory impedance.<br>The value ranges from 0.8 to 2.2, where 1.0 represents the normal state, 0.8 corresponds to 80% of the normal value, and so on for the others. 0.7-1.3 represent the normal ranges, and other values represent pathological ranges.
EOAsys	Aortic valve stenosis (characterized by the valve's systolic opening area).<br>Under normal conditions, there is no stenosis, and the orifice area measures 4.0 cm². With increasing stenosis severity, the values assigned are 3.0 cm², 2.0 cm², and 1.0 cm², representing the pathological ranges.	Rsys	Peripheral circulatory impedance indirectly reflects a patient's blood pressure level.<br>The value ranges from 0.6 to 1.6, where 1.0 represents the normal state, 0.6 corresponds to 60% of the normal value, and so on for other values. 0.7-1.3 represent the normal ranges, and other values represent pathological ranges.

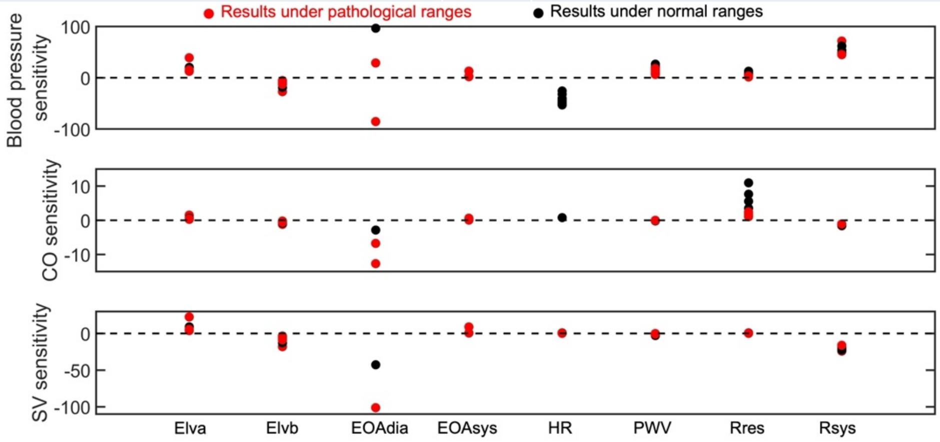

Fig. S39.

Sensitivity analysis of the modeling parameters. Model under different human-specific parameters were simulated (up panel), including compliance parameters (Elva, Elvb), aortic valve parameters (EOAdia, EOAsys), HR, carotid artery parameter (PWV), and impedance parameters (Rres, Rsys). These parameters were selected since they correspond to the most common physiological and pathological factors that have a significant impact on the closed-loop circulatory system (*84*). Variations of these parameters are explained in the middle panel. The normal range reflects physiological variation across individuals, while the pathological range indicates values associated with disease. Model outputs include systolic, diastolic, and mean blood pressure, as well as CO and SV. The bottom panel shows scatter plots of parameter sensitivity, calculated as the local slope (output difference/input difference) between adjacent data points from the upper panel. Black dots denote sensitivities derived from the normal range; red dots denote those from the pathological range. The sensitivity plots (bottom panel) identify the key parameters influencing each output. Under normal-range values, blood pressure is highly sensitive to EOAdia (aortic valve regurgitation), Rsys (systemic impedance), and HR (heart rate). For CO, the dominant parameter is Rres (coronary microcirculatory impedance); for SV, they are EOAdia and Rsys. Among these sensitive parameters, HR is a direct model input and can be customized for each individual. EOAdia, relevant for regurgitation, is effectively zero in healthy individuals. Rsys and Rres are holistic resistances, representing the distributed vascular impedances of the systemic circulation and myocardium, respectively, and they were modified by scaling all corresponding regional resistances simultaneously. Crucially, Rsys primarily affects blood pressure and SV, whereas Rres specifically influences CO, allowing their effects to be differentiated in the outputs. In practice, this sensitivity analysis might provide a calibration priority. If model outputs diverge significantly from measurements, parameters with the highest sensitivity for that output should be adjusted first to achieve better agreement.

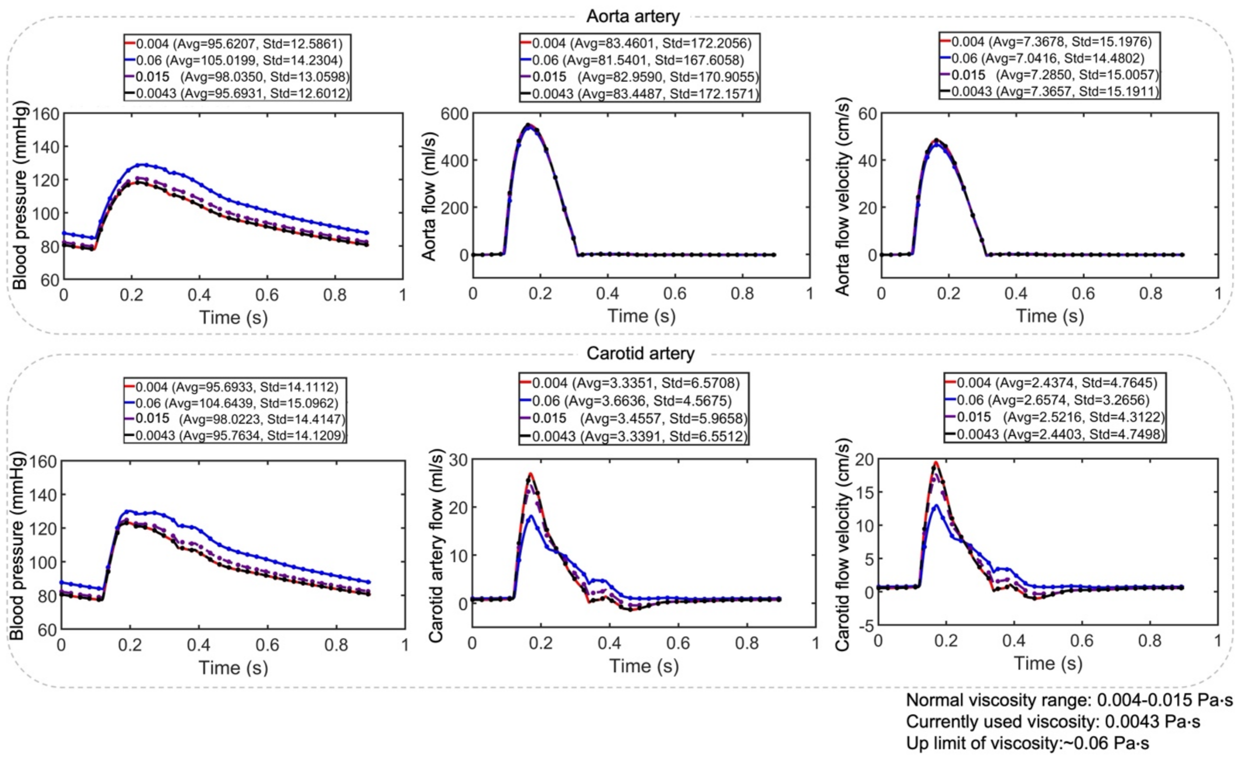

Fig. S40.

Modeling results of non-Newtonian flow. The human circuitry model assumes Newtonian flow. However, in practice, blood flow is non-Newtonian flow, which means viscosity is not a constant property, but changes with the applied shear stress or the rate of shearing, particularly in smaller vessels and at low shear rates. Currently, our model output focuses on the main (large-diameter) arteries (e.g., aorta, carotid). In these vessels, shear rates are typically very high (>100 s^{-1}). Under these conditions, the apparent viscosity of blood becomes nearly constant, and its behavior closely approximates a Newtonian fluid. We have added simulations to quantify the effects of blood viscosity. Within the typical physiological range (0.004 to 0.015 Pa·s), the resulting deviations from population-averaged values are limited. For the aorta, average deviations were 2.4 mmHg (2.5%) in pressure, -0.5 ml/s (-0.6%) in blood flow, and -0.08 cm/s (-1.1%) in flow velocity. For the carotid artery, average deviations were 2.3 mmHg (2.4%) in pressure, 0.12 ml/s (3.6%) in blood flow, and 0.08 cm/s (3.4%) in flow velocity. Overall, blood viscosity introduces an average deviation within 3.5% across key hemodynamic parameters.

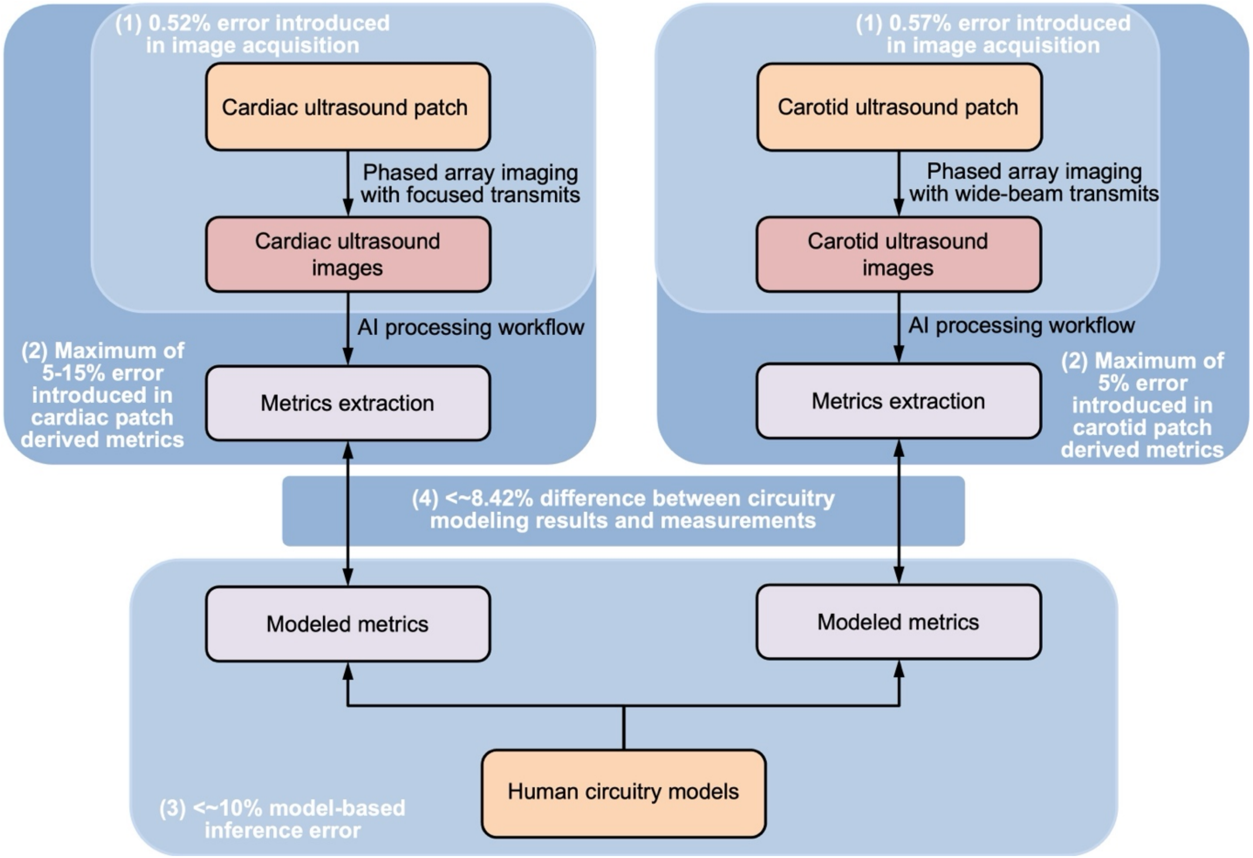

Fig. S41.

Illustration of the error components within the proposed framework. **(1) Errors introduced in image acquisition**: Errors may arise from factors such as ultrasound patch designs and beamforming processing. Ultrasound images of a standard phantom (fig. S12, fig. S14) revealed scatterers located near the target depths. Analysis showed that the depth of these scatterers had an average error of 0.52 ± 0.75% (with individual errors of 2.47%, 0.53%, 0.31%, 0.20%, 0.31%, 0.14%, 0.10%, and 0.07% for scatterers at 20, 40, 60, 80, 90, 100, 120, and 140 mm, respectively) for the cardiac patch, and 0.57 ± 1.0% (with individual errors of 2.00%, -0.085%, and -0.20% for scatterers at 6, 20, and 40 mm, respectively) for the carotid patch. The scatterer depth was determined by identifying the pixel with the highest image brightness near the target depth. These errors represent imaging inaccuracies that may propagate through the subsequent processing framework. **(2) Errors introduced in patch derived metrics**: It combines the errors from image acquisition, AI-based segmentation/reconstruction and metric extraction. To derive cardiac metrics, cardiac ultrasound images were first segmented using the MTANet architecture, then completed with a 2D inpainting model, and finally reconstructed using the nnU-Net model. During this process, errors may be introduced in both image segmentation and shape reconstruction. The AI-based metric extraction method was validated against a commercial ultrasound system under resting and post-exercise conditions (Fig. 4H and fig. S32). At rest, measurements from the cardiac patch showed the following differences (mean ± SD) compared to the clinical standard: end-diastolic volume (EDV): -5.97 ± 5.29 mL; end-systolic volume (ESV): 9.28 ± 3.83 mL; ejection fraction (EF): -0.0956 ± 0.0385; cardiac output (CO): 0.108 ± 0.443 L/min; and stroke volume (SV): -2.75 ± 6.81 mL. The majority of these differences remained within 5% of the clinical

standard, confirming the reliability of the cardiac patch measurements. Post exercise, the cardiac patch yielded respective differences of -1.22 ± 9.86 mL, -10.6 ± 6.2 mL, -0.0845 ± 0.0456, -0.658 ± 0.635 L/min, and 20.28 ± 9.77 mL, with most differences falling within 15% of the clinical standard. Therefore, the AI-based processing error for cardiac images is estimated to be at most 5-15%. To obtain carotid metrics, carotid ultrasound images were processed using the pre-trained nnU-Net deep learning model, during which image segmentation errors may be introduced. Blood pressure measurements obtained with the carotid patch were calibrated and validated against clinical standards, including the invasive arterial line and cuff method (Fig. 5G, fig. S36, Materials and Methods). Compared with the gold standard invasive arterial line method, the average radial artery blood pressure showed an error of approximately 2.7% (90.5 ± 20.9 mmHg vs. 93.0 ± 21.4 mmHg). Compared with measurements from a commercial ultrasound probe, the average carotid artery blood pressure showed an error of approximately 4.8% (87.5 ± 16.8 mmHg vs. 80.6 ± 17.1, 86.5 ± 17.7, and 83.7 ± 14.5 mmHg). **(3) Model-based inference error**: In our earlier study (*52*), we compared the accuracy of the 0-1D model used in the present work with clinically invasive measurements (invasively measured FFR). A multi-center, double-blind collaborative test was conducted on the same cohort of patients (10 individuals, 14 diseased coronary arteries) to evaluate the accuracy of various coronary fractional flow reserve (FFR) estimation techniques. Results showed that the errors between the calculated FFR values by our method and the measured values are mostly within 10% (*52*), and the accuracy in identifying hemodynamically significant FFR (<0.8) exceeded 80% for all groups. These findings indicate that 0-1D models can achieve a level of accuracy comparable to 3D models in characterizing hemodynamics in stenotic regions of real patient vessels. **(4) Comparison between circuitry modeling results and measurements**: Fig. 6B presents a comparison between the modeled results from healthy participants and the measurements obtained using the ultrasound patches, focusing on transient metric data under graded exercise intensity. The results show differences of 8.32% for SV and 8.42% for CO. Figs. 6D-E show the comparison between modeled pathological results and patch measurements, based on transient metric data under resting conditions. For the patient with coronary heart disease (CHD), the differences were -2.86% for SV and -4.11% for CO. For the patient with comorbid CAD and CHD, the differences were -7.31% for SV, -6.52% for CO, and -1.5% for blood pressure. Overall, the absolute differences remained within 8.42%. The error from each individual component in fig. S41 propagates through the framework and may be amplified or attenuated by subsequent steps. Therefore, rather than evaluating each error in isolation, we assessed the combined errors. Among the described error components above, the combined error from image acquisition, AI-based segmentation/reconstruction, and metric extraction (i.e., the errors introduced in patch-derived cardiac metrics, up to 5-15%) contributes the most to the uncertainty in the final diagnostic metrics comparison between modeling and measurements. This is followed by the model-based inference error (10%).

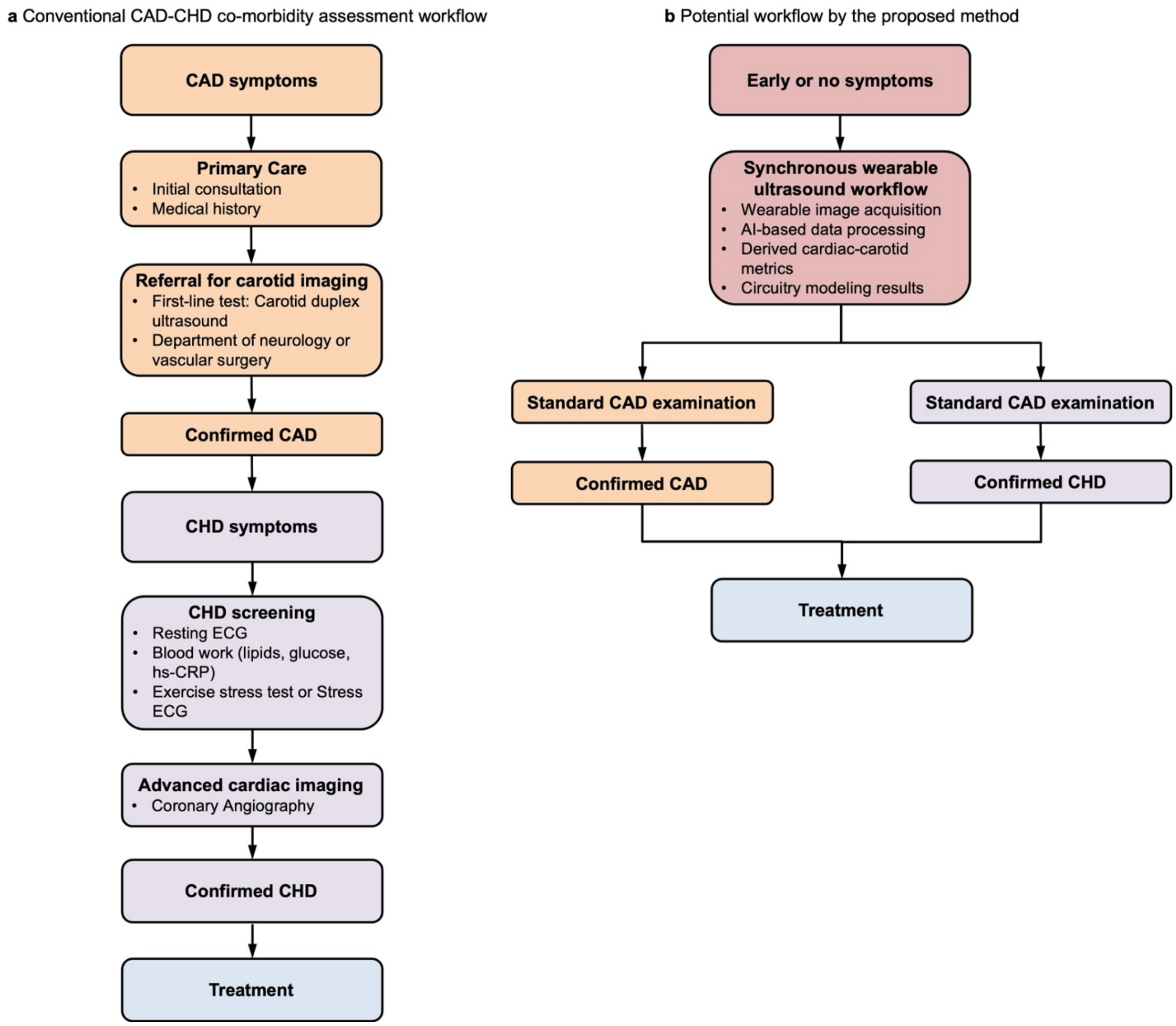

Fig. S42.

Potential role of the proposed method in the assessment of CAD-CHD co-morbidity. This study presents a framework for deriving clinically significant cardiac-carotid metrics using synchronous wearable ultrasound acquisition and AI-based data processing. Through model fitting of the measured metrics with assumed pathological parameters, the proposed method offers potential insights into the co-morbidity of CAD and CHD. As shown in fig. S42, we position this method as a diagnostic support tool specifically aimed at suggesting the possible presence or early signs of co-morbidity. The proposed framework is not intended to replace comprehensive clinical evaluation but rather to serve as an adjunct that highlights potential and early co-morbid patterns for further investigation. We also acknowledge that in typical clinical practice, more accurate evaluation would require integrating multiple information sources, including oral inquiry about physical condition, medical history, and physical examination. Our method alone cannot provide a definitive diagnosis; instead, it offers an additional, data-driven suggestion to guide clinicians regarding the presence and extent of co-morbidities. Nevertheless, we fully recognize that the gold standard remains necessary for confirmation. Our method potentially offers a more efficient approach for early intervention in comorbidity assessment and treatment, whereas conventional

diagnosis typically relies on separate, site-by-site examinations initiated only after symptoms appear.

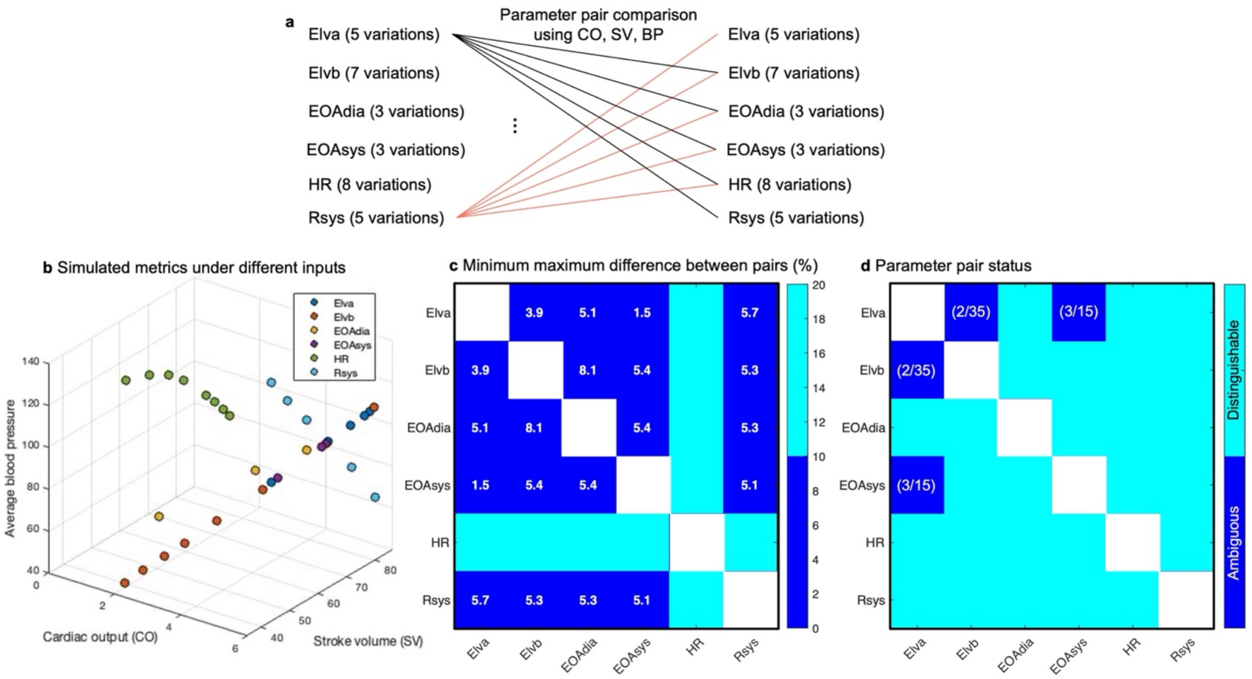

Fig. S43.

The identifiability issue of inverse mapping from measured metrics to disease states. **a**, Illustration of simulation parameter pairs. **b**, Simulated metrics under different inputs. **c**, Minimum maximum difference between pairs. **d**, Parameter pair status. We acknowledge that the inverse mapping from clinical metrics (such as CO, SV, BP) to disease states is not always unique, and diagnostic ambiguity is inherent to this model. To address this, we conducted an ambiguity analysis using 31 simulations covering 6 pathological parameters (Elva, Elvb, EOAdia, EOAsys, HR, Rsys, with the variation counts of 5, 7, 3, 3, 8, 5, respectively, as shown in Fig. S39). Each simulation varied only one parameter from healthy conditions. Outputs included CO, SV, and BP (minimum, mean, maximum values). Parameter pairs were compared across all 6 parameters (Fig. S42a), with each parameter pair includes multiple variation pairs. For example, the parameter pair Elva vs. Elvb includes 35 (5×7) variation pairs. A parameter pair was considered distinguishable if any output differed by >5% (minimum value of the maximum output difference across variation pairs >5%), where 5% reflects median measurement error in CAD/CHD patients (Fig. S41); ambiguity required all outputs to differ by <5%. As shown in figs. S43b-d, the inverse mapping is partially non-unique. 13 of 15 parameter pairs (86.7%) are clearly distinguishable. Two pairs showed ambiguity: Elva (left ventricular systolic compliance) vs. EOAsys (aortic valve stenosis) had 3 of 15 variation pairs with small differences (fig. S43d); Elva vs. Elvb (left ventricular diastolic compliance) had 2 of 35 variation pairs with small differences. For these cases, differences in all outputs fell within 5%, precluding reliable distinction given typical measurement variability. Raising the threshold from 5% to 10% increased ambiguous parameter pairs to 8, but only 11 of 390 variation pairs were ambiguous, primarily involving cardiac parameters (Elva, Elvb, EOAsys). In our CHD pathological modeling, Elva and Elvb co-vary (Materials and Methods), rendering their ambiguity physiologically reasonable. Thus, while most cases are identifiable, diagnostic ambiguity still exists, mainly between left ventricular elastance (Elva, Elvb) and aortic valve stenosis (EOAsys). Additional metrics extractable by our method (e.g., waveform features, HR, PR, RR, vessel dynamics) might be helpful for resolving these ambiguities, since combining richer

information enables more accurate evaluation. As Fig. S43 considers only limited parameter cases, further in-depth analysis remains a critical future direction.

Table S1.

The bicycle exercise protocol (*30*). Watts is the unit used to measure power output in spin biking, which quantifies the amount of energy a rider generates while pedaling. METS stands for Metabolic Equivalents, which are a measure of the energy cost of physical activities. One MET is defined as the amount of energy the body uses while at rest (sitting still). Increasing METS represents the rise of excise intensity, which was achieved by increasing the pedaling resistance. METS are used to classify activities as low (≤ 3 METs), moderate (3-6 METs), or high (> 6 METs) intensity. The participant pedals at each pedaling watts for around 2 mins, and may discontinue the exercise protocol at any time if they experience excessive fatigue or discomfort.

Stage	1	2	3	4	5	6	7	8	9	10
Watts	25	50	75	100	125	150	175	200	225	250
METS	2.4	3.7	4.9	6.1	7.3	8.6	9.8	11.0	12.2	13.5
Duration (mins)	2	2	2	2	2	2	2	2	2	2
Total time (mins)	2	4	6	8	10	12	14	16	18	20

Table S2.

Performance comparison against existing wearable ultrasound devices, in terms of center frequency, pitch, electro-mechanical coupling coefficient, bandwidth, array number, axial/lateral resolution, matching layer, SNR, flexibility, attachment stability, frame rates. The designed patches in this work feature relatively small pitches compared to existing alternatives, effectively suppressing imaging artifacts caused by acoustic side lobes. The broad bandwidth surpasses that of most reported patches in the literature. Through an optimized linear array design with matching layers, this work achieves spatial imaging resolutions comparable to those of more complex patch architectures, while maintaining simplicity and scalability. The patch array was intentionally designed to be inflexible. This eliminates the unknown spatial location-induced imaging artifacts inherent to flexible beamforming patches, which are difficult to mitigate. Utilizing ultrasound gel and adhesive tape for better acoustic coupling and attachment stability, our patch demonstrated robust long-term wearability over 24 hours and tolerance for 20 minutes of graded-intensity cycling, surpassing the performance of most existing designs.

Ref.	(*21*)	(*19*)	(*18*)	(*23*)	(*26*)	(*27*)	(*85*)	(*36*)	(*38*)	(*86*)	(*87*)	This work
Frame rates	200 Hz	20-30 Hz	/	/	1 Hz	1 Hz	50 Hz	/	/	/	/	15-20 Hz
Attachment stability	4 h Extensive head motions	24 h Bicycle	12 h Cycling	16 h	1 h	165 min Eccentric exercise	1 min Running	/	6 s	/	1 h	24 h daily wear 20 mins bicycle
Flexibility	Flexible	~110% stretching	20% uniaxial stretching	60%x 50%y	20% uniaxial stretching	~40% stretching	Inflexible	110.6% stretching	/	> 50% stretching	16% stretching	Inflexible
SNR	40-43 dB	30-45 dB	/	over 15 dB	26.8 dB	39 dB	/	/	/	~20.24 dB	15-50 dB	25.84 dB 26.32 dB
Matching layer	No	No	No	Yes	No	No	No	Yes	Yes	No	No	Yes
Axial/lateral resolution	/	0.59-1/ 1.55-2.8 mm	0.23-1.98/ 1-5 mm	0.4/0.77 mm	/	0.5-1.5/ ~3 mm	0.34 mm	0.31-0.34/ 0.46-0.84 mm	0.24-0.26/ 0.38-0.68 mm	0.61 mm/ 0.34-0.79 mm	2.5 mm	0.5-1.1 mm/0.9-4.5 mm; 0.4-0.6 mm/0.4-1.4 mm

Array number	16×16	32	32/112/256	4×5	15×16	16×16	1	64	128	10×10	12×12	64
Bandwidth	17.41%	55%	~50%	32%	61%	50%	45%	66.47%	69.2± 7.65%	47.11%	/	60% 63%
Electro-mechanical coupling coefficient	0.65-0.7	0.67	/	/	0.6	0.64	/	0.66	0.68± 0.3	0.6	0.6	0.49 0.74
Pitch	0.75 mm	0.4 mm	0.8 mm	/	0.8 mm	0.8 mm	/	0.5 mm	0.3 mm	2.0 mm	0.8 mm × 0.8 mm	0.3 mm 0.27 mm
Center frequency	2 MHz	3 MHz	2/4/6 MHz	7.5 MHz	2.4 MHz	3 MHz	4 MHz	5 MHz	6.5 MHz	3.5 MHz	2 MHz	3 MHz 4.5 MHz

Table S3.

Lumped parameters of the heart model. Units of parameters are as follows: elastance (*E*), mmHg/ml; viscoelasticity coefficient (*S*), mmHg·s·ml^{-1}; resistance (*R*), mmHg·s·ml^{-1}; inertance (*L*), mmHg·s2·ml^{-1}; compliance (*C*), ml/mmHg; Bernoulli's resistance (*B*), mmHg·s^{2}·ml^{-2}; pressure (*P*), mmHg. Details are described in the reference (*81*). P_{la} and P_{lv} are derived from simulations through the model.

Parameters	Value	Parameters	Value	Parameters	Value
E_{raa}	0.13	L_{pua}	5e-4	S_{la}	$P_{la} \times 5\text{e} - 4$
S_{ra}	$P_{ra} \times 5\text{e} - 4$	C_{pua}	2.45	L_{mv}	5e-4
L_{tv}	5e-4	R_{pua}	0.035	B_{mv}	1e-3
B_{tv}	1e-5	L_{puc}	2e-4	R_{mv}	1e-3
R_{tv}	1e-3	C_{puc}	19.89	E_{lva}	2.87
E_{rva}	0.48	R_{puc}	0.0259	S_{lv}	$P_{lv} \times 5\text{e} - 4$
S_{rv}	$P_{rv} \times 5\text{e} - 4$	L_{puv}	3e-4	L_{av}	5e-4
L_{pv}	5e-4	C_{puv}	8.26	B_{av}	1.5e-4
B_{pv}	1.5e-5	R_{puv}	0.0152	R_{av}	1.5e-3
R_{pv}	1.5e-3	E_{laa}	0.25	P_{pc}	3
E_{rab}	0.13	E_{rvb}	0.05	P_{it}	-3.5
E_{lvb}	0.056	E_{lab}	0.25		

Table S4.

Two groups of participants recruited for in-vivo tests. The first group comprised five healthy individuals who completed the bicycle exercise protocol, where intensity was systematically increased from rest (0 W) to peak effort (125 W) in 25 W increments every two minutes. The second group consisted of three patients with confirmed CAD, CHD, or CAD-CHD co-morbidity, who were measured at the resting state for safety considerations.

	Health condition	Age	Sex	BMI (kg/m^2)	Adverse events during test
Group 1	Healthy	24	m	19.9	All completed graded intensity exercise from 0 to 125 W, no adverse event
		31	m	19.6	
		24	m	23.4	
		26	m	24.4	
		20	m	23.9	
Group 2	Confirmed CAD (unstable plaque at the origin of the left internal carotid artery, causing severe luminal stenosis)	65	m	22	Resting state test, no adverse event
	Confirmed CHD (Acute anterior myocardial infarction: occasional atrial premature beats, nonspecific intraventricular conduction block, segmental wall motion abnormalities of the left ventricle)	30	m	23.4	
	Confirmed CAD-CHD comorbidity (CHD: occasional atrial premature beats, trace mitral and tricuspid regurgitation; CAD: focal intima-media thickening with plaque formation in bilateral carotid arteries)	61	m	22	

Movie S1.
Cardiac ultrasound image segmentation.

Movie S2.
Carotid ultrasound image segmentation.

REFERENCES

1. P. Libby, Inflammation during the life cycle of the atherosclerotic plaque. *Cardiovasc. Res.* **117**, 2525–2536 (2021).

2. K. Yahagi, F. D. Kolodgie, C. Lutter, H. Mori, M. E. Romero, A. V. Finn, R. Virmani, Pathology of human coronary and carotid artery atherosclerosis and vascular calcification in diabetes mellitus. *Arterioscler. Thromb. Vasc. Biol.* **37**, 191–204 (2017).

3. S. Tanimoto, Y. Ikari, K. Tanabe, S. Yachi, H. Nakajima, T. Nakayama, M. Hatori, G. Nakazawa, Y. Onuma, Y. Higashikuni, H. Yamamoto, E. Tooda, K. Hara, Prevalence of carotid artery stenosis in patients with coronary artery disease in Japanese population. *Stroke* **36**, 2094–2098 (2005).

4. D. Bos, B. Arshi, Q. J. A. van den Bouwhuijsen, M. K. Ikram, M. Selwaness, M. W. Vernooij, M. Kavousi, A. van der Lugt, Atherosclerotic carotid plaque composition and incident stroke and coronary events. *J. Am. Coll. Cardiol.* **77**, 1426–1435 (2021).

5. F. Jashari, P. Ibrahimi, R. Nicoll, G. Bajraktari, P. Wester, M. Y. Henein, Coronary and carotid atherosclerosis: A reflection of systemic atherosclerosis. *Atherosclerosis* **227**, 193–200 (2013).

6. V. Aboyans, J. B. Ricco, M. E. L. Bartelink, M. Bjorck, M. Brodmann, T. Cohnert, J. P. Collet, M. Czerny, M. De Carlo, S. Debusa, C. Espinola-Klein, T. Kahan, S. Kownator, L. Mazzolai, A. R. Naylora, M. Roffi, J. Rotherb, M. Sprynger, M. Tendera, G. Tepe, M. Venermo, C. Vlachopoulos, I. Desormais, ESC Scientific Document Group. 2017 ESC guidelines on the diagnosis and treatment of peripheral arterial diseases, in collaboration with the European Society for Vascular Surgery (ESVS): Document covering atherosclerotic disease of extracranial carotid and vertebral, mesenteric, renal, upper and lower extremity arteries. Endorsed by: The European Stroke Organization (ESO). The task force for the diagnosis and treatment of peripheral arterial diseases of the European Society of Cardiology (ESC) and of the European Society for Vascular Surgery (ESVS). *Eur. Heart J.* **39**, 763–816 (2018).

7. I. Bytyçi, R. Shenouda, P. Wester, M. Y. Henein, Carotid atherosclerosis in predicting coronary artery disease: A systematic review and meta-analysis. *Arterioscler. Thromb. Vasc. Biol.* **41**, e224–e237 (2021).

8. S. Duvnjak, D. Radak, *Diagnosis of Carotid Artery Diseases in Carotid Artery Disease* (Springer, Switzerland, 2025).

9. S. N. Hayes, M. S. Tweet, D. Adlam, E. S. H. Kim, R. Gulati, J. E. Price, C. H. Rose, Spontaneous coronary artery dissection: JACC state-of-the-art review. *J. Am. Coll. Cardiol.* **76**, 961–984 (2020).

10. N. S. Nurmohamed, A. R. van Rosendael, I. Danad, Q. Ngo-Metzger, P. R. Taub, K. K. Ray, G. Figtree, M. P. Bonaca, J. Hsia, F. Rodriguez, A. T. Sandhu, K. Nieman, J. P. Earls, U. Hoffmann, J. J. Bax, J. K. Min, D. J. Maron, D. L. Bhatt, Atherosclerosis evaluation and cardiovascular risk estimation using coronary computed tomography angiography. *Eur. Heart J.* **45**, 1783–1800 (2024).

11. L. E. Mantella, A. M. Johri, A call for non-invasive tools to assess cardiovascular risk. *J. Am. Soc. Echocardiogr.* **34**, A11–A12 (2021).

12. W. Brinjikji, J. Huston III, A. A. Rabinstein, G. M. Kim, A. Lerman, G. Lanzino, Contemporary carotid imaging: From degree of stenosis to plaque vulnerability. *J. Neurosurg.* **124**, 27–42 (2016).

13. V. Rafailidis, I. Chryssogonidis, C. Xerras, I. Nikolaou, T. Tegos, K. Kouskouras, D. Rafailidis, A. Charitanti-Kouridou, A comparative study of color Doppler imaging and contrast-enhanced ultrasound for the detection of ulceration in patients with carotid atherosclerotic disease. *Eur. Radiol.* **29**, 2137–2145 (2019).

14. M. S. Williams, G. N. Levine, D. Kalra, A. Agarwala, D. Baptiste, J. E. Cigarroa, R. L. Diekemper, M. V. Foster, M. Gulati, T. D. Henry, D. Itchhaporia, J. S. Lawton, L. K. Newby, K. C. Rogers, K. Soni, J. E. Tamis-Holland, 2025 AHA/ACC clinical performance and quality measures for patients with chronic coronary disease: A report of the American College of

Cardiology/American Heart Association Joint Committee on Performance Measures. *J. Am. Coll. Cardiol.* **85**, 2504–2535 (2025).

15. R. Maini, J. Moscona, G. Sidhu, P. Katigbak, C. Fernandez, A. Irimpen, O. Mogabgab, C. Ward, R. Samson, T. LeJemtel, Pooled diagnostic accuracy of resting distal to aortic coronary pressure referenced to fractional flow reserve: The importance of resting coronary physiology. *J. Interv. Cardiol.* **31**, 588–598 (2018).

16. U. Baber, R. Mehran, S. Sartori, M. M. Schoos, H. Sillesen, P. Muntendam, M. J. Garcia, J. Gregson, S. Pocock, E. Falk, V. Fuster, Prevalence, impact, and predictive value of detecting subclinical coronary and carotid atherosclerosis in asymptomatic adults: The BioImage study. *J. Am. Coll. Cardiol.* **65**, 1065–1074 (2015).

17. S. Zhou, G. Park, M. Y. Lin, X. Y. Yang, S. Xu, Wearable ultrasound technology. *Nat. Rev. Bioeng.* **3**, 835–854 (2025).

18. M. Lin, Z. Zhang, X. Gao, Y. Bian, R. S. Wu, G. Park, Z. Lou, Z. Zhang, X. Xu, X. Chen, A. Kang, X. Yang, W. Yue, L. Yin, C. Wang, B. Qi, S. Zhou, H. Hu, H. Huang, M. Li, Y. Gu, J. Mu, A. Yang, A. Yaghi, Y. Chen, Y. Lei, C. Lu, R. Wang, J. Wang, S. Xiang, E. B. Kistler, N. Vasconcelos, S. Xu, A fully integrated wearable ultrasound system to monitor deep tissues in moving subjects, *Nat. Biotechnol.* **42**, 448–457 (2024).

19. H. Hu, H. Huang, M. Li, X. Gao, L. Yin, R. Qi, R. S. Wu, X. Chen, Y. Ma, K. Shi, C. Li, T. M. Maus, B. Huang, C. Lu, M. Lin, S. Zhou, Z. Lou, Y. Gu, Y. Chen, Y. Lei, X. Wang, R. Wang, W. Yue, X. Yang, Y. Bian, J. Mu, G. Park, S. Xiang, S. Cai, P. W. Corey, J. Wang, S. Xu, A wearable cardiac ultrasound imager. *Nature* **613**, 667–675 (2023).

20. H. Tang, Y. Yang, Z. Liu, W. Li, Y. Zhang, Y. Huang, T. Kang, Y. Yu, N. Li, Y. Tian, X. Liu, Y. Cheng, Z. Yin, X. Jiang, X. Chen, J. Zang, Injectable ultrasonic sensor for wireless monitoring of intracranial signals. *Nature* **630**, 84–90 (2024).

21. S. Zhou, X. Gao, G. Park, X. Yang, B. Qi, M. Lin, H. Huang, Y. Bian, H. Hu, X. Chen, R. S. Wu, B. Liu, W. Yue, C. Lu, R. Wang, P. Bheemreddy, S. Qin, A. Lam, K. A. Wear, M. Andre, E.

B. Kistler, D. W. Newell, S. Xu, Transcranial volumetric imaging using a conformal ultrasound patch. *Nature* **629**, 810–818 (2024).

22. A. Y. Chang, M. Lin, L. Yin, M. Reynoso, S. Ding, R. Liu, Y. Dugas, A. Casanova, G. Park, Z. Li, H. Luan, N. Askarinam, F. Zhang, S. Xu, J. Wang, Integration of chemical and physical inputs for monitoring metabolites and cardiac signals in diabetes. *Nat. Biomed. Eng.* **10**, 94–109 (2026).

23. C. Wang, X. Li, H. Hu, L. Zhang, Z. Huang, M. Lin, Z. Zhang, Z. Yin, B. Huang, H. Gong, S. Bhaskaran, Y. Gu, M. Makihata, Y. Guo, Y. Lei, Y. Chen, C. Wang, Y. Li, T. Zhang, Z. Chen, A. P. Pisano, L. Zhang, Q. Zhou, S. Xu. Monitoring of the central blood pressure waveform via a conformal ultrasonic device. *Nat. Biomed. Eng.* **2**, 687–695 (2018).

24. S. Zhou, G. Park, K. Longardner, M. Lin, B. Qi, X. Yang, X. Gao, H. Huang, X. Chen, Y. Bian, H. Hu, R. S. Wu, W. Yue, M. Li, C. Lu, R. Wang, S. Qin, E. Tasali, T. Karrison, I. Thomas, B. Smarr, E. B. Kistler, B. A. Khiami, I. Litvan, S. Xu, Clinical validation of a wearable ultrasound sensor of blood pressure. *Nat. Biomed. Eng.* **9**, 865–881 (2025).

25. F. Wang, P. Jin, Y. Feng, J. Fu, P. Wang, X. Liu, Y. Zhang, Y. Ma, Y. Yang, A. Yang, X. Feng, Flexible Doppler ultrasound device for the monitoring of blood flow velocity. *Sci. Adv.* **7**, eabi9283 (2021).

26. X. Gao, X. Chen, H. Hu, X. Wang, W. Yue, J. Mu, Z. Lou, R. Zhang, K. Shi, X. Chen, M. Lin, B. Qi, S. Zhou, C. Lu, Y. Gu, X. Yang, H. Ding, Y. Zhu, H. Huang, Y. Ma, M. Li, A. Mishra, J. Wang, S. Xu, A photoacoustic patch for three-dimensional imaging of hemoglobin and core temperature. *Nat. Commun.* **13**, 7757 (2022).

27. H. Hu, Y. Ma, X. Gao, D. Song, M. Li, H. Huang, X. Qian, R. Wu, K. Shi, H. Ding, M. Lin, X. Chen, W. Zhao, B. Qi, S. Zhou, R. Chen, Y. Gu, Y. Chen, Y. Lei, C. Wang, C. Wang, Y. Tong, H. Cui, A. Abdal, Y. Zhu, X. Tian, Z. Chen, C. Lu, S. Xu, Stretchable ultrasonic arrays for the three-dimensional mapping of the modulus of deep tissue, *Nat. Biomed. Eng.* **7**, 1321–1334 (2023).

28. Y. S. Chatzizisis, V. L. Murthy, S. D. Solomon, Echocardiographic evaluation of coronary artery disease. *Coron. Artery Dis.* **24**, 613–623 (2013).

29. S. A. Coulter, "Echocardiographic evaluation of coronary artery disease" in *Cardiovascular Medicine* (Springer, London, ed. 3, 2007), pp. 824–852.

30. P. A. Pellikka, A. Arruda-Olson, F. A. Chaudhry, M. H. Chen, J. E. Marshall, T. R. Porter, S. G. Sawada, Guidelines for performance, interpretation, and application of stress echocardiography in ischemic heart disease: From the American Society of Echocardiography. *J. Am. Soc. Echocardiogr.* **33**, 1–41.e8 (2020).

31. C. Mitchell, P. S. Rahko, L. A. Blauwet, B. Canaday, J. A. Finstuen, M. C. Foster, K. Horton, K. O. Ogunyankin, R. A. Palma, E. J. Velazquez, Guidelines for performing a comprehensive transthoracic echocardiographic examination in adults: Recommendations from the American Society of Echocardiography. *J. Am. Soc. Echocardiogr.* **32**, 1–64 (2019).

32. P. Hoskins, A. Thrush, K. Martin, *Diagnostic Ultrasound: Physics and Equipment* (Cambridge Univ. Press, Cambridge, ed. 2, 2010).

33. M. A. Chamsi-Pasha, P. P. Sengupta, W. A. Zoghbi, Handheld echocardiography: Current state and future perspectives. *Circulation* **136**, 2178–2188 (2017).

34. A. M. Johri, V. Nambi, T. Z. Naqvi, S. B. Feinstein, E. S. H. Kim, M. M. Park, H. Becher, H. Sillesen, Recommendations for the assessment of carotid arterial plaque by ultrasound for the characterization of atherosclerosis and evaluation of cardiovascular risk: From the American Society of Echocardiography. *J. Am. Soc. Echocardiogr.* **33**, 917–933 (2020).

35. S. S. Hans, M. F. Rehman, *Extracranial Carotid and Vertebral Artery Disease (Contemporary Management)* (Springer, Switzerland, ed. 2, 2025).

36. J. Chen, J. Liu, W. Chen, D. Shang, Q. Zhang, Y. Li, H. Zheng, D. Gu, D. Wu, T. Ma, Skin-conformable flexible and stretchable ultrasound transducer for wearable imaging. *IEEE Trans. Ultrason. Ferroelectr. Freq. Control* **71**, 811–820 (2024).

37. J. Chen, B. Zhuang, J. Peng, Z. Zhang, B. Wang, C. Dai, D. Wu, Variable curvature flexible transducer for abdominal expandable imaging. *IEEE Trans. Ultrason. Ferroelectr. Freq. Control* **72**, 299–308 (2025).

38. W. Chen, J. Liu, S. Lei, Z. Yang, Q. Zhang, Y. Li, J. Huang, Y. Dong, H. Zheng, D. Wu, T. Ma, Flexible ultrasound transducer with embedded optical shape sensing fiber for biomedical imaging applications. *I.E.E.E. Trans. Biomed. Eng.* **70**, 2841–2851 (2023).

39. Y. C. Eldar, *Sampling Theory: Beyond Bandlimited Systems* (Cambridge Univ. Press, London, 2014).

40. D. Zhang, W. Wang, F. Li, Association between resting heart rate and coronary artery disease, stroke, sudden death and noncardiovascular diseases: A meta-analysis. *CMAJ* **188**, E384–E392 (2016).

41. R. Ma, J. Gao, S. Mao, Z. Wang, Association between heart rate and cardiovascular death in patients with coronary heart disease: A NHANES-based cohort study. *Clin. Cardiol.* **45**, 574–582 (2022).

42. A. Mertins, D. A. Mertins, *Signal Analysis: Wavelets, Filter Banks, Time Frequency Transforms and Applications* (Wiley, England, 1999).

43. R. J. H. Miller, A. Killekar, A. Shanbhag, B. Bednarski, A. M. Michalowska, T. D. Ruddy, A. J. Einstein, D. E. Newby, M. Lemley, K. Pieszko, S. D. Van Kriekinge, P. B. Kavanagh, J. X. Liang, C. Huang, D. Dey, D. S. Berman, P. J. Slomka, Predicting mortality from AI cardiac volumes mass and coronary calcium on chest computed tomography. *Nat. Commun.* **15**, 2747 (2024).

44. C. C. Nwabuo, Y. Yano, H. T. Moreira, D. Appiah, H. D. Vasconcellos, Q. N. Aghaji, A. J. Viera, J. S. Rana, R. V. Shah, V. L. Murthy, N. B. Allen, P. J. Schreiner, D. M. Lloyd-Jones, J. A. C. Lima, Long-term blood pressure variability in young adulthood and coronary artery calcium and carotid intima-media thickness in mid-life: The CARDIA study. *Hypertension* **76**, 404–409 (2020).

45. Y. Ling, Y. Wang, W. Dai, J. Yu, P. Liang, D. Kong, MTANet: Multi-Task attention network for automatic medical image segmentation and classification. *IEEE. Trans. Med. Imaging* **43**, 674–685 (2024).

46. F. Isensee, W. Tassilo, U. Constantin, B. Michael, R. Saikat, M. Klaus, F. J. Paul, "nnU-Net revisited: A call for rigorous validation in 3D medical image segmentation," in *International Conference on Medical Image Computing and Computer-Assisted Intervention* (Springer Cham, 2024), vol. **15009**.

47. F. Isensee, P. F. Jaeger, S. A. Kohl, J. Petersen, K. H. Maier-Hein, nnU-Net: A self-configuring method for deep learning-based biomedical image segmentation. *Nat. Methods* **18**, 203–211 (2021).

48. X. Huang, M. A. Lediju Bell, K. Ding, Deep learning for ultrasound beamforming in flexible array transducer. *IEEE. Trans. Med. Imaging* **40**, 3178–3189 (2021).

49. J. Chang, Z. Chen, Y. Huang, Y. Li, X. Zeng, C. Lu, Flexible ultrasonic array for breast-cancer diagnosis based on a self-shape-estimation algorithm. *Ultrasonics* **108**, 106212 (2020).

50. X. Ge, Z. Yin, Y. Fan, Y. Vassilevski, F. Liang, A multi-scale model of the coronary circulation applied to investigate transmural myocardial flow. *Int. J. Numer. Method. Biomed. Eng.* **34**, e3123 (2018).

51. X. Ge, Y. Liu, S. Tu, S. Simakov, Y. Vassilevski, F. Liang, Model-based analysis of the sensitivities and diagnostic implications of FFR and CFR under various pathological conditions. *Int. J. Numer. Method. Biomed. Eng.* **37**, e3257 (2021).

52. J. M. Carson, S. Pant, C. Roobottom, R. Alcock, P. Javier Blanco, C. Alberto Bulant, Y. Vassilevski, S. Simakov, T. Gamilov, R. Pryamonosov, F. Liang, X. Ge, Y. Liu, P. Nithiarasu, Non-invasive coronary CT angiography-derived fractional flow reserve: A benchmark study comparing the diagnostic performance of four different computational methodologies. *Int. J. Numer. Method. Biomed. Eng.* **35**, e3235 (2019).

53. Y. Sun, M. Beshara, R. J. Lucariello, S. A. Chiaramida, A comprehensive model for right-left heart interaction under the influence of pericardium and baroreflex. *Am. J. Physiol.* **272**, H1499–H1515 (1997).

54. F. Liang, H. Senzaki, C. Kurishima, K. Sughimoto, R. Inuzuka, H. Liu, Hemodynamic performance of the Fontan circulation compared with a normal biventricular circulation: A computational model study. *Am. J. Physiol. Heart Circ. Physiol.* **1**, H1056–H1072 (2014).

55. D. F. Young, F. Y. Tsai, Flow characteristics in models of arterial stenoses. II. Unsteady flow. *J. Biomech.* **6**, 547–559 (1973).

56. C. J. White, T. G. Brott, W. A. Gray, D. Heck, T. Jovin, S. P. Lyden, D. C. Metzger, K. Rosenfield, G. Roubin, R. Sachar, A. Siddiqui, Carotid artery stenting: JACC state-of-the-art review. *J. Am. Coll. Cardiol.* **80**, 155–170 (2022).

57. M. Teramoto, Y. Kokubo, A. Arafa, R. Kashima, Y. M. Nakao, H. A. Sheerah, H. Kataoka, Common carotid artery stenosis degree as a predictor of cardiovascular disease in a general population: The Suita Study. *J. Am. Heart Assoc.* **13**, e030828 (2024).

58. A. Xodo, A. Gregio, F. Pilon, D. Milite, T. H. Danesi, G. Badalamenti, S. Lepidi, M. D'Oria, Carotid interventions in patients undergoing coronary artery bypass grafting: A narrative review. *J. Clin. Med.* **13**, 3019 (2024).

59. R. J. Henning, B. L. Hoh, The diagnosis and treatment of asymptomatic and symptomatic patients with carotid artery stenosis. *Curr. Probl. Cardiol.* **50**, 102992 (2025).

60. T. G. Brott, G. S. Roubin, W. S. Moore, J. F. Meschia, Carotid artery disease: Then and now. *Stroke. Vasc. Interv. Neurol.* **4**, e001076 (2024).

61. L. Saba, R. Scicolone, E. Johansson, V. Nardi, G. Lanzino, S. K. Kakkos, G. Pontone, A. D. Annoni, K. I. Paraskevas, A. J. Fox, Quantifying carotid stenosis: History, current applications, limitations, and potential: How imaging is changing the scenario. *Life* **14**, 73 (2024).

62. D. Hong, N. Dai, S. H. Lee, D. Shin, K. H. Choi, S. M. Kim, H. K. Kim, K. H. Jeon, S. J. Ha, K. Y. Lee, T. K. Park, J. H. Yang, Y. B. Song, J. Y. Hahn, S. H. Choi, Y. H. Choe, H. C. Gwon, J. Ge, J. M. Lee, Fractional flow reserve and fractional flow reserve gradient from CCTA for predicting future coronary events. *JACC Asia* **4**, 735–747 (2024).

63. G. Kossoff, The effects of backing and matching on the performance of piezoelectric ceramic transducers. *IEEE Trans. Sonics Ultrason.* **13**, 20–30 (1966).

64. D. Aune, A. Sen, B. ó'Hartaigh, I. Janszky, P. R. Romundstad, S. Tonstad, L. J. Vatten, Resting heart rate and the risk of cardiovascular disease, total cancer, and all-cause mortality—A systematic review and dose–response meta-analysis of prospective studies. *Nutr. Metab. Cardiovasc. Dis.* **27**, 504–517 (2017).

65. K. Fox, J. S. Borer, A. J. Camm, N. Danchin, R. Ferrari, J. L. Lopez Sendon, P. G. Steg, J. C. Tardif, L. Tavazzi, M. Tendera, Resting heart rate in cardiovascular disease. *J. Am. Coll. Cardiol.* **50**, 823–830 (2007).

66. F. Shaffer, J. P. Ginsberg, An overview of heart rate variability metrics and norms. *Front. Public Health* **5**, 258 (2017).

67. J. Osei, V. Vaccarino, M. Wang, A. S. Shah, R. Lampert, L. Y. Li, Y. A. Ko, B. D. Pearce, M. Kutner, E. V. Garcia, M. Piccinelli, P. Raggi, J. D. Bremner, A. A. Quyyumi, Y. V. Sun, H. Ahmed, G. Haddad, O. Daaboul, T. Roberts, L. Stefanos, L. Correia, A. J. Shah, Stress-induced autonomic dysfunction is associated with mental stress–induced myocardial ischemia in patients with coronary artery disease. *Circ. Cardiovasc. Imaging* **17**, e016596 (2024).

68. L. Tomasova, A. Misak, L. Kurakova, M. Grman, K. Ondrias, Characterization of rat cardiovascular system by anacrotic/dicrotic notches in the condition of increase/decrease of NO bioavailability. *Int. J. Mol. Sci.* **21**, 6685 (2020).

69. J. Hao, J. Wang, R. Shi, Q. Wang, X. Cheng, J. Feng, Y. Yang, Y. Hu, T. Chen, K. Chen, Cumulative resting heart rate load and cardiovascular risk in patients with heart failure in sinus rhythm. *Rev. Esp. Cardiol.* **79**, 204–214 (2026).

70. M. Skytioti, M. Elstad, Respiratory sinus arrhythmia is mainly driven by central feedforward mechanisms in healthy humans. *Front. Physiol.* **13**, 768465 (2022).

71. F. Shaffer, R. McCraty, C. L. Zerr, A healthy heart is not a metronome: An integrative review of the heart's anatomy and heart rate variability. *Front. Psychol.* **5**, 1040 (2014).

72. Q. Li, H. Huang, X. Lu, Y. Yang, Y. Zhang, W. Chen, W. Lai, G. Liang, S. Shi, X. Wang, J. Chen, S. Chen, X. Yan, The association between left ventricular end-diastolic diameter and long-term mortality in patients with coronary artery disease. *Rev. Cardiovasc. Med.* **24**, 84 (2023).

73. W. He, Q. Yao, D. Li, X. Sui, W. Zhang, Association between left ventricular remodeling and coronary chronic total occlusion in hypertensive coronary artery disease patients. *Sci. Rep.* **15**, 24239 (2025).

74. S. Jinno, A. Yamada, K. Sugimoto, J. Chan, C. Nakashima, Y. Funato, N. Hoshino, M. Hoshino, K. Takada, Y. Sato, H. Kawai, M. Sarai, H. Ito, H. Izawa, Resting echocardiographic parameters can exclude significant coronary artery disease: A comparison with coronary computed tomography angiography. *Echocardiography* **40**, 1251–1258 (2023).

75. Z.-Y. Liao, M.-C. Peng, C.-H. Yun, Y.-H. Lai, H. L. Po, C. J.-Y. Hou, J.-Y. Kuo, C.-L. Hung, Y.-J. Wu, B. E. Bulwer, H.-I Yeh, C.-H. Tsai, Relation of carotid artery diameter with cardiac geometry and mechanics in heart failure with preserved ejection fraction. *J. Am. Heart Assoc.* **1**, e003053 (2012).

76. H. L. Collins, R. A. Augustyniak, E. J. Ansorge, D. S. O'Leary, Carotid baroreflex pressor responses at rest and during exercise: Cardiac output vs. regional vasoconstriction. *Am. J. Physiol. Heart Circ. Physiol.* **280**, H642–H648 (2001).

77. P. J. Fadel, S. Ogoh, D. M. Keller, P. B. Raven, Recent insights into carotid baroreflex function in humans using the variable pressure neck chamber. *Exp. Physiol.* **88**, 671–680 (2003).

78. R. Manoj, K. V. Raj, P. M. Nabeel, M. Sivaprakasam, J. Joseph, Measurement of pressure dependent variations in local pulse wave velocity within a cardiac cycle from forward travelling pulse waves. *Sci. Rep.* **15**, 3066 (2025).

79. Z. Qiu, X. Chen, Y. Li, K. Chen, X. Ge, H. Xu, "Three-dimensional whole heart shape reconstruction for wearable ultrasound patches: A deep learning approach and experimental study," in *Functional Imaging and Modeling of the Heart*, R. Chabiniok, Q. Zou, T. Hussain, H. H. Nguyen, V. G. Zaha, M. Gusseva, Eds. (Springer, Cham, 2025), vol. 15673.

80. J. Alastruey, K. H. Parker, J. Peiró, S. M. Byrd, S. J. Sherwin, Modelling the circle of Willis to assess the effects of anatomical variations and occlusions on cerebral flows. *J. Biomech.* **40**, 1794–805 (2007).

81. F. Liang, K. Fukasaku, H. Liu, S. Takagi, A computational model study of the influence of the anatomy of the circle of willis on cerebral hyperperfusion following carotid artery surgery. *Biomed. Eng. Online* **10**, 84 (2011).

82. A. R. Lyon, T. López-Fernández, L. S. Couch, R. Asteggiano, M. C. Aznar, J. Bergler-Klein, G. Boriani, D. Cardinale, R. Cordoba, B. Cosyns, D. J. Cutter, E. de Azambuja, R. A. de Boer, S. F. Dent, D. Farmakis, S. A. Gevaert, D. A. Gorog, J. Herrmann, D. Lenihan, J. Moslehi, B. Moura, S. S. Salinger, R. Stephens, T. M. Suter, S. Szmit, J. Tamargo, P. Thavendiranathan, C. G. Tocchetti, P. van der Meer, H. J. H. van der Pal, ESC Scientific Document Group. 2022 ESC Guidelines on cardio-oncology developed in collaboration with the European Hematology Association (EHA), the European Society for Therapeutic Radiology and Oncology (ESTRO) and the International Cardio-Oncology Society (IC-OS): Developed by the task force on cardio-oncology of the European Society of Cardiology (ESC). *Eur. Heart J.* **43**, 4229–4361 (2022).

83. J. C. Plana, M. Galderisi, A. Barac, M. S. Ewer, B. Ky, M. Scherrer-Crosbie, J. Ganame, I. A. Sebag, D. A. Agler, L. P. Badano, J. Banchs, D. Cardinale, J. Carver, M. Cerqueira, J. M. DeCara, T. Edvardsen, S. D. Flamm, T. Force, B. P. Griffin, G. Jerusalem, J. E. Liu, A. Magalhães, T. Marwick, L. Y. Sanchez, R. Sicari, H. R. Villarraga, P. Lancellotti, Expert consensus for multimodality imaging evaluation of adult patients during and after cancer therapy: A report from the American Society of Echocardiography and the European Association of Cardiovascular Imaging. *J. Am. Soc. Echocardiogr.* **27**, 911–939 (2014).

84. X. Ge, Y. Liu, Z. Yin, S. Tu, Y. Fan, Y. Vassilevski, S. Simakov, F. Liang, Comparison of instantaneous wave-free ratio (iFR) and fractional flow reserve (FFR) with respect to their

sensitivities to cardiovascular factors: A computational model-based study. *J. Interv. Cardiol.* **11**, 4094121 (2020).

85. X. Gao, X. Chen, M. Lin, W. Yue, H. Hu, S. Qin, F. Zhang, Z. Lou, L. Yin, H. Huang, S. Zhou, Y. Bian, X. Yang, Y. Zhu, J. Mu, X. Wang, G. Park, C. Lu, R. Wang, R. S. Wu, J. Wang, J. Li, S. Xu, A wearable echomyography system based on a single transducer. *Nat. Electron.* **7**, 1035–1046 (2024).

86. H. Hu, X. Zhu, C. Wang, L. Zhang, X. Li, S. Lee, Z. Huang, R. Chen, Z. Chen, C. Wang, Y. Gu, Y. Chen, Y. Lei, T. Zhang, N. Kim, Y. Guo, Y. Teng, W. Zhou, Y. Li, A. Nomoto, S. Sternini, Q. Zhou, M. Pharr, F. L. di Scalea, S. Xu, Stretchable ultrasonic transducer arrays for three-dimensional imaging on complex surfaces, *Sci. Adv.* **4**, eaar3979 (2018).

87. C. Wang, B. Qi, M. Lin, Z. Zhang, M. Makihata, B. Liu, S. Zhou, Y. H. Huang, H. Hu, Y. Gu, Y. Chen, Y. Lei, T. Lee, S. Chien, K. I. Jang, E. B. Kistler, S. Xu, Continuous monitoring of deep-tissue haemodynamics with stretchable ultrasonic phased arrays, *Nat. Biomed. Eng.* **5**, 749–758 (2021).
